# From Contact to Stalemate: MAPK-Associated Chemical and Enzymatic Defenses Shape a Stable Barrage in the Co-Culture of *Trametes* sp. D and *Aspergillus niger* L14

**DOI:** 10.3390/jof12050327

**Published:** 2026-04-30

**Authors:** Jialiang Ying, Huawei Zhang

**Affiliations:** 1 School of Pharmaceutical Sciences, Zhejiang University of Technology, Hangzhou 310014, China; 18058765705@163.com; 2 State Key Laboratory of Green Chemical Synthesis and Conversion, Zhejiang University of Technology, Hangzhou 310014, China; 3 Zhejiang-Egypt Joint Laboratory on Intelligent Discovery of Marine Drugs, Zhejiang University of Technology, Hangzhou 310014, China

**Keywords:** co-culture, secondary metabolites, mechanisms, MAPK

## Abstract

The co-culture between *Trametes* sp. D and *Aspergillus niger* L14 resulted in a distinct orange-brown antagonistic band at their interface. Direct hyphal contact was associated with markedly enhanced production of numerous secondary metabolites (SMs), some of which were absent or decreased in monocultures. *T.* sp. D induced indolic compounds and cyclic dipeptides, such as Indole-3-acetamide and Cyclo-(Pro-Phe), whereas *A. niger* L14 overproduced polyketide-derived pigments and organic acids, such as Fonsecin and Kojic acid. These SMs did not inhibit their producer but suppressed the opponent’s growth, indicating reciprocal chemical antagonism. Transcriptomic analysis revealed upregulation of stress-related and metabolic genes, consistent with each fungus activating defense pathways. Biochemical assays showed that the confrontation zone had the highest oxidative stress markers, cell wall-degrading enzyme activity, and acidification (notably by *A. niger* L14), reflecting intense interfungal antagonism. The stress-response mitogen-activated protein kinase (MAPK) pathway was also activated in both fungi. Our findings supported a mechanistic model of fungal competition involving direct contact, chemical exchange, enzymatic attack, and stress signaling, highlighting that physical interactions likely contributed to triggering cryptic secondary metabolism and robust defense responses.

## 1. Introduction

Fungi commonly engage in antagonistic interactions in their natural habitats, competing for territory and resources. When two fungal colonies meet on a solid substrate, they often form a confrontation zone, which is a region of altered growth or a visible demarcation line at the interface [1]. Such confrontation or barrage lines are typically darkly pigmented boundaries, thought to result from the accumulation of defensive secondary metabolites (SMs) or pigments at the interface of the interaction. Depending on the species and their competitive strategies, the outcomes of fungal pairings could vary: one fungus may overgrow and replace the other, or both may stop spreading upon contact, forming a deadlock [2]. A recent systematic study of 105 pairwise interactions reported complete overgrowth or replacement in about one-third of cases. In contrast, nearly 44% of interactions led to mutual growth inhibition, with stable barrage zone formation [3]. These macroscopic outcomes underscore that a complex biochemical standoff underlies the confrontation zone. Unraveling the mechanisms behind such zones is essential, as fungal antagonism in nature influences community structure and could be harnessed in biotechnology, such as to induce new SMs [4]. Indeed, the competition-induced stresses triggered unique physiological responses and chemical outputs in fungi as they contended with one another.

Recent research has provided mechanistic insights into how fungi sense and respond during confrontation. Physical contact often triggers drastic cellular changes: hyphal tips may exhibit granulation, swelling, vacuolation, or burst upon encountering a competitor. At the colony level, this correlates with phenomena like growth inhibition at a distance or the formation of pigmented zone lines, as described above. On the biochemical front, SM induction is a hallmark of fungal antagonism. Co-culture studies showed that fungi in confrontation frequently upregulate otherwise silent biosynthetic gene clusters (BGCs), leading to the production of novel or enhanced SMs with antimicrobial activity [5]. Recent studies using defined two-fungus co-culture systems further support this view: co-culture of *A. alabamensis* with *A. fumigatiaffinis* was shown to induce defensive SMs under co-culture conditions [6], and a recent review likewise summarized fungal co-culture as an effective strategy for awakening silent BGCs and discovering cryptic SMs [7]. Moreover, new polyketides and alkaloids have been detected exclusively in the confrontation zones of competing fungi, supporting the idea that microbial interactions “turn on” cryptic metabolite pathways [8]. In parallel, fungi deploy enzymatic defenses and offenses. Antagonists such as *Trichoderma* spp. are well known to secrete lytic enzymes (such as Chitinases, Glucanases, Proteases) when in contact with other fungi, which can degrade the competitor’s cell wall [9]. Likewise, wood-decay basidiomycetes increased the production of extracellular oxidative enzymes, such as Laccases and Peroxidases, during combat, both to digest substrates and to generate toxic quinones or peroxide radicals against their opponents [2]. Thus, the antagonistic zone was an arena of intense metabolic crosstalk: physical contact-mediated signals, SMs, lytic enzymes, and oxidative stress all intertwined to determine whether one fungus gained the upper hand or a stalemate ensued.

*Trametes* species, such as *T. versicolor*, are renowned for their robust lignin-degrading enzyme systems and have been widely exploited in environmental biotechnology [10]. *T. versicolor* is also one of the most studied medicinal mushrooms because it produces polysaccharide-K and other immunomodulatory compounds used as adjuvant cancer therapies, and displays antibacterial, antioxidant, and anti-inflammatory activities [11]. *Aspergillus*, by contrast, includes both boon and bane species in human affairs. On the industrial side, *Aspergillus* spp. are workhorse microbes: *A. oryzae* has been safely used for centuries in food fermentation and is now a model host for enzyme production due to its high secretion capacity [12]. Likewise, *A. niger* is employed at scale for organic acid production and various enzymes [13]. Concurrently, *A. fumigatus* and *A. flavus* are among the most essential human fungal pathogens, causing invasive aspergillosis, especially in immunocompromised patients [14]. Moreover, *A. flavus* could produce carcinogenic aflatoxins, causing agricultural losses and posing health hazards. Given this dual significance (*Trametes* spp. in biodegradation and medicine, and *Aspergillus* spp. in industry and disease), understanding their antagonistic interaction is of high interest.

In this study, *T.* sp. D and *A. niger* L14 were co-cultured, and a multi-faceted approach was employed to dissect the mechanism of the antagonistic zone formation. Hyphal interactions at the interface were examined by microscopy. Metabolomics, coupled with metabolite isolation and structural characterization, was used to identify SMs and key bioactive compounds induced by confrontation. Then, transcriptomic analysis revealed differential gene expression during co-culturing, associated with SM biosynthesis, stress response, and other processes. The enzyme activities and stress-related biochemical markers were further quantified to elucidate mechanisms underlying fungal antagonism. Overall, this work sheds light on the complex interplay of secondary metabolism and signaling in fungal-fungal antagonism, providing a framework for understanding similar interactions in other microbes and informing potential biotechnological applications.

## 2. Materials and Methods

### 2.1. Fungal Strains, Culture Conditions and Analysis of SMs

This study was conducted with two fungal strains: fungus A, *Trametes* sp. D, a slower-growing basidiomycete endophyte isolated from *Edgeworthia chrysantha* (NCBI BioProject PRJNA1401684); fungus B, a faster-growing marine sponge-derived fungus, *Aspergillus niger* L14 (NCBI BioProject PRJNA669564). The preparation method for wheat bran leachate agar medium (MDA) was as follows: boiling 200 g of wheat bran (Hangzhou Huichuang Instrument Equipment Co., Ltd., Hangzhou, China) in 1 L of water for 20 min, and filtering the mixture, and then, adding 20 g of glucose (Sangon Biotech Co., Ltd., Shanghai, China) and 15–20 g of agar (Sinopharm Chemical Reagent Co., Ltd., Shanghai, China) to the filtrate and pouring them into 90 mm plates while still hot and allowing them to solidify to create the final MDA.

At first, *T.* sp. D was inoculated onto one side of a 9 cm MDA plate and incubated at 30 °C for 4–5 days until its colony reached approximately 3 cm in diameter. Subsequently, *A. niger* L14 was inoculated on the opposite side of the plate, and co-incubation was continued at 30 °C. After 2–3 days of dual cultivation, the two fungal colonies came into contact; within an additional 3–4 days, a distinct antagonistic interaction zone developed at their interface. Agar from the antagonistic zone was excised and extracted with ethyl acetate (Shanghai Lingfeng Chemical Reagent Co., Ltd., Shanghai, China) under ultrasonication to obtain the co-culture crude extract. Agar was cut from the areas of the plate furthest from the antagonistic zone to serve as monoculture controls, and the same extraction method was used to obtain individual control extracts for each fungus. MDA culture medium without fungal strains was used as a blank control. In addition, the two fungal strains were separated by a polycarbonate membrane (PC; Xiamen Nengxiang Technology Co., Ltd., Xiamen, China) placed in the center of the MDA plate (PC pore size of 0.22 µm, allowing compounds with molecular weight less than 2000 Da to pass through), and the remaining culture and extraction conditions were the same as those of the co-culture conditions, serving as a control group without physical contact. All extracts were filtered to remove particulate matter.

Each crude extract was diluted to 1.0 mg/mL in methanol (Sinopharm Chemical Reagent Co., Ltd., Shanghai, China) and analyzed by ultra-high-performance liquid chromatography-quadrupole time-of-flight tandem mass spectrometry (UHPLC-QTOF-MS/MS). The system consisted of a SCIEX X500B QTOF mass spectrometer coupled with a SCIEX ExionLC™ AC UHPLC (SCIEX, Framingham, MA, USA). Chromatographic separation was achieved on an Agilent ZORBAX Extend-C18 column (2.1 mm × 100 mm, 1.8 µm particle size; Agilent Technologies, Santa Clara, CA, USA) using a mobile phase of water (solvent A) and methanol (solvent B). The elution gradient was programmed as follows: 10% B at 0.00 min linearly ramping to 95% B over 20.00 min, holding at 95% B from 20.00 to 25.00 min, then returning to 10% B by 25.10 min and re-equilibrating at 10% B until 30.00 min. The flow rate was 0.30 mL/min, the column temperature was maintained at 35 °C, and the injection volume was 1 µL. The mass spectrometer operated in positive electrospray ionization mode with information-dependent acquisition for MS/MS fragmentation. High-resolution MS/MS spectra (product ion scans) were collected over an m/z range of 50–1500. Raw data files were processed using MZmine (v2.53), and MS-DIAL (v4.70) for feature detection and alignment, followed by compound analysis and annotation.

### 2.2. Scanning Electron Microscopy (SEM) Observations

SEM was used to observe hyphal interactions in the antagonistic zone of direct-contact co-cultures, using monoculture groups as controls. For the SEM experiments, small agar blocks containing the interaction zone were fixed in glutaraldehyde, dehydrated through an ethanol series, critical-point dried, sputter-coated with gold, and observed under a scanning electron microscope to visualize hyphal morphology and any physical interactions or structural changes at the interface. Observations were performed using a ZEISS scanning electron microscope (Carl Zeiss Microscopy GmbH, Jena, Germany).

### 2.3. Isolation and Identification of SMs

To obtain SMs from the co-culture antagonistic zone and monoculture, about 1000 MDA plates were scaled up for fermentation and extracted with ethyl acetate. Crude extracts were concentrated under reduced pressure. The extracts from the three groups were separated into monomeric compounds using Medium-pressure Liquid Chromatography (MPLC) and High-performance Liquid Chromatography (HPLC). Purified compounds were characterized by spectroscopic methods (mass spectrometry, nuclear magnetic resonance) to determine their structures.

### 2.4. Transcriptome Sampling and Sequencing

To capture gene expression changes during co-culture antagonism, RNA sequencing (RNA-seq) was conducted for both fungi under interaction and control conditions. Three conditions were sampled for transcriptomic analysis: monoculture of *T.* sp. D, monoculture of *A. niger* L14, and co-culture with direct contact. Total RNA was isolated using the RNeasy Mini Kit (Qiagen, Hilden, Germany) by Sinotech Genomics Co., Ltd. (Shanghai, China) according to the manufacturer’s standard protocol. RNA quantity and quality were checked, and mRNA was enriched. cDNA libraries were prepared and sequenced on the DNBSEQ-T7 platform (MGI Tech Co., Ltd., Shenzhen, China) to obtain paired-end reads. High-quality reads were aligned to reference genomes or transcriptomes of *T.* sp. D and *A. niger* L14. Differential expression analysis was performed to compare gene expression in co-culture and monoculture for each fungus. Functional enrichment analysis was then carried out: Gene Ontology (GO) enrichment to categorize the biological processes, cellular components, etc., and Kyoto Encyclopedia of Genes and Genomes (KEGG) pathway analysis to see which metabolic or signaling pathways were significantly enriched among the differentially expressed genes (DEGs).

### 2.5. Antagonistic Bioassays of Isolated Compounds and Metabolic Profiling

To evaluate the bioactivity of the isolated antagonistic zone SMs, the fungal growth inhibition assays were conducted. The producing strain for each compound was determined by comparing SMs from monoculture groups or by the results of transcriptomic analysis. Each purified compound was individually tested against both fungi. Solutions of each compound were prepared and added to the MDA culture medium to create media with different concentrations (1, 10, 100 μg/mL). In addition, a mixture of compounds was used to simulate the metabolic environment during co-cultivation: the mixture contained all the primary SMs obtained from either *T.* sp. D or *A. niger* L14 (at concentrations equivalent to those measured for each compound during co-culture). Then, spores and mycelium from each fungus were inoculated onto fresh MDA plates to observe strain growth. For *T.* sp. D, the growth-inhibition rates of the compounds were evaluated by measuring colony diameter. For *A. niger* L14, the 96-well plate method was used. The strain culture medium without the compounds served as a control. On the other hand, by extracting crude extracts from each group and analyzing them by liquid chromatography-mass spectrometry (LC-MS), the effects of each compound or mixture on the metabolic profile of the inhibited fungus were assessed, allowing inferences about the metabolic changes induced by the compounds in the target fungus.

### 2.6. Transcriptomics-Based Network Analysis

To investigate how SMs produced by one fungal strain affect another strain in the co-culture environment, a method similar to network pharmacology was employed, drawing on techniques used for drug target prediction. For example, for each key SM produced by fungus A: *T.* sp. D, the PharmMapper server (v2017) was used to identify candidate target proteins for each compound. Because PharmMapper’s database is largely based on human proteins, the output was curated to focus on enzymes or proteins with homologs in the fungus B: *A. niger* L14, as inferred from whole-genome data. This study assumed that if a compound from fungus A affected fungus B, fungus B might respond by altering the expression of target and related pathway genes. Then, the STRING database (v12.0) was used to explore protein–protein interactions and pathways: the list of fungal B genes from the predicted targets and the list of fungal B genes showing significantly upregulated expression in transcriptome data were uploaded to STRING to examine their interactions and clustering into networks. Additionally, the upregulated genes in each fungus were annotated with KEGG pathway information. Gene and protein terms, together with their corresponding genome annotation identifiers, where applicable, and predicted/annotated functions, were provided in Table S1.

### 2.7. Molecular Docking Analysis

To further validate and explore interactions between the SMs of one fungus and the key enzymes of the other, the molecular docking simulations were conducted. All enzymes from *A. niger* for which 3D structures were available in the Protein Data Bank (PDB), including computed structure models from AlphaFold DB and ModelArchive, were downloaded (prioritizing structures with a resolution better than 2 Å), and for *T.* sp. D, the data for *T. versicolor* was downloaded for reference. Each selected SM (ligand) was energy-minimized and converted to an appropriate 3D format. Docking was performed using CB-Dock2 based on AutoDock Vina (v1.1.2). Docking scores and binding poses were analyzed to assess affinity and interaction mode. A docking score less than −5 kcal/mol indicated good binding affinity, and the lower the score, the stronger the binding affinity [15]. Molecular docking analysis was used to support whether the SMs could feasibly interact with and disrupt the biological functions of the opposing fungus’s proteins.

### 2.8. Biochemical Indicator and Enzyme-Linked Immunosorbent Assay (ELISA) Test

To measure the intensity of cellular damage and stress responses in different spatial regions of the co-culture, the co-culture plates were divided into five zones: (1) the far side of *T.* sp. D colony (far from the antagonistic zone), representing unchallenged *T.* sp. D growth; (2) the near-interaction side of *T.* sp. D (the region of *T.* sp. D colony close to the antagonistic zone); (3) the antagonistic zone (the interface region containing both fungi); (4) the near-interaction side of *A. niger* L14 (the region of *A. niger* L14 colony close to the antagonistic zone); (5) the far side of *A. niger* L14 colony (far from the antagonistic zone), representing unchallenged *A. niger* L14 growth. In addition, two fungal strains were grown independently on fresh MDA plates as a control. The assays for each indicator were performed according to the instructions provided with the respective biochemical detection kits. For cell membrane damage indicators, the activity of extracellular Lactate dehydrogenase (LDH) and the content of intracellular malondialdehyde were measured. To assess biofilm damage, total extracellular polysaccharides were quantified. To detect cell wall damage, the extracellular activities of β-1,3-glucanase and chitinase were gauged. For intracellular oxidative stress situations, the enzyme activities of superoxide dismutase (SOD), catalase (CAT), and glutathione peroxidase (GSH-Px) were detected.

To assess whether the MAPK stress signaling pathways were engaged during fungal confrontation, the focus was placed on the p38 MAPK homologs in each fungus. In many fungi, the p38 MAPK equivalent is part of the high osmolarity glycerol (HOG) pathway [16]. In *T.* sp. D and in *A. niger* L14, similar stress-activated MAPK pathways exist. Protein extracts were prepared from the mycelium using a filamentous fungal protein extraction kit (BB-3136; Shanghai Beibo Biotechnology Co., Ltd., Shanghai, China): monoculture groups, co-culture groups, and the groups where one fungus was inoculated with a mixture containing all the primary SMs obtained from another fungus. After protein quantification, ELISA kits (ADY36325 and ADY36397; Shanghai Aodaying Biotechnology Co., Ltd., Shanghai, China) were used to detect activation of the p38/HOG pathway, including the phosphorylated and total forms of the protein. The ratio of phosphorylated protein to total protein was calculated to assess changes in this conserved stress signaling cascade during co-culture.

### 2.9. Determination of the pH Value in the Culture Medium

Since *A. niger* is known for acid production [17], changes in medium pH during the interaction were tracked as another factor in the antagonism. pH was measured at three time points and five zones from the co-culture plates: (i) before the fungi made contact; (ii) one day after initial contact (early interaction); (iii) four days after sustained contact, using the fresh MDA plates with two monoculture fungal strains as a control. At each time point, small agar samples were taken from several zones and immediately homogenized in distilled water. After centrifugation, the pH of the homogenate was measured with a calibrated pH electrode, using distilled water as a reference.

### 2.10. Integrated Metabolomic-Transcriptomic Pathway Analysis

To hypothesize biosynthetic pathways for the observed co-culture-specific SMs, metabolite and transcriptomic data were combined. For each SM uniquely produced or highly upregulated in co-culture, the transcriptome of the producing fungus was searched for candidate genes or gene clusters responsible for its biosynthesis. Pearson and Spearman correlation analyses were performed in Python (v3.9.13) to find correlations between SM abundance and gene expression levels (fragments per kilobase of exon model per million mapped fragments (FPKM) values and fold-change in co-culture and monoculture). High correlation coefficients and low *p*-values for a metabolite-gene pair suggested a possible link, thereby filtering the candidate biosynthetic genes or enzymes to estimate the biosynthesis mechanism of SMs.

### 2.11. Data Visualization and Statistical Analysis

Network analysis results were visualized and analyzed using Cytoscape (v3.9.1). The various quantitative analysis results were visualized using GraphPad Prism (v10.1.2; GraphPad Software, Boston, MA, USA).

## 3. Results

### 3.1. Physical Contact Was Associated with Antagonistic Interaction Zone Formation

**Figure 1:**
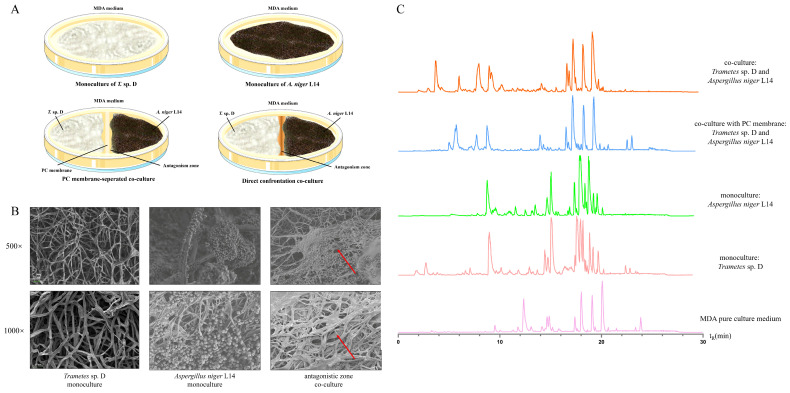
Direct contact-associated formation of a confrontation interface and metabolic divergence in co-culture. (**A**) Schematic plate phenotypes on wheat bran leachate agar medium (MDA) showing monocultures of *T.* sp. D and *A. niger* L14, polycarbonate membrane (PC)-separated co-culture, and direct confrontation co-culture. A distinct orange-brown antagonism zone was evident only under direct confrontation. (**B**) Scanning electron microscopy (SEM) of hyphae in monoculture and at the confrontation interface; red arrows indicated physical-contact regions between hyphae in the antagonistic zone. (**C**) Liquid chromatography-mass spectrometry (LC-MS) analysis of extracts from five conditions.

In co-culture plates where *T.* sp. D and *A. niger* L14 grew toward each other, a distinct confrontation interface formed upon their mycelial contact (Figure 1A and Figure S1A). Initially appearing as a thin golden-yellow band, this interface zone intensified in color over time, becoming a conspicuous orange-brown band separating the two colonies. The growth of each fungus was restricted upon approaching this band, suggesting mutual antagonism, and neither could overgrow the other, leading to a static border. By contrast, in monocultures, each fungus grew unimpeded across the plate with no such color change.

SEM of the direct confrontation interface between *T.* sp. D and *A. niger* L14 revealed extensive hyphal entanglement, indicative of intimate physical antagonism. The characteristically broad hyphae of *T.* sp. D were observed coiling around and firmly contacting the thinner *A. niger* L14 hyphae, even encasing some of *A. niger* L14’s conidial structures (Figure 1B). At higher magnification, the interaction zone showed evident direct hyphal contact and interweaving between the two fungi. The broader hyphae of *T.* sp. D were observed around the thinner hyphae of *A. niger* L14, with locally denser entanglement near conidial structures. Compared with the respective monocultures, hyphae at the confrontation interface were arranged more densely, with more disordered orientation and spatial distribution, and with more obvious overlap and crossing. These observations indicated pronounced physical contact and hyphal entanglement during confrontation, and suggested that the interface morphology differed markedly from that of the respective monocultures.

To examine the contribution of direct hyphal contact to these effects, the PC was used to separate the two fungi while allowing chemical diffusion (Figure 1A and Figure S1B). In the membrane-separated co-culture plates, the growth of each fungus continued until it reached the membrane. Notably, in these divided plates, there was no prominent orange-brown band as seen in direct contact. A faint yellowish zone was sometimes observed on the membrane where the opposing metabolites met, but it was markedly lighter and narrower than the band in direct contact plates. These observations indicated that the antagonistic response, particularly pigment production at the interface, was markedly stronger under direct-contact conditions. Without direct contact between hyphae, interactions mediated solely by diffusible molecules would elicit only weak responses, suggesting that direct hyphal contact was an important contributing factor.

Subsequently, the SM profiles of the five groups were analyzed by LC-MS, using uninoculated MDA as a control to exclude interference from medium components (Figure 1C). Compared with the monoculture groups, the SM profiles of the co-culture group with direct contact showed very significant changes, with some product peak areas significantly increased and others absent in monoculture groups (e.g., 3–6 min, 8–11 min). However, in the co-culture group with physical separation, some SMs showed significant changes compared to the monoculture groups, but not as pronounced as in the direct contact group (e.g., 3–4 min, 9–14 min), indicating that although there was a PC for isolation, some SMs could still penetrate the PC during co-culture to affect the growth of the other fungus, but not as severely as in direct contact. These results further indicated that direct contact was strongly associated with these changes.

### 3.2. Isolation and Identification of SMs from Monocultures and Co-Culture

**Figure 2:**
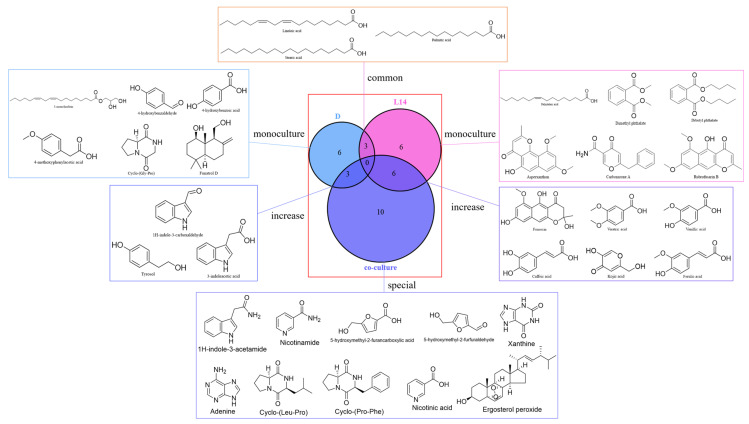
Summary of the 19 SMs isolated and identified across culture conditions, shown with a Venn-style classification of SMs associated with *T.* sp. D, *A. niger* L14, and co-culture induction and enrichment, alongside chemical structures.

**Figure 3:**
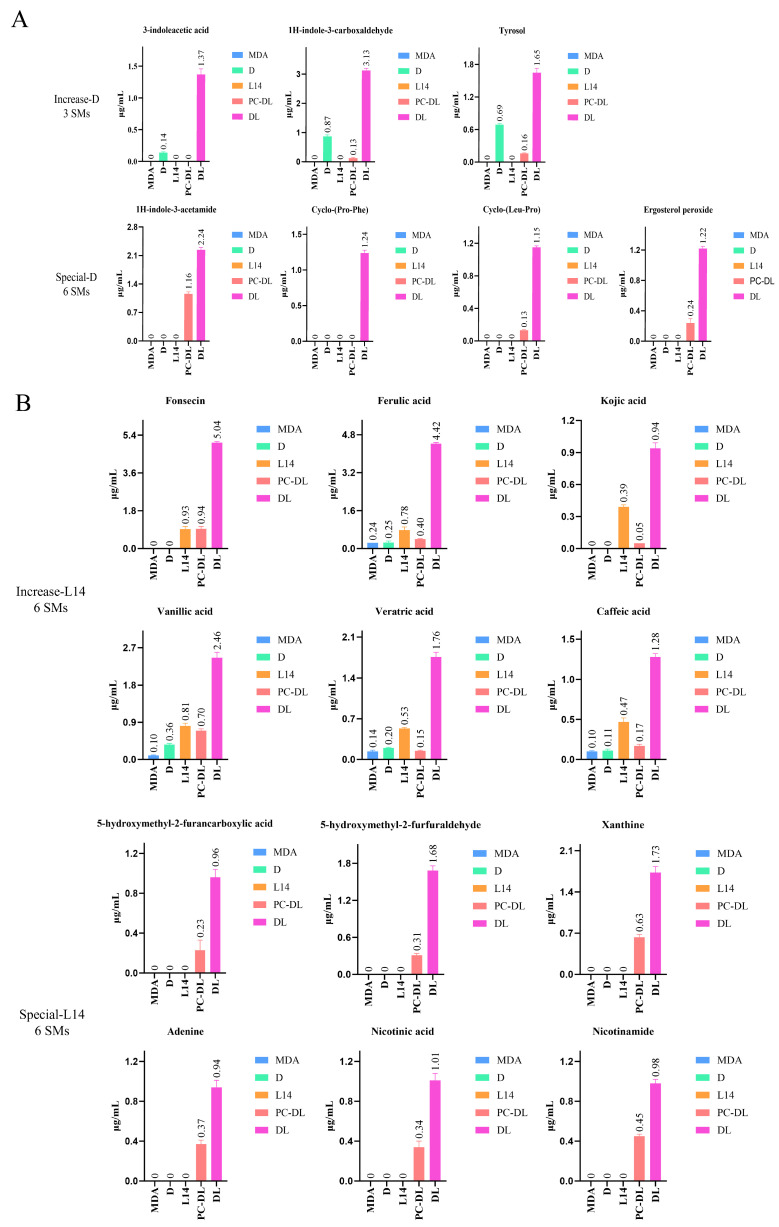
Induced or augmented SMs in co-culture. Abbreviations represent the growth conditions as follows: MDA, blank medium without fungi; D, monoculture of *T.* sp. D; L14, monoculture of *A. niger* L14; PC-DL, co-culture with PC; DL, co-culture without PC. (**A**) Quantification of 7 *T.* sp. D-derived SMs across 5 conditions. (**B**) Quantification of 12 *A. niger* L14-derived SMs across 5 conditions.

To clarify the chemical interactions between the two fungal strains during co-culture, 19 SMs were obtained from large-scale fermentation cultures using about 1000 MDA plates, followed by extraction of the antagonistic zone and isolation and identification of the SMs. Simultaneously, to determine their source strains, monomeric compounds were isolated from the extracts of each fungal strain cultured separately, and combined with the results of DEGs in the transcriptome, it was determined that *T.* sp. D and *A. niger* L14 produced 7 and 10 SMs, respectively. In addition, the content of each SM in different groups was analyzed using LC/MS. Some fatty acid compounds (e.g., Linoleic acid) were isolated from both strains in their monocultures, and some terpenoid or polyketide compounds (e.g., Funantral D, Asperxanthon) were isolated only from their respective monoculture. SMs from *T.* sp. D: several indole and aromatic SMs were isolated from the antagonistic zone that corresponded to *T.* sp. D-derived SMs (Figure 2). Three compounds were found at significantly higher levels in co-culture than in monoculture: 3-Indoleacetic acid, 1H-Indole-3-carboxaldehyde, and Tyrosol. In monoculture, their concentrations were low, but in the antagonistic zone, they increased severalfold, reaching levels approximately 2–10 times higher (Figure 3A). Strikingly, four special SMs were present in the antagonistic zone extracts that were not detected in monoculture: 1H-Indole-3-acetamide, cyclo-(Pro-Phe), cyclo-(Leu-Pro), and Ergosterol peroxide. 1H-Indole-3-acetamide is notable as a precursor or shunt metabolite in the 3-Indoleacetic acid biosynthetic pathway, suggesting *T.* sp. D upregulated tryptophan-derived secondary metabolism under competition [18]. Cyclo-dipeptides are a class of compounds often associated with microbial interactions and can exhibit antibiotic or signaling activities [19]. Ergosterol peroxide is a known antifungal and antibacterial compound; its production indicates oxidative modifications in *T.* sp. D’s sterol metabolism when challenged [20].

SMs from *A. niger* L14: *A. niger* likewise showed a broadened SM profile in co-culture, mainly organic acids and polyketones (Figure 2). Six compounds that were present in monoculture at low levels became highly enriched in co-culture with several-fold higher concentration: Fonsecin, Ferulic acid, Kojic acid, Vanillic acid, Veratric acid, and Caffeic acid (Figure 3B). Additionally, another set of six special SMs was uniquely detected in co-culture: 5-Hydroxymethyl-2-furfuraldehyde, 5-Hydroxymethyl-2-furancarboxylic acid, Xanthine, Adenine, Nicotinic acid, and Nicotinamide. The two furan derivatives were likely sugar degradation products, and their presence indicated altered carbohydrate metabolism or perhaps stress-induced breakdown of sugars in co-culture [21]. Xanthine and Adenine are purine derivatives, suggesting increased nucleotide turnover or stress-related release of nucleic acid components [22]. Nicotinic acid and Nicotinamide might be secreted due to perturbation of the NAD^+^/NADH cycle or as metabolic byproducts when *A. niger* L14 was under stress [23]. Interestingly, Nicotinic acid could also act as an antimicrobial agent in some contexts by interfering with other organisms’ metabolism [24]. These unique compounds represented co-culture-specific SMs of *A. niger* L14, indicating that *A. niger* L14 activated certain silent or lowly expressed pathways only when confronted by *T.* sp. D.

Next, the roles of these SMs during co-culture were investigated by testing their effects on the growth of the source strain and competing strains in a dose-dependent manner (Figure S2). The results indicated that none of the compounds significantly inhibited the growth of the fungus that produced them, even at the highest tested concentration of 100 μg/mL. *T.* sp. D continued to grow normally on media supplemented with its own SMs, and *A. niger* L14 was likewise not inhibited by its SMs at these levels. The results showed that all SMs exhibited clear inhibitory effects on the other fungus. For instance, *T.* sp. D-derived Ergosterol peroxide caused a dose-dependent reduction in *A. niger* L14 growth, but *T.* sp. D growth was unaffected. Similarly, *A. niger* L14’s pigment Fonsecin could impede *T.* sp. D growth, and this effect was dose-dependent, whereas *A. niger* L14 itself tolerated these SMs. These results suggested that each fungus had intrinsic resistance or detoxification mechanisms for its own SMs, but was vulnerable to the other’s chemical arsenal. In addition, when the mixture of *T.* sp. D-derived SMs was added to the *A. niger* L14 culture medium, *A. niger* L14 growth was markedly suppressed, mimicking the antagonistic effect of a live *T.* sp. D (Figure S3). Similarly, the mixture of *A. niger* L14-derived SMs added to *T.* sp. D plates caused obvious growth inhibition of *T.* sp. D. These experiments demonstrated that mixtures of SMs were the main groups of compounds produced by each fungus during co-culture, and that these compounds acted synergistically as chemical weapons to inhibit the growth of competing strains without harming themselves. This was an ingenious evolutionary strategy: each fungus could poison the microenvironment in a way that was only detrimental to competing strains.

Exposing one fungus to the SMs of another fungus could lead to changes in the metabolic profile of the target strain. Therefore, to further investigate the mechanisms of SM production during co-culture from a metabolomic perspective, product concentrations in each group were analyzed by LC-MS (Table S2). For example, treating *A. niger* L14 with *T.* sp. D’s indolic compounds (such as 3-Indoleacetic acid, 1H-Indole-3-carboxaldehyde) led to *A. niger* L14 producing a slight pigment and exuding organic acids, similar to what happened in co-culture. LC-MS analysis of *A. niger* L14 treated with the mixture of *T.* sp. D-derived SMs showed induction of some compounds (like Kojic acid), suggesting that *A. niger* L14 sensed those chemical signals and ramped up certain pathways. On the other hand, when *T.* sp. D was grown with *A. niger* L14’s SM mixture, Indole-3-acetamide, and other defensive or antifungal *T.* sp. D-derived compounds were detected even in the absence of *A. niger* L14, although the concentrations of these compounds were lower than during co-culture, indicating that *A. niger* L14’s chemicals alone could trigger some of *T.* sp. D’s response and physical contact could exacerbate these reactions. This provided evidence that SMs themselves function as signaling molecules or stressors that elicited counter-responses in the opposing fungus, suggesting that the isolated compounds were the main SMs used by the two strains for mutual defense and competition during co-culture, and the compound groups produced by one strain could induce the other strain to initiate and produce the corresponding compound groups.

### 3.3. Network Analysis Linking Specific SMs with Transcriptomic Data Revealed the Activation of Stress and Defense Pathways

To understand how each fungus responded to the environment during co-culture and how SMs produced by one fungus affected the other, a comprehensive transcriptomic analysis was performed. Simultaneously, drawing on principles of network pharmacology, networks were constructed that integrated the specific SMs of one fungus with the transcriptomic data of the other. Related genome annotation identifiers, where applicable, and predicted/annotated functions were summarized in Table S1.

**Figure 4:**
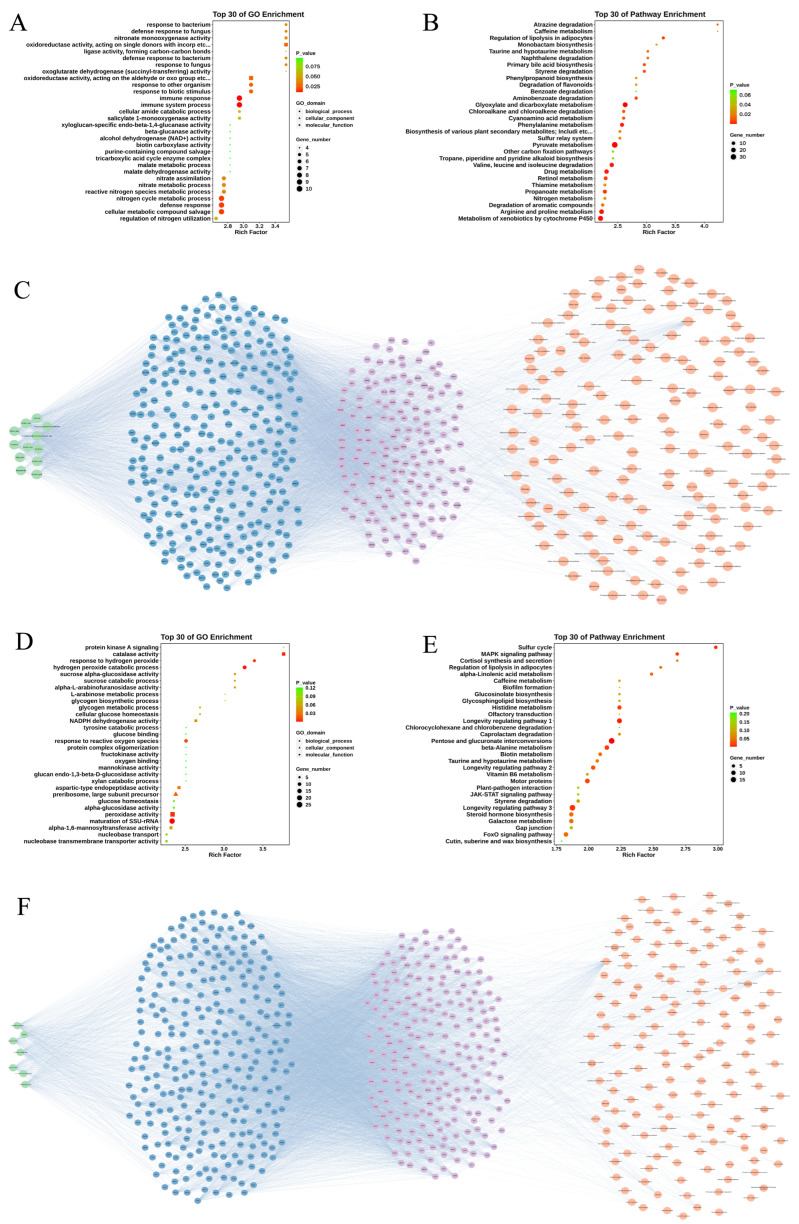
Transcriptome-based pathway enrichment and networks reveal coordinated offense and defense programs in both fungi. (**A**) Enriched GO terms in *T.* sp. D during co-culture. (**B**) Enriched KEGG pathways among *T.* sp. D during co-culture. (**C**) Interaction network linking the 12 *A. niger* L14-derived SMs to predicted gene targets and co-culture-upregulated annotated-KEGG genes in *T.* sp. D. (**D**) Enriched GO terms in *A. niger* L14 during co-culture. (**E**) Enriched KEGG pathways among *A. niger* L14 during co-culture. (**F**) Interaction network linking the 7 *T.* sp. D-derived SMs to predicted gene targets and co-culture-upregulated annotated-KEGG genes in *A. niger* L14. The symbol and color codes, including enrichment categories, *p* values, gene counts, and network node/edge types, were indicated in the embedded legends for the corresponding panels.

Under confrontational conditions, *T.* sp. D exhibited distinct defensive and offensive responses. Transcriptome analysis revealed that *T.* sp. D significantly upregulated a series of genes encoding extracellular degradative enzymes, including lignin-degrading enzymes such as Laccase, Chloroperoxidase, and Lignin peroxidase. This enhanced expression of oxidoreductases suggested that *T.* sp. D released greater oxidizing power in the confrontation zone, potentially enabling it to attack the hyphae of competing fungi and decompose their organic compounds. Simultaneously, genes encoding cell wall hydrolases, such as Chitinase, were also altered, with some isoenzymes significantly upregulated. Chitinase is generally associated with chitin degradation in fungal cell walls, suggesting that *T.* sp. D may attempt to weaken the structural integrity of *A. niger* L14 by targeting the opponent’s cell wall. The synergistic effect of these enzymatic weapons reflected a proactive offensive strategy of *T.* sp. D, namely, seizing survival space through enzymatic degradation and oxidative stress. In addition to its secretory offensive, *T.* sp. D also initiated detoxification and adaptation mechanisms against the SMs of its competitors. For example, *T.* sp. D upregulated genes annotated as oxalate decarboxylase, formate dehydrogenase, etc., suggesting a potential role in the degradation of organic acids secreted by the competing fungus. *A. niger* is known to readily consume large amounts of organic acids and obtain nutrients, or inhibit other organisms by acidifying its environment. Therefore, the increased activity of organic acid-degrading enzymes in *T.* sp. D was considered a defensive adaptation aimed at neutralizing the chemical weapons of *A. niger* L14. Based on the GO function and KEGG metabolic pathway enrichment analysis of DEGs, it was highlighted that *T.* sp. D significantly enriched genes related to the defense response against fungi (Figure 4A,B). When co-cultured with *A. niger* L14, *T.* sp. D showed enrichment of genes and pathways associated with responses to fungal challenge, including pathways potentially involved in recognizing non-self hyphae and triggering defense responses. The molecular functions of the DEGs in *T.* sp. D were mainly oxidoreductase activities, such as monooxygenase and peroxidase activities. This was consistent with the aforementioned enzyme analysis, suggesting that *T.* sp. D may extensively utilize oxidase-related systems to oxidize and decompose organic compounds and other toxic secretions in the antagonistic zone. Furthermore, some secondary metabolic pathways were also enriched in *T.* sp. D, such as the biosynthesis pathway of phenylpropanoid compounds, suggesting that *T.* sp. D mobilized the synthesis of SMs for environmental adaptation or antifungal effects.

Analysis of SMs from *A. niger* L14 affecting *T.* sp. D: using the PharmMapper database, it was found that the main SMs produced by *A. niger* L14 during co-culture were associated with multiple target enzymes in *T.* sp. D. Subsequently, the STRING database was used to analyze further the interactions between these target enzymes and the genes significantly upregulated in *T.* sp. D during co-culture, the KEGG pathways to which these genes belong were annotated, ultimately forming a coherent network (Figure 4C and Figure S4). The integrative network suggested that the 12 *A. niger* L14-derived SMs acted through many targets, with 1380 compound-target edges linking to 291 *T.* sp. D enzymes. Direct associations were multi-target and overlapping across SMs, indicating a distributed perturbation rather than a singular-mode effect. A core enzyme set was predicted for all 12 SMs: DUT (dUTPase; nucleotide sanitization), GALE (UDP-glucose 4-epimerase; carbohydrate metabolism), and PANC (pantothenate synthetase; CoA biosynthesis). Other key targets included PCK1 (gluconeogenic control), PIM1 (mitochondrial protease), and secreted glycosidases CBH1, ABFA, and FAEA, which aligned with *T.* sp. D’s extracellular hydrolytic enzyme arsenal. After linking these targets to the transcriptional upregulating response, a network of 182 targets, 175 genes, and 21,239 compound-target-gene edges across 158 KEGG pathways (score ≥ 0.15) was yielded. In this network, GALE remained prominent because it hit all SMs, and oxidative-stress nodes CTA1 (catalase) and GLR1 (glutathione reductase) emerged as hubs, among *T.* sp. D’s upregulated genes, hubs included *RAD51* (DNA repair), *SOD2*, *CAT* (reactive oxygen species, ROS detox), and *FDH* (formate dehydrogenase), reflecting genome maintenance, antioxidant defense, and organic-acid detoxification. The key enriched pathways involved core metabolism and redox functions (e.g., carbon metabolism, oxidative phosphorylation, peroxisome, glyoxylate/dicarboxylate metabolism). Overall, this network connected the *A. niger* L14-derived SMs to both *T.* sp. D’s offensive enzyme arsenal and its detoxification or redox defense in the antagonistic zone.

For *A. niger* L14, it also exhibited intense stress, attack, and defense response during co-culture. Firstly, *A. niger* L14 upregulated multiple antioxidant defense-related genes, particularly the catalase gene, which was significantly elevated. Catalase efficiently breaks down hydrogen peroxide and plays a crucial role in antioxidant defense. This change indicated that when *T.* sp. D produced peroxides and free radicals via its oxidases, *A. niger* L14 may have enhanced its ability to scavenge hydrogen peroxide, thereby helping to alleviate oxidative stress and protect cellular structures from damage. Secondly, *A. niger* L14 also modulated transmembrane transport and secondary metabolism processes to counter the attack by *T.* sp. D. Transcriptome data showed a strong upregulation of multiple membrane transporter genes during co-culture. These transporters were likely involved in exporting toxic SMs produced by *T.* sp. D out of the cell for detoxification and tolerance, and to improve their own nutrient uptake efficiency. Similarly, *A. niger* L14 secreted some antagonistic SMs to counteract *T.* sp. D, including organic acids and antifungal SMs. GO functional analysis revealed a high enrichment of hydrogen peroxide decomposition and oxidative stress response processes in *A. niger* L14, further demonstrating that *A. niger* L14 primarily defended against *T.* sp. D attacks by enhancing antioxidant capacity (Figure 4D). In particular, the molecular function of catalase activity reached a significant level in the enrichment analysis, indicating that multiple catalase-related genes were co-upregulated during co-culture. Meanwhile, the enrichment of multiple hydrolytic enzyme activities indicated that *A. niger* L14 may attack *T.* sp. D during co-culture through the secretion of hydrolytic enzymes. KEGG analysis revealed substantial alterations in signaling and metabolic pathways in *A. niger* L14 (Figure 4E). For example, genes related to the MAPK signaling pathway showed an upregulation trend, suggesting that *A. niger* L14 may modulate cellular activity and induce defense gene expression through stress signaling cascades. Furthermore, in terms of metabolic pathways, *A. niger* L14 exhibited varying degrees of enrichment and changes in pathways related to membrane lipids, amino acids, etc., during co-culture. These changes reflected extensive metabolic reprogramming in *A. niger* L14, including enhanced pathways required for defense and survival, and reduced energy-consuming growth-related pathways, to better adapt to competitive adversity.

Analysis of SMs from *T.* sp. D affecting *A. niger* L14: a complete analytical network was also constructed using the PharmMapper and STRING databases (Figure 4F and Figure S5). The integrated network with 7 *T.* sp. D-derived SMs identified 271 predicted targets linked by 776 compound-target edges. Two major functional axes emerged. Firstly, oxidative stress defense: e.g., 3-Indoleacetic acid targeted DAO1 and a catalase-like protein CATA, and 1H-Indole-3-acetamide also hit CATA, consistent with *A. niger* L14’s peroxide-detox response. The top hit for Tyrosol was SOD2, consistent with superoxide dismutase upregulation. Secondly, membrane or sterol and stress-sensing nodes appeared: Ergosterol peroxide strongly hit multiple targets (MUP1, CCP1, PIM1) and also scored IRE1, YAP1, and the sterol enzyme CYP51, suggesting interference with membrane integrity and stress signaling. In particular, fungal hydrolases were targeted. For example, Tyrosol scored CHI1 (Chitinase) and Cyclo-(Pro-Phe) scored LIP (lipase) and AXE1 (acetylesterase), indicating roles in cell-wall hydrolysis. After linking targets and transcriptome data, 237 target nodes, 217 upregulated genes, and 18,761 edges were yielded. This network explained the enriched pathways. For instance, YAP1 was linked to the ABC-transporter regulator PDR and SOD2, tying into detox pumps and antioxidant defense. CATA was linked to peroxisomal oxidases (e.g., KATG, CATB), matching the Peroxisome signature. In total, KEGG mapping of the 217 network-linked DEGs identified 151 pathways, including Peroxisome, Glutathione metabolism, ABC transporters, MAPK signaling, etc. (each involving multiple key genes). Overall, *T.* sp. D’s SMs act via multiple direct and indirect interactions to trigger the antioxidant, detoxification, membrane-repair, and hydrolytic pathways that dominate *A. niger* L14’s co-culture response.

### 3.4. Molecular Docking Revealed the Multi-Enzyme Binding Potential of Antagonism-Associated SMs

**Figure 5:**
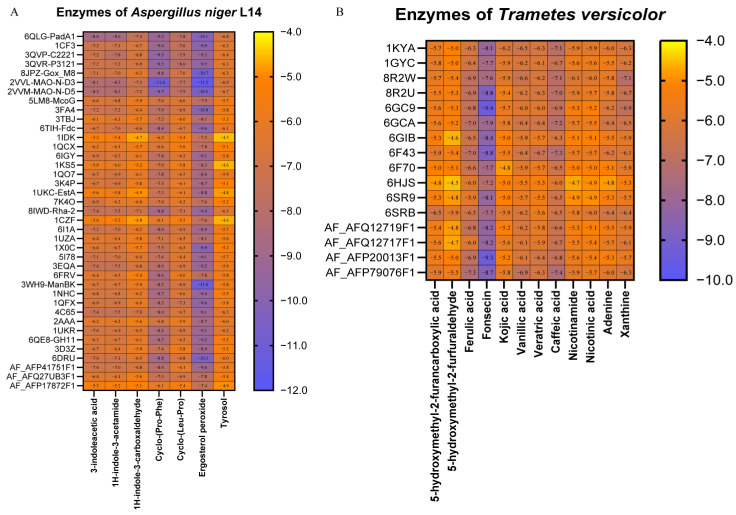
Molecular docking supported direct interactions between co-culture-induced SMs and antagonism-relevant enzymes. (**A**) Heatmap of predicted docking affinities between 7 *T.* sp. D-derived SMs and representative enzyme structures from *A. niger*. (**B**) Heatmap of predicted docking affinities between 12 *A. niger* L14-derived SMs and representative enzyme structures from *T. versicolor*.

Molecular docking studies provided evidence for the interactions between metabolites and target enzymes. Seven major SMs derived from *T.* sp. D were analyzed using the CB-Dock2 platform to examine their binding to all classes of enzymes from *A. niger* recorded in the PDB database (Figure 5A, Table S3). Docking provided structure-level support for the network’s core premise that *T.* sp. D-derived SMs had the physical potential to engage multiple *A. niger* enzymes. Across 39 enzyme structures (predominantly hydrolases and oxidoreductases), including 36 experimentally solved structures and 3 predicted models, Ergosterol peroxide and Cyclo-(Pro-Phe) exhibited the strongest global binding profiles, while Tyrosol showed comparatively weaker but still favourable binding. Using a stringent strong binding (score ≤ −5 kcal/mol), the results indicated that Ergosterol peroxide achieved 39 strong interactions (mean −9.051 kcal/mol), and Cyclo-(Pro-Phe) also achieved 39 pairs (mean −7.964 kcal/mol). 1H-Indole-3-acetamide (mean −6.797 kcal/mol) and 3-Indoleacetic acid (mean −6.785 kcal/mol) similarly displayed 39 strong interactions, followed by Cyclo-(Leu-Pro) (mean −6.613 kcal/mol). In contrast, 1H-Indole-3-carboxaldehyde exhibited 36 strong interactions (mean −6.131 kcal/mol), and Tyrosol showed 34 pairs (mean −5.723 kcal/mol). Representative high-affinity pairs further emphasised preferential engagement of oxidoreductases, consistent with the network’s oxidative-stress axis and *A. niger* L14’s observed upregulation of *CAT*, *CATB*, *SOD2* and glutathione-associated genes. Ergosterol peroxide demonstrated the strongest single docking score against monoamine oxidase N (2VVL-MAO-N-D3, −11.5 kcal/mol), and Cyclo-(Pro-Phe) bound the same enzyme with −11.0 kcal/mol; Tyrosol also bound MAO-N but relatively more weakly (−6.9 kcal/mol). The indole SMs displayed repeatable high affinity against flavin prenyltransferase PAD1 (6QLG-PadA1, −8.6 kcal/mol for both 3-Indoleacetic acid and 1H-Indole-3-acetamide), whereas Cyclo-(Leu-Pro) reached its best score against glucose oxidase (3QVR-P3121, −8.0 kcal/mol). Importantly, almost all SMs were strongly bound to hydrolytic enzymes, such as 6IGY (Chitinase), 3EQA (Glucoamylase), 5I78 (Endo-β-1,4-glucanase), consistent with *A. niger* L14 secreting a large amount of hydrolytic enzymes to attack *T.* sp. D during co-culture. Of note, molecular docking validated the feasibility of multi-enzyme binding and highlighted leading SMs [especially Ergosterol peroxide and Cyclo-(Pro-Phe)].

On the other hand, twelve major SMs derived from *A. niger* L14 were analyzed, and all enzyme classes from *T. versicolor* were recorded in the PDB database (Figure 5B, Table S4). Molecular docking corroborated the network-derived redox or detox and antagonism-chemistry results by showing that *A. niger* L14-derived SMs could form energetically favourable contacts with *T.* sp. D’s oxidoreductases and glutathione transferases. The docking dataset comprised 192 dockings (12 ligands × 16 enzymes), spanning experimentally resolved structures (two laccases, hydroxyquinol-1,2-dioxygenase, phenol 2-monooxygenase, and eight GST isoenzymes) and four predicted oxidoreductases (two laccases, ligninase C, and pyranose 2-oxidase [P2Ox]). This study found that over 93.75% docking scores were below −5 kcal/mol, indicating predicted interactions generally had strong binding forces. Across ligands, Fonsecin showed the strongest overall binding (mean −8.24 kcal/mol across the 16 enzymes) and produced the best single score (−9.4 kcal/mol) against a GST Xi isoform (GST Xi1, 6GC9). Additional high-affinity contacts included Fonsecin-ligninase C (−9.3 kcal/mol) and Fonsecin-phenol 2-monooxygenase (−8.8 kcal/mol), consistent with broad binding to detoxification-associated transferases and redox enzymes implicated in aromatic compound transformation. At the enzyme level, predicted P2Ox exhibited the most favourable average profile (mean −6.48 kcal/mol across ligands), and phenolic acids showed strong P2Ox binding (Caffeic acid-P2Ox was −7.4 kcal/mol, Ferulic acid-P2Ox was −7.3 kcal/mol). Kojic acid bound a Laccase structure at −6.2 kcal/mol, and aromatic-detoxification candidates also showed supportive binding (Nicotinamide and Nicotinic acid to hydroxyquinol-1,2-dioxygenase at −6.1 and −6.0 kcal/mol, respectively). These patterns were mechanistically coherent with the transcriptome described: *T.* sp. D upregulated extracellular oxidoreductases (e.g., Laccases, Peroxidases) and hydrolytic enzymes as offensive tools, while simultaneously activating detoxification capacity. Fungal GSTs are canonical detoxification enzymes that confer xenobiotic resistance and oxidative-stress protection, making strong GST docking contacts consistent with a detoxification interpretation [25]. In parallel, P2Ox has been proposed to support lignin-associated redox chemistry by generating hydrogen peroxide, an essential cosubstrate for lignin peroxidases in lignocellulolytic fungi. Thus, strong binding of phenolic acids and Fonsecin to P2Ox plausibly intersects the same oxidative axis that underpins *T.* sp. D’s offensively increased oxidising power in the antagonistic zone [26].

Overall, the docking results supported the idea that many SMs could directly bind to and potentially inhibit critical enzymes of the opposing fungus. These include enzymes involved in pathways such as cell membrane synthesis, cell wall maintenance, metabolic energy production, and protective enzyme systems. In short, these docking results supported the plausibility of specific compound-target interactions that may contribute to the observed physiological and transcriptomic changes, rather than attributing these changes solely to general physical contact stress.

### 3.5. Stress and Signal Transduction at the Fungal Interface: Oxidative or Cell Wall Damage and Activation of the MAPK Signaling Pathway

**Figure 6:**
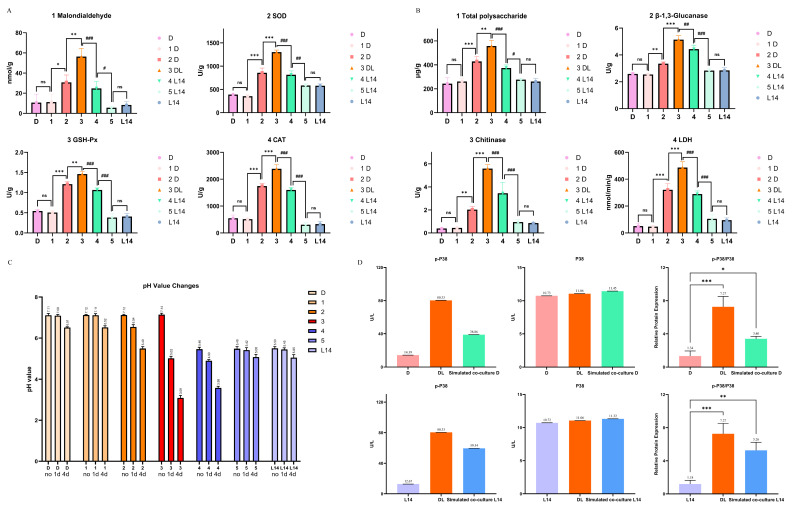
Spatial physiological stress patterns across the confrontation zone and activation of p38/HOG-MAPK signaling pathway. (**A**) Oxidative/stress-associated and antioxidant indicators were measured from mycelia sampled across monoculture controls and co-culture spatial regions. (**B**) Interface-associated matrix/cell-wall and damage-related indicators. (**C**) pH measurements across time (0, 1, and 4 d) in monoculture and spatial zones of the co-culture plate. (**D**) Enzyme-linked immunosorbent assay (ELISA)-based quantification of phosphorylated and total p38/HOG-MAPK in each fungus under monoculture, direct co-culture (DL), and simulated co-culture conditions. The p-p38/p38 ratio summarizes pathway activation. Data were presented as mean ± SD (*n* = 3). Statistical differences among groups were evaluated by one-way ANOVA. Asterisks indicated significant differences compared with the *T.* sp. D monoculture, and hash symbols indicated significant differences compared with the *A. niger* L14 monoculture. * *p* < 0.05, ** *p* < 0.01, *** *p* < 0.001 vs. D; ^#^ *p* < 0.05, ^##^ *p* < 0.01, ^###^ *p* < 0.001 vs. L14; ns, not significant.

Initially, the biochemical indicators of five zones on the MDA plates during co-culture were detected. It was found that the values of all indicators were highest in the antagonistic zone and decreased gradually from there to the outer zones. Additionally, the content of various indicators in the remote zones was not significantly different from that in monocultures (Figure 6A,B). For cell membrane damage, LDH release was lowest in the far regions of each fungus, around zones 1 and 5, indicating intact cells and minimal lysis there. As the interface approached, LDH levels rose. In zones 2 and 4, LDH was moderately elevated. Strikingly, in zone 3, LDH activity spiked to about 5–6 times the baseline. This indicated that substantial cell membrane damage occurred at the site of fungal contact. In addition, the malondialdehyde content reflected lipid peroxidation and showed a similar trend: minimal in the far zones, higher near the interface, and highest at the interface. These data confirmed that the antagonistic zone was a hotspot of cell membrane injury, likely due to the action of antifungal compounds and ROS produced by each fungus against the other. The amount of extracellular polysaccharides was measured as an indicator of biofilm or protective matrix. The results showed that the total polysaccharide was lowest in the far zones of both fungi and increased towards the antagonistic zone. This suggested that both fungi secrete more polysaccharides in the battle zone, possibly to form a barricade or immobilize the opponent’s enzymes. For instance, *T.* sp. D might produce glucans to thicken the antagonistic zone, and *A. niger* L14 might produce polysaccharide granules as a defence.

For cell wall-degrading enzymes: both β-1,3-Glucanase and Chitinase activities were significantly higher at the confrontation interface than elsewhere. In zone 3, β-1,3-Glucanase activity was approximately 2–3 times that in the far zones. Chitinase activity showed an even more pronounced difference: very low in zones 1 and 5, but rising sharply in zones 2 and 4, especially in zone 3. The near-interface zones of each fungus showed intermediate levels of these enzymes, suggesting that some enzymes diffused or that those cells were primed for attack. This pattern indicated a localized deployment of these enzymes at the battlefront: both fungi were likely releasing them that could break down the other’s cell wall. The synergistic presence of both enzymes in the same zone was reminiscent of biological control mechanisms (Chitinases and Glucanases together degrade fungal invaders). The fact that each fungus produced minimal enzymes in isolation but abundant ones at the interface underscores that this was a responsive attack mechanism induced by confrontation.

Regarding oxidative stress, the activities of SOD, CAT, and GSH-Px were uniformly low in the peaceful far zones. As one moved toward the interface, all three enzyme activities rose, with the peak at zone 3. SOD activity in the band was roughly 2–3 times that in zones 1 and 5, CAT was nearly 6–8 times, and GSH-Px was also significantly elevated. This corresponded well with the high oxidative stress, as evidenced by malondialdehyde at the interface. The organisms were actively countering ROS by ramping up antioxidant defenses. Interestingly, zones 2 and 4 also showed elevated SOD and CAT levels, though not as high as in zone 3. This suggested a gradient of oxidative stress: even cells slightly removed from the front are experiencing and preparing for ROS attack, perhaps due to diffusion of hydrogen peroxide or ROS from the confrontation zone. These results aligned with previous reports that fungal antagonism induced oxidative stress and that fungi elevated ROS-scavenging enzymes in response [27]. Both *T.* sp. D and *A. niger* L14 were evidently investing in detoxifying harmful oxygen radicals generated during their confrontation.

Thereafter, this study found that *A. niger* L14 acidified the interface as a competitive tactic. The SMs previously isolated from *A. niger* L14 contained many organic acids; therefore, the pH of the medium during co-culture was measured (Figure 6C). A dramatic change in environmental pH was recorded as the interaction progressed, particularly on the *A. niger* L14 side of the confrontation. Initially, before contact, the pH of the medium in both halves of the plate was close to neutral (starting around pH 7.0 due to the MDA). After one day of direct hyphal contact, the pH at the immediate interface and just into the *A. niger* L14 zone had already dropped (to approximately pH 5.0). In contrast, the *T.* sp. D side and distant zones remained near pH 7.0. By four days of sustained interaction, the acidity near *A. niger* L14 became even more pronounced: the antagonistic zone and *A. niger* L14-proximal zone reached pH values between 3.0 and 4.0. In comparison, the *T.* sp. D side of the interface saw a milder pH reduction and the far *T.* sp. D zone stayed around pH 6.5. The far *A. niger* L14 side was also slightly acidic. These findings aligned with the idea that *A. niger* L14 used acidification as a weapon. By drastically lowering the pH, *A. niger* L14 could create an unfavorable environment for *T.* sp. D. In fact, the growth of *T.* sp. D was significantly inhibited in acidified zones, which may be due to reduced levels of its enzymes (such as Laccase or Cellulase) in low-pH environments, or to direct damage to the cells of *T.* sp. D. From another perspective, the acidification also influenced the chemistry at the interface. For instance, some of the pigment changes to orange-brown could be due to pH-dependent color changes in specific SMs or to enhanced activity of oxidative enzymes at acidic pH, producing quinones or pigments such as Fonsecin and Kojic acid. In short, controlling the microenvironment pH was a powerful strategy that *A. niger* L14 employed to dominate the interaction zone.

Ultimately, whether the conserved stress-activated protein kinase pathway (i.e., the HOG/MAPK signaling pathway homologous to p38) was activated during the fungal defense process was investigated (Figure 6D). ELISA results suggested activation of a p38/HOG MAPK stress response pathway, especially in *A. niger* L14. In *T.* sp. D, total p38 changed only slightly among treatments, whereas p-p38 increased from 14.39 U/L in monoculture to 80.33 U/L in direct co-culture and 38.86 U/L in the simulated co-culture group, with the p-p38/p38 ratio increasing from 1.34 to 7.27 and 3.40, respectively. A similar pattern was observed in *A. niger* L14, in which total p38 remained relatively stable, while p-p38 increased from 12.67 U/L in monoculture to 80.33 U/L in direct co-culture and 59.54 U/L in the simulated co-culture group, and the p-p38/p38 ratio increased from 1.18 to 7.27 and 5.26, respectively. In the simulated co-culture group, *A. niger* L14 also showed increased p-p38 levels and an elevated p-p38/p38 ratio, suggesting that *T.* sp. D-derived SMs contributed to activation of the MAPK signaling pathway in *A. niger* L14. The increased phospho-p38 suggested that *A. niger* L14 was sensing an extreme stress (cell wall stress from *T.* sp. D enzymes, oxidative stress, etc), and was mounting a global stress response. Activation of p38/HOG in fungi typically led to induction of osmoprotectant genes, cell wall repair, and SM regulation [16], all of which align with our observations of *A. niger* L14’s behavior. In *T.* sp. D, an increase in p38/HOG MAPK activation in co-culture was also detected, and the simulated culture group of *A. niger* L14-derived SMs also showed similar activation effects. In short, both fungi perceived the co-culture antagonism as severe stress, triggering conserved signaling cascades. This highlighted that beyond specific biochemical skirmishes, the fungi were also activating higher-level regulatory programs to adjust their physiology during competition.

### 3.6. An Integrated Model for Fungal-Fungal Confrontation in Co-Culture

**Figure 7:**
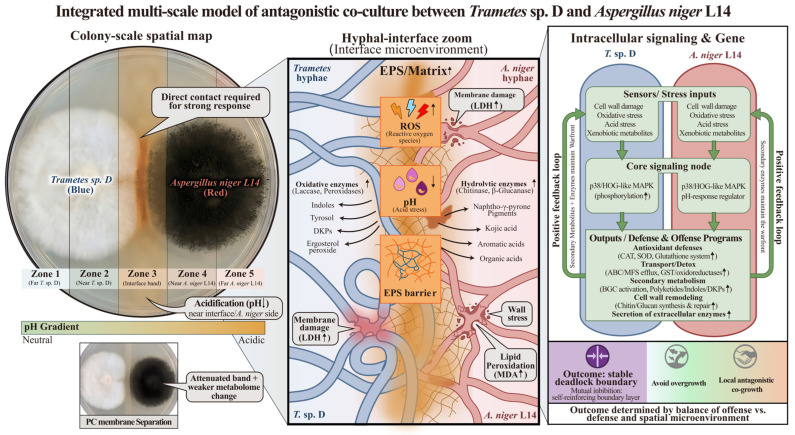
Integrated multi-scale mechanistic model of fungal-fungal confrontation leading to a stable barrage. The schematic summarized the confrontation from the colony scale to the interface microenvironment and intracellular regulation. Co-culture with direct hyphal contact was associated with a more pronounced boundary band. The interface featured matrix buildup, pH gradients, metabolite exchange, and enzyme attack/defense. p38/HOG-MAPK activation accompanied stress signaling during the deadlock.

The graphical mechanism model summarized four coupled layers of the confrontation between *T.* sp. D and *A. niger* L14 on MDA: (i) the direct contact-associated recognition step at the colony interface; (ii) reciprocal chemical antagonism, in which each fungus activated secondary metabolism and exported characteristic SMs into the antagonistic zone; (iii) localized enzymatic and physicochemical attacks that remodeled the microenvironment (cell-wall degradation, oxidative stress, extracellular matrix accumulation, acidification, etc.); (iv) conserved stress-response signaling outputs (ROS burden, membrane/wall injury, and antioxidant defenses, plus p38/HOG-MAPK activation) associated with enhanced detoxification, transport, and barrier formation, likely contributing to stabilization of the orange-brown barrage band (Figure 7).

In the integrated model, both colonies expanded until direct hyphal contact created an entangled battlefront where hyphae deform, and cellular injury began. This physical encounter was associated with marked metabolic reprogramming, converting the interface into a bidirectional chemical war zone: *T.* sp. D elevated tryptophan-derived indolic SMs, cyclic dipeptides, Ergosterol peroxide, etc., whereas *A. niger* L14 boosted polyketide-derived pigments, phenolic acids, organic acids, etc., output (such as Fonsecin, Ferulic acid). Each SM set was largely self-tolerated but suppressed the opponent, and cross-exposure further provoked counter-metabolite induction. In parallel, *T.* sp. D mainly deployed oxidative enzymes (including Laccases, Peroxidases, etc.) that delivered enhanced oxidizing power to attack the opponent, while *A. niger* L14 mainly deployed hydrolases (such as Chitinase, Glucanases) that weakened *T.* sp. D cell walls; both effects peaked at the antagonistic zone, coinciding with elevated lipid peroxidation and membrane leakage, and with increased extracellular polysaccharides as a protective barricade. *A. niger* L14 additionally acidified the nearby medium, creating a low-pH environment that penalized *T.* sp. D physiology and affected enzyme activity. These combined assaults were associated with activation of the p38/HOG-MAPK stress pathway in both fungi, along with increased antioxidant defense, efflux/transport, detoxification, and cell-envelope repair programs, likely contributing to a persistent standoff rather than overgrowth.

## 4. Discussion

Co-culture could elicit broad induction of otherwise cryptic SMs, underscoring the potent impact of interspecific interactions on fungal metabolism [28]. Numerous SMs were uniquely detected or greatly amplified in the antagonistic zone, consistent with reports that fungal co-culture could awaken silent BGCs. *T.* sp. D produced elevated levels of indole alkaloids and cyclodipeptides, while *A. niger* L14 overproduced polyketide pigments and organic acids, each inhibiting the counterpart’s growth but not the producer’s own mycelium. This reciprocity indicated a strategic chemical antagonism, in which each fungus deployed specific antibiotics to gain an advantage without harming itself. Comparable cases of interaction-specific metabolite production have been observed in other co-cultures: e.g., co-cultivation of marine fungi induced a rare 2-alkenyl-tetrahydropyran while simultaneously deactivating a competitor’s antifungal pyridoxatin via methylation [29]. Such findings suggested fungi not only activate new SMs under competition but could also chemically neutralize an opponent’s toxins. In this study, *A. niger* L14’s secretion of Kojic acid and other organic acids likely contributed to this phenomenon, as acidic pH could selectively impair rival enzymes and SMs [30]. Indeed, *A. niger* is a prolific acid producer in industry. In co-culture, this trait manifested as a competitive weapon: acidification of the medium by *A. niger* L14 resulted in a zone of inhibition against *T.* sp. D, presumably by denaturing *T.* sp. D-derived Laccases and other enzymes [17]. Our results showed that physical contact and chemical exchange between fungi could dramatically broaden their SM repertoires and shift the metabolic balance toward antagonism [31].

Importantly, direct hyphal contact substantially enhanced the antagonistic response between *T.* sp. D and *A. niger* L14. When the fungi were grown separated by PC, only attenuated metabolic and phenotypic changes occurred, whereas confrontation yielded a prominent barrage line with intense pigment and SMs accumulation. This direct contact-associated effect was consistent with observations in other systems, which showed that proximity and physical interaction were crucial triggers of secondary metabolism [32]. For instance, in co-cultures of ectomycorrhizal and mycoparasitic fungi, metabolomic profiling revealed hundreds of unique compounds emerging predominantly under direct contact conditions, with minimal response in diffusible-only setups [28,32]. Our microscopy experiments confirmed that *T.* sp. D hyphae physically intertwined with *A. niger* L14, and such intimate contact likely enabled exchange of signals or small diffusible molecules that activated cryptic biosynthetic genes. Contact-mediated signaling has been shown to induce fungal morphogenesis and the production of SMs. In the well-studied *Trichoderma*-*Laccaria* model, direct confrontation versus only volatile exposure led to vastly different outcomes: *Trichoderma*’s growth was more suppressed without contact, whereas *Laccaria* was strongly inhibited only with direct contact [32]. Similarly, *T.* sp. D showed more pronounced growth restriction under direct contact conditions with *A. niger* L14, suggesting that physical contact plays an important role in the observed antagonistic phenotype. Collectively, the present data support a major contribution of direct contact to the antagonistic phenotype, although more general colony-encounter effects cannot be entirely ruled out.

By integrating differentially expressed SMs of two fungi during co-culture with DEGs from the transcriptome, and by screening for correlation coefficients and analyzing gene pathways, the metabolite-gene networks were constructed to infer the biosynthetic pathways of SMs. For *T.* sp. D, 3-Indoleacetic acid likely arose from tryptophan via the Indole-3-pyruvate → Indole-3-acetaldehyde → 3-Indoleacetic acid route (e.g., *Aro8*, *Aro10*, ALDH), reinforced by an amidase-mediated Indole-3-acetamide → 3-Indoleacetic acid branch and a nitrilase-mediated putative Indole-3-acetonitrile → 3-Indoleacetic acid conversion (Figure S6). Indole-3-acetamide and Indole-3-carboxaldehyde were consistent with P450-driven oxidative steps feeding into these indole intermediates. Tyrosol was explained by a canonical Ehrlich route from Tyrosine (e.g., aminotransferase, decarboxylase, ADH, BDH) with reduced diversion to acid or Tyramine branches. Cyclo-(Pro-Phe) and Cyclo-(Leu-Pro) were linked to enhanced protein turnover plus suppressed dipeptide hydrolysis and proline catabolism, favoring diketopiperazine (DKP) accumulation. Ergosterol peroxide was rationalized as resulting from elevated sterol biosynthesis under oxidative stress, enabling ROS-mediated ergosterol peroxidation. For *A. niger* L14, co-culture enhanced carbohydrate depolymerization (e.g., xylanase, invertase), acidification, and oxidative stress, favoring non-enzymatic sugar dehydration to 5-hydroxymethyl-2-furfuraldehyde and its detoxification via ADH/AKR reduction and ALDH/P450 oxidation to 5-hydroxymethyl-2-furancarboxylic acid (Figure S7). Increased glucose flux supported Kojic acid formation through gluconate and oxidoreductases, while a γ-naphthopyrone precursor was O-methylated to Fonsecin. Phenylpropanoid-like conversions from phenylalanine produced Caffeic and Ferulic acids, followed by CoA activation, side-chain shortening, and aldehyde oxidation to Vanillic acid and O-methylation to Veratric acid. Elevated NAD^+^ turnover yielded Nicotinamide and Nicotinic acid via *nudC* and *PNC1*, and intensified nucleic-acid catabolism, leading to accumulation of Adenine and Xanthine.

The antagonism between *A. niger* L14 and *T.* sp. D put significant stress on each other, as evidenced by the upregulation of oxidative stress biomarkers in the interaction region. In both fungi, transcriptomic data indicated activation of stress-response pathways, notably the MAPK cascades analogous to the HOG/p38 pathway. The phosphorylation of p38/HOG MAPK was detected, suggesting that each fungus sensed interspecies contact as a high-stress condition, combined with osmotic, oxidative, and cell wall stress, and mobilized its stress signaling. Such MAPK activation is a common motif in fungal antagonisms [33]. In *T.* sp. D-*A. niger* L14 co-culture, MAPK activation may have contributed to the induction of protective enzymes (e.g., Catalases, Superoxide dismutases) to mitigate ROS, as well as enzymes that reinforced cell wall integrity (e.g., chitin and glucan synthases), hinting that each was both attacking the other’s cell wall and bolstering its own. This mirrored antagonistic interactions in other models, such as *Simplicillium* and *Heterobasidion*, in which the pathogen’s transcriptome showed enrichment for detoxification and membrane-stabilization genes under attack [34]. Overall, this study supported a model in which stress signaling pathways (MAPK cascades) and oxidative stress were associated with a broad stress response during fungal competition, arming the fungi with enhanced tolerance to toxins and physical damage.

An enzymatic arms race at the confrontation boundary characterized the antagonistic interaction. *T.* sp. D mainly released copious amounts of oxidative enzymes (such as Laccases, Peroxidases) into the agar, while *A. niger* L14 mainly secreted high levels of hydrolytic enzymes (such as Chitinases, β-glucanases) along with organic acids. Spatial enzyme assays showed a peak of several enzyme activities precisely at the interface, where *A. niger* L14’s hyphae contacted *T.* sp. D [35]. These enzymes may promote the degradation of fungal cell walls, while *T.* sp. D’s oxidases can prevent damage to its own hyphae and detoxify phenolic acid toxins secreted by *A. niger* L14 [27]. The interface zone in our co-culture exhibited distinctive orange-brown pigmentation, a marker of fungal stress and cell wall injury. The production of organic acids by *A. niger* L14 further acidified the local medium, potentially inhibiting *T.* sp. D enzyme activities and thus protecting *A. niger* L14 from oxidative damage. Notably, transcriptomic analysis of both fungi in co-culture revealed enhanced expression of genes associated with cell wall integrity and drug resistance, including ABC transporters, efflux pumps, and cell wall synthases. These changes echoed those seen in other antagonistic systems: e.g., confronted fungi often upregulated transporter genes to pump out the opponent’s toxins and increased chitin synthase expression to repair cell wall damage [34]. The integration of metabolomic and transcriptomic data showed that *T.* sp. D and *A. niger* L14 both sensed the attack on their cell envelope, accompanied by activation of signaling pathways including MAPKs, increased SM biosynthesis, secretion of antagonistic enzymes, and activation of defense genes for detoxification and wall reinforcement. This co-culture system underscored the core mechanisms: physical contact initiation, induction of SMs and enzyme production, oxidative stress burst, and stress signaling feedback. *T.* sp. D relied on oxidative reactions and antifungal SMs, whereas *A. niger* L14 emphasized acidification and hydrolytic enzyme attack. The outcome was a deadlock: neither fungus eradicated the other, instead forming a lasting antagonistic zone where both maintained viability behind their battlefront. The co-culture of *T.* sp. D and *A. niger* L14 demonstrated how combining two fungi could induce a wealth of bioactive SMs and enzymes that might not be available in large quantities in monocultures. Harnessing such induced outputs could be useful, for instance, by prompting fungi to produce new antibiotics or enzymes through controlled confrontation [36]. In conclusion, our multi-omics analysis of this fungal duel provided a detailed mechanistic insight: physical contact was associated with a complex chain of stress signaling that integrated with secondary metabolism, leading each fungus to deploy specialized SMs and enzymes, modulate environmental conditions, and activate defense networks. These findings not only elucidated how natural interactions between *Trametes* spp. and *Aspergillus* spp. were governed, but also contributed broadly to the emerging understanding of fungal-fungal ecology, in which competition can drive metabolic innovation and chemical warfare between species can uncover compounds and biochemical pathways with potential biotechnological value.

Despite these strengths, several limitations of the present study should be acknowledged. The target networks and molecular docking analyses provide hypothesis-generating evidence for metabolite-protein interactions; however, they do not constitute direct proof of binding or in vivo functional inhibition and therefore require further biochemical and genetic validation. The proposed biosynthetic routes are inferred from integrated metabolomic-transcriptomic correlations rather than direct pathway reconstruction. In addition, because the mechanistic conclusions were derived from a single fungal pair under a defined plate-based co-culture system on MDA, the broader applicability of these interaction patterns to other strains, substrates, and ecological settings remains to be determined.

## 5. Conclusions

The antagonistic zone observed between *T.* sp. D and *A. niger* L14 was a visible outcome of intense chemical and enzymatic warfare between these fungi. Physical contact was associated with activation of otherwise silent SM gene clusters in both species, yielding antifungal compounds not produced in monocultures. Simultaneously, *T.* sp. D secreted oxidative enzymes (e.g., Laccases) to degrade *A. niger* L14 hyphae, while *A. niger* L14 released hydrolytic enzymes and organic acids to acidify the interface and impede *T.* sp. D enzyme activity. These interactions were associated with activation of a p38/HOG-MAPK signaling cascade, together with upregulation of defense-related genes and protective cell wall remodeling. Ultimately, these antagonistic strategies resulted in a stalemate, as evidenced by the antagonistic zone, which illustrated how co-culture competition delineated microbial community boundaries and could induce the production of bioactive SMs, with implications for natural product discovery.

## Data Availability

The original contributions presented in this study are included in the article. Further inquiries can be directed to the corresponding author.

## Supplementary Materials

The following supporting information can be downloaded at: https://www.mdpi.com/article/10.3390/jof12050327/s1, Figure S1: Time-resolved macroscopic phenotypes of confrontation and the effect of physical separation; Figure S2: Induced or augmented SMs in co-culture and their selective antagonistic effects; Figure S3: Plate assays using mixtures of all identified SMs demonstrate selective antagonism without self-inhibition; Figure S4: Interaction network linking the 12 *A. niger* L14-derived SMs to predicted gene targets and co-culture-upregulated annotated-KEGG genes in *T.* sp. D (ultra-high definition image); Figure S5: Interaction network linking the 7 *T.* sp. D-derived SMs to predicted gene targets and co-culture-upregulated annotated-KEGG genes in *A. niger* L14 (ultra-high definition image); Figure S6: Metabolite-gene networks and inferred biosynthetic routes for *T.* sp. D co-culture metabolites; Figure S7: Metabolite-gene networks and inferred biosynthetic routes for *A. niger* L14 co-culture metabolites; Figure S8: Original uncropped SEM image (500×) of *T.* sp. D grown in monoculture; Figure S9: Original uncropped SEM image (500×) of *A. niger* L14 grown in monoculture; Figure S10: Original uncropped SEM image (500×) of co-culture; Figure S11: Original uncropped SEM image (1000×) of *T.* sp. D grown in monoculture; Figure S12: Original uncropped SEM image (1000×) of *A. niger* L14 grown in monoculture; Figure S13: Original uncropped SEM image (1000×) of co-culture; Dataset S1: Supplementary Figure Legends; Dataset S2: 1D NMR spectra and HRESIMS spectra of 34 compounds; Table S1: Gene and protein compilation; Table S2: Reciprocal modulation of major SMs upon exogenous metabolite challenge; Table S3: Molecular docking results of seven *T.* sp. D-derived SMs with *A. niger*; Table S4: Molecular docking results of twelve *A. niger* L14-derived SMs with *T. versicolor*.

## Abbreviations

The following abbreviations are used in this manuscript:

SM	Secondary metabolite
MAPK	Mitogen-activated protein kinase
BGC	Biosynthetic gene cluster
MDA	Wheat bran leachate agar medium
PC	Polycarbonate membrane
LC-MS	Liquid chromatography-mass spectrometry
UHPLC-QTOF-MS/MS	Ultra-high-performance liquid chromatography-quadrupole time-of-flight tandem mass spectrometry
SEM	Scanning electron microscopy
MPLC	Medium-pressure liquid chromatography
HPLC	High-performance liquid chromatography
RNA-seq	RNA sequencing
FPKM	Fragments per kilobase of exon model per million mapped fragments
GO	Gene ontology
KEGG	Kyoto encyclopedia of genes and genomes
DL	Co-culture group
DEGs	Differentially expressed genes
LDH	Lactate dehydrogenase
SOD	Superoxide dismutase
CAT	Catalase
GSH-Px	Glutathione peroxidase
HOG	High osmolarity glycerol
ELISA	Enzyme-linked immunosorbent assay
ROS	Reactive oxygen species
DKP	Diketopiperazine
