# Supplementary material for: From Contact to Stalemate: MAPK-Associated Chemical and Enzymatic Defenses Shape a Stable Barrage in the Co-Culture of Trametes sp. D and Aspergillus niger L14
Source: J Fungi (Basel). 2026 Apr 30;12(5):327. doi: 10.3390/jof12050327 (PMC13208484; doi:10.3390/jof12050327)
Supplement: Supplementary file 1 [file jof-12-00327-s001.zip › Dataset S1_Supplementary Figure Legends.pdf]

**Figure S1.** Time-resolved macroscopic phenotypes of confrontation and the effect of physical separation. (A) Photographs of direct co-culture on MDA showing the development of the confrontation band over time (e.g., early golden-yellow band progressing to an orange-brown band; images from front/back views). (B) Photographs of PC membrane-separated co-culture on MDA (front/back views), showing that physical separation attenuates band formation and weakens the confrontation phenotype relative to direct contact.

**Figure S2.** Induced or augmented SMs in co-culture and their selective antagonistic effects. (A) Dose-dependent growth inhibition assays for *T.* sp. D-derived SMs tested against both strains. (B) Dose-dependent growth inhibition assays for *A. niger* L14-derived SMs tested against both strains. Data were presented as mean ± SD (n = 3). Statistical differences among groups were evaluated by one-way ANOVA. Asterisks indicated significant differences compared with the *T.* sp. D monoculture, and hash symbols indicated significant differences compared with the *A. niger* L14 monoculture. *P < 0.05, **P < 0.01, ***P < 0.001 vs. D; $^{\#}$P < 0.05, $^{\#\#}$P < 0.01, $^{\#\#\#}$P < 0.001 vs. L14; ns, not significant.

**Figure S3.** Plate assays using mixtures of all identified SMs demonstrate selective antagonism without self-inhibition. (A) Structures of the 7 *T.* sp. D-derived SMs and representative growth phenotypes of *T.* sp. D and *A. niger* L14 on MDA with/without the mixed SMs, illustrating selective inhibition of the competitor with minimal producer self-inhibition. (B) Structures of the 12 *A. niger* L14-derived SMs and representative growth phenotypes of *T.* sp. D and *A. niger* L14 on MDA with/without the mixed SMs, illustrating selective inhibition of *T.* sp. D with minimal inhibition of *A. niger* L14.

**Figure S4.** Interaction network linking the 12 *A. niger* L14-derived SMs to predicted gene targets and co-culture-upregulated annotated-KEGG genes in *T.* sp. D (ultra-high definition image).

**Figure S5.** Interaction network linking the 7 *T.* sp. D-derived SMs to predicted gene targets and co-culture-upregulated annotated-KEGG genes in *A. niger* L14 (ultra-high definition image).

**Figure S6.** Metabolite-gene networks and inferred biosynthetic routes for *T.* sp. D co-culture metabolites. (A) Metabolite-gene correlation networks for the major *T.* sp. D-derived SMs induced/enhanced in co-culture. (B) Proposed biosynthetic and regulatory schemes for the *T.* sp. D SM set, inferred from integrated metabolomics-transcriptomics and KEGG/pathway annotation, including tryptophan-derived indole routes, aromatic alcohol formation (Tyrosol), cyclic dipeptide association, sterol-derived transformation (Ergosterol peroxide), and linked stress/defense-related gene modules.

**Figure S7.** Metabolite-gene networks and inferred biosynthetic routes for *A. niger* L14 co-culture metabolites. (A) Metabolite-gene correlation networks for the major *A. niger* L14-derived SMs induced/enhanced in co-culture. (B) Proposed biosynthetic and regulatory schemes for the *A. niger* L14 SM set inferred from integrated metabolomics-transcriptomics and KEGG annotation, covering carbohydrate-derived Kojic acid formation, polyketide/pigment-associated routes (e.g., Fonsecin-related), aromatic acid metabolism, purine/nicotinamide-related metabolism, and associated stress/defense gene programs.

**Figure S8.** Original uncropped SEM image (500 ×) of *T.* sp. D grown in monoculture.

**Figure S9.** Original uncropped SEM image (500 ×) of *A. niger* L14 grown in monoculture.

**Figure S10.** Original uncropped SEM image (500 ×) of co-culture.

**Figure S11.** Original uncropped SEM image (1000 ×) of *T.* sp. D grown in monoculture.

**Figure S12.** Original uncropped SEM image (1000 ×) of *A. niger* L14 grown in monoculture.

**Figure S13.** Original uncropped SEM image (1000 ×) of co-culture.
