# Supplementary material for: From Contact to Stalemate: MAPK-Associated Chemical and Enzymatic Defenses Shape a Stable Barrage in the Co-Culture of Trametes sp. D and Aspergillus niger L14
Source: J Fungi (Basel). 2026 Apr 30;12(5):327. doi: 10.3390/jof12050327 (PMC13208484; doi:10.3390/jof12050327)
Supplement: Supplementary file 1 [file jof-12-00327-s001.zip › Dataset S2_1D NMR spectra and HRESIMS spectra of 34 compounds.pdf]

Structural identification of 34 compounds

（1）Compound **1**：Xanthine

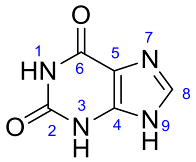

Structure of compound 1

^{1}H NMR (600 MHz, DMSO-d$_6$) data of compound 1

Position	δ_H
1-NH, 3-NH, 9-NH	13.27 (1H, br. s), 11.47 (1H, br. s), 10.78 (1H, br. s)
8	7.90 (1H, s)

（2）Compound **2**：Adenine

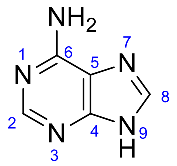

Structure of compound 2

^{1}H NMR (600 MHz, DMSO-*d*$_6$) data of compound 2

Position	δ_H
2	8.09 (1H, s)
6-NH2	7.04 (2H, s)
8	8.07 (1H, s)
9-NH	12.73 (1H, br. s)

（3）Compound **3**：Nicotinamide

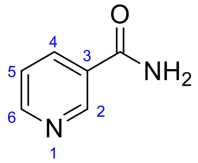

Structure of compound 3

^{1}H (600 MHz, DMSO-*d*$_6$) and ^{13}C (150 MHz, DMSO-*d*$_6$) NMR data of compound 3

Position	δ_H	δ_C
2	9.00 (1H, s)	148.6
3		129.6
4	8.18 (1H, br. d, *J* = 7.8 Hz)	135.1
5	7.47 (1H, dd, *J* = 7.8, 4.2 Hz)	123.3
6	8.68 (1H, d, *J* = 4.2 Hz)	151.8
$CONH_2$	8.12, 7.56 (each 1H, br. s)	166.4

（4）Compound **4**：Nicotinic acid

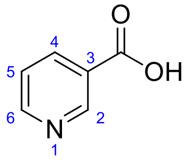

Structure of compound 4

^{1}H (600 MHz, DMSO-d_6) and ^{13}C (150 MHz, DMSO-d_6) NMR data of compound 4

Position	δ_H	δ_C
2	9.05 (1H, s)	150.2
3		126.5
4	8.24 (1H, d, J = 7.8 Hz)	136.9
5	7.52 (1H, dd, J = 7.8, 4.2 Hz)	123.8
6	8.76 (1H, d, J = 4.2 Hz)	153.3
COOH	13.39 (1H, br. s)	166.2

（5）Compound **5**：Kojic acid

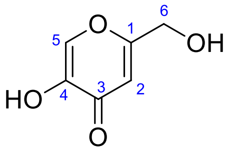

Structure of compound 5

^{1}H (600 MHz, DMSO-d_6) and ^{13}C (150 MHz, DMSO-d_6) NMR data of compound 5

Position	δ_H	δ_C
1		168.0
2	6.31 (1H, s)	109.8
3		173.8
4		145.7
4-OH	9.01 (1H, br. s)	
5	8.00 (1H, s)	139.2
6	4.26 (2H, br. s)	59.4
6-OH	5.63 (1H, br. s)	

（6）Compound **6**：5-Hydroxymethyl-2-furancarboxylic acid

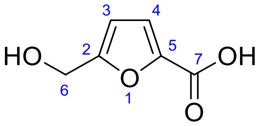

Structure of compound 6

^{1}H NMR (600 MHz, DMSO-d_6) data of compound 6

Position	δ_H
3	6.43 (1H, d, J = 3.4 Hz)
4	7.12 (1H, d, J = 3.4 Hz)

6	4.42 (2H, s)
6-OH	5.42 (1H, br. s)
7-COOH	12.92 (1H, s)

（7）Compound **7**：5-Hydroxymethyl-2-furfuraldehyde

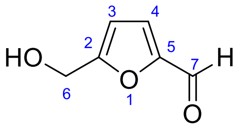

Structure of compound 7

^{1}H NMR (600 MHz, DMSO-d_6) data of compound 7

Position	δ_H
3	6.58 (1H, d, J = 3.4 Hz)
4	7.46 (1H, d, J = 3.4 Hz)
6	4.48 (2H, s)
6-OH	5.53 (1H, br. s)
7	9.52 (1H, s)

（8）Compound **8**：Vanillic acid

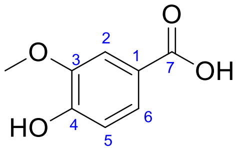

Structure of compound 8

^{1}H NMR (600 MHz, DMSO-d_6) data of compound 8

Position	δ_H
2	7.41 (1H, d, J = 1.7 Hz)
5	6.82 (1H, d, J = 8.2 Hz)
6	7.42 (1H, dd, J = 8.2, 1.7 Hz)
3-OMe	3.78 (3H, s)
4-OH	9.79 (1H, br. s)
7-COOH	12.44 (1H, br. s)

（9）Compound **9**：Caffeic acid

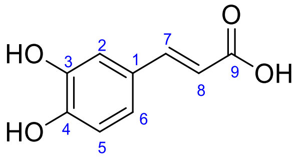

Structure of compound 9

^{1}H NMR (600 MHz, DMSO-d_6) data of compound 9

Position	δ_H

2	7.00 (1H, d, J = 1.3 Hz)
3-OH	9.09 (1H, br. s)
4-OH	9.48 (1H, br. s)
5	6.73 (1H, d, J = 7.8 Hz)
6	6.94 (1H, dd, J = 7.8, 1.3 Hz)
7	7.39 (1H, d, J = 16.0 Hz)
8	6.14 (1H, d, J = 16.0 Hz)
9-COOH	12.07 (1H, br. s)

（10）Compound **10**：Ferulic acid

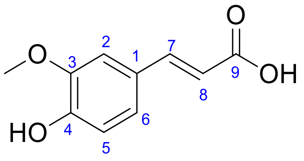

Structure of compound 10

^{1}H NMR (600 MHz, DMSO-d_6) data of compound 10

Position	δ_H
2	7.25 (1H, br. s)
5	6.76 (1H, d, J = 8.2 Hz)
6	7.05 (1H, br. d, J = 8.2 Hz)
7	7.46 (1H, d, J = 15.8 Hz)
8	6.34 (1H, d, J = 15.8 Hz)
3-OMe	3.79 (3H, s)
4-OH	9.50 (1H, br. s)
9-COOH	12.08 (1H, br. s)

（11）Compound **11**：Tyrosol

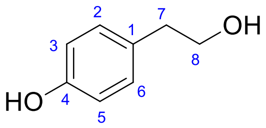

Structure of compound 11

^{1}H NMR (600 MHz, DMSO-d_6) data of compound 11

Position	δ_H
2,6	6.96 (2H, d, J = 8.0 Hz)
3,5	6.93 (2H, d, J = 8.0 Hz)
4-OH	9.07 (1H, s)
7	2.57 (2H, t, J = 7.2 Hz)
8	3.80 (2H, td, J = 7.2, 5.0 Hz)
8-OH	4.52 (1H, t, J = 5.0 Hz)

（12）Compound **12**：1H-Indole-3-acetamide

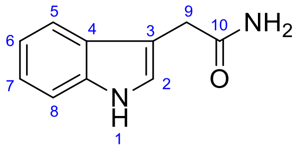

Structure of compound 12

^{1}H NMR (600 MHz, DMSO-d_6) data of compound 12

Position	δ_H
1-NH	10.82 (1H, br. s)
2	7.16 (1H, s)
5	7.52 (1H, d, J = 7.8 Hz)
6	6.95 (1H, dd, J = 8.1, 7.8 Hz)
7	7.05 (1H, t, J = 8.1 Hz)
8	7.35 (1H, d, J = 8.1 Hz)
9	3.44 (2H, s)
10-NH_2	7.25, 6.79 (each 1H, br. s)

（13）Compound **13**：3-Indoleacetic acid

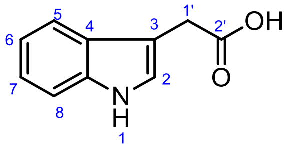

Structure of compound 13

^{1}H NMR (600 MHz, DMSO-d_6) data of compound 13

Position	δ_H
1-NH	10.87 (1H, s)
2	7.20 (1H, s)
5	7.47 (1H, d, J = 8.0 Hz)
6	6.96 (1H, t, J = 8.0 Hz)
7	7.05 (1H, t, J = 8.0 Hz)
8	7.33 (1H, d, J = 8.0 Hz)
1'	3.61 (2H, s)
2'-COOH	12.10 (1H, br. s)

（14）Compound **14**：Veratric acid

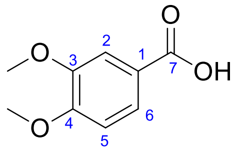

Structure of compound 14

^{1}H NMR (600 MHz, DMSO-d_6) data of compound 14

Position	δ_H

2	7.42 (1H, d, J = 1.0 Hz)
5	7.02 (1H, d, J = 8.5 Hz)
6	7.54 (1H, dd, J = 8.5, 1.0 Hz)
3-OMe, 4-OMe	3.80 (3H, s), 3.78 (3H, s)
7-COOH	12.62 (1H, br. s)

（15）Compound **15**：1H-Indole-3-carboxaldehyde

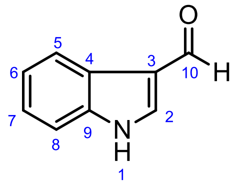

Structure of compound 15

^{1}H (600 MHz, DMSO-d_6) NMR data of compound 15

Position	δ_H
1-NH	12.09 (1H, br. s)
2	8.26 (1H, s)
5	8.08 (1H, d, J = 7.2 Hz)
6	7.20 (1H, dd, J = 8.4, 7.2 Hz)
7	7.24 (1H, t, J = 8.4 Hz)
8	7.49 (1H, d, J = 8.4 Hz)
10	9.92 (1H, s)

（16）Compound **16**：Ergosterol peroxide

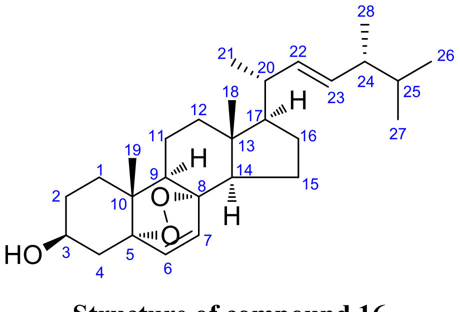

Structure of compound 16

^{1}H (600 MHz, DMSO-d_6) and ^{13}C (150 MHz, DMSO-d_6) NMR data of compound 16

Position	δ_H	δ_C
1		34.45
2		29.90
3	3.54 (1H, m)	64.59
4		36.44
5		81.40
6	6.41 (1H, d, J = 8.5 Hz)	135.53
7	6.20 (1H, d, J = 8.5 Hz)	130.07
8		78.36
9		50.84

10		36.87
11		22.78
12		38.67
13		43.98
14		51.18
15		20.20
16		28.22
17		55.39
18	0.75 (3H, s)	12.54
19	0.78 (3H, s)	17.85
20		40.04
21	0.95 (3H, d, *J* = 6.6 Hz)	20.69
22	5.14 (1H, dd, *J* = 15.4, 8.4 Hz)	135.17
23	5.21 (1H, dd, *J* = 15.4, 7.8 Hz)	131.51
24		41.96
25		32.43
26	0.80 (3H, d, *J* = 6.6 Hz)	19.43
27	0.79 (3H, d, *J* = 6.6 Hz)	19.73
28	0.86 (3H, d, *J* = 6.6 Hz)	17.27
OH	4.57 (1H, br. s)	

（17）Compound **17**：Fonsecin

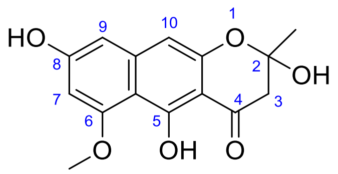

Structure of compound 17

^{1}H NMR (600 MHz, DMSO-d_6) data of compound 17

Position	δ_H
2-CH_3	1.57 (3H, s)
3	3.13 (1H, d, *J* = 17.4 Hz)<br>2.69 (1H, d, *J* = 17.4 Hz)
5-OH	14.17 (1H, s)
6-OCH_3	3.81 (3H, s)
7	6.29 (1H, br. s)
9	6.44 (1H, br. s)
10	6.39 (1H, s)

（18）Compound **18**：Cyclo-(Leu-Pro)

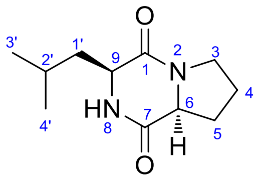

Structure of compound 18

^{1}H (600 MHz, DMSO-d_6) and ^{13}C (150 MHz, DMSO-d_6) NMR data of compound 18

Position	δ_H	δ_C
1		166.5
3	3.35 (1H, m), 3.29 (1H, m)	44.8
4	1.71-1.93 (2H, m)	21.9
5	2.10 (1H, m), 1.71-1.93 (1H, m)	27.4
6	4.16 (1H, t, J = 8.0 Hz)	58.4
7		170.3
8-NH	7.97 (1H, br. s)	
9	3.98 (1H, t, J = 6.2 Hz)	52.6
1'	1.71-1.93 (2H, m)	37.8
2'	1.33 (1H, m)	24.0
3'	0.85 (3H, d, J = 6.4 Hz)	22.4
4'	0.84 (3H, d, J = 6.4 Hz)	22.8

（19）Compound **19**：Cyclo-(Pro-Phe)

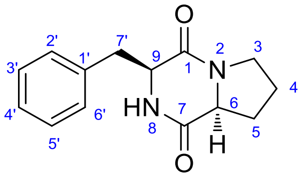

Structure of compound 19

^{1}H (600 MHz, DMSO-d_6) and ^{13}C (150 MHz, DMSO-d_6) NMR data of compound 19

Position	δ_H	δ_C
1		165.5
3	3.37 (1H, m), 3.25 (1H, m)	45.0
4	1.70 (1H, m), 1.40 (1H, m)	22.3
5	1.98 (1H, m), 1.70 (1H, m)	28.2
6	4.05 (1H, t, J = 8.2 Hz)	58.9
7		169.5
8-NH	7.94 (1H, br. s)	
9	4.33 (1H, t, J = 5.3 Hz)	56.2
1'		137.7
2', 6'	7.15-7.27 (2H, m)	130.2
3', 5'	7.15-7.27 (2H, m)	128.4
4'	7.15-7.27 (1H, m)	126.8
7'	3.05 (1H, dd, J = 14.4, 5.3 Hz)<br>3.00 (1H, dd, J = 14.4, 5.3 Hz)	35.8

（20）Compound **20**：Cyclo-(Gly-Pro)

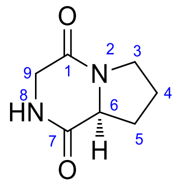

Structure of compound 20

^{1}H (600 MHz, DMSO-d_6) and ^{13}C (150 MHz, DMSO-d_6) NMR data of compound 20

Position	δ_H	δ_C
1		163.8
3	3.39 (1H, m), 3.32 (1H, m)	45.9
4	1.86-1.72 (2H, m)	22.0
5	2.11 (1H, m)<br>1.86-1.72 (1H, m)	27.8
6	4.10 (1H, t, *J* = 7.5 Hz)	58.0
7		169.2
8-NH	8.03 (1H, br. s)	
9	3.97 (1H, d, *J* = 16.7 Hz)<br>3.48 (1H, dd, *J* = 16.7, 4.5 Hz)	44.6

（21）Compound **21**：4-Hydroxybenzoic acid

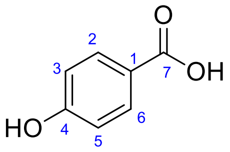

Structure of compound 21

^{1}H NMR (600 MHz, DMSO-d_6) data of compound 21

Position	δ_H
2, 6	7.76 (2H, d, *J* = 8.3 Hz)
3, 5	6.80 (2H, d, *J* = 8.3 Hz)
7-COOH	12.37 (1H, br. s)
OH	10.17 (1H, br. s)

（22）Compound **22**：4-Hydroxybenzaldehyde

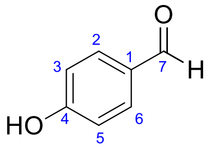

Structure of compound 22

^{1}H NMR (600 MHz, DMSO-d_6) data of compound 22

Position	δ_H
2, 6	7.74 (2H, d, *J* = 8.3 Hz)
3, 5	6.91 (2H, d, *J* = 8.3 Hz)
7-CHO	9.77 (1H, s)

4-OH	10.56 (1H, br. s)

（23）Compound **23**：4-Methoxybenzeneacetic acid

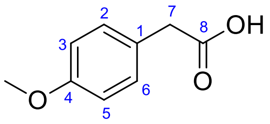

Structure of compound 23

^{1}H NMR (600 MHz, DMSO-d_6) data of compound 23

Position	δ_H
2, 6	7.14 (2H, d, J = 8.0 Hz)
3, 5	6.84 (2H, d, J = 8.0 Hz)
7	3.46 (2H, s)
4-OMe	3.71 (3H, s)
8-COOH	12.19 (1H, br. s)

（24）Compound **24**：Funatrol D

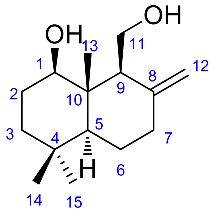

Structure of compound 24

^{1}H (600 MHz, DMSO-d_6) and ^{13}C (150 MHz, DMSO-d_6) NMR data of compound 24

Position	δ_H	δ_C
1	3.36 (1H, dd, J = 10.3, 5.4 Hz)	75.8
2		27.8
3		39.5
4		32.9
5	1.05 (1H, dd, J = 12.7, 2.8)	53.0
6		23.9
7	2.27 (1H, J = ddd, 12.2, 4.3, 2.7 Hz)<br>1.95 (1H, td, J = 12.2, 5.4 Hz)	37.5
8		149.0
9	1.85 (1H, dd, J = 8.8, 3.2 Hz)	57.2
10		44.7
11	3.68 (1H, dd, J =11.5, 8.8 Hz)<br>3.55 (1H, dd, J = 11.5, 3.2 Hz)	59.8
12	4.69, 4.30 (each 1H, br. s)	106.7
13	0.66 (3H, s)	9.4
14	0.74 (3H, s)	21.3
15	0.81 (3H, s)	32.8
1-OH and 11-OH	5.50, 5.41 (each 1H, br. s)	

（25）Compound **25**：1-Monolinolein

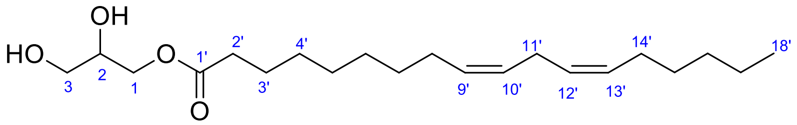

Structure of compound 25

^{1}H NMR (600 MHz, DMSO-d_6) data of compound 25

Position	δ_H
2'	2.26 (2H, t, J = 7.8 Hz)
3'	1.49 (2H, m)
4'-7'	1.21-1.34 (8H, m)
8'	2.00 (2H, q, J = 6.6 Hz)
9', 10', 12', 13'	5.26-5.36 (4H, m)
11'	2.71 (2H, t, J = 6.6 Hz)
14'	2.00 (2H, q, J = 6.6 Hz)
15'-17'	1.21-1.34 (6H, m)
18'	0.84 (3H, t, J = 7.2 Hz)
1	4.01 (1H, dd, J = 10.8, 4.2 Hz)<br>3.88 (1H, dd, J = 10.8, 6.6 Hz)
2	3.61 (1H, m)
3	3.27-3.35 (2H, m)
2-OH and 3-OH	4.82, 4.59 (each 1H, br. s)

（26）Compound **26**：Palmitic acid

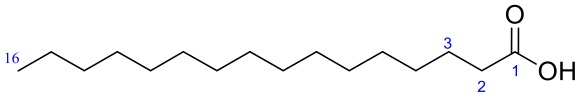

Structure of compound 26

^{1}H (600 MHz, DMSO-d_6) NMR data of compound 26

Position	δ_H
1-COOH	11.92 (1H, br. s)
2	2.15 (2H, t, J = 7.2 Hz)
3	1.46 (2H, m)
4-15	1.21 (24H, m)
16	0.83 (3H, t, J = 6.6 Hz)

（27）Compound **27**：Linoleic acid

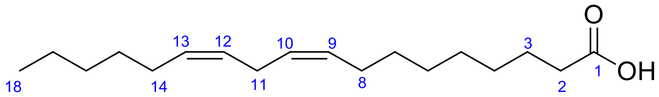

Structure of compound 27

^{1}H NMR (600 MHz, DMSO-d_6) data of compound 27

Position	δ_H
1-COOH	11.92 (1H, br. s)
2	2.16 (2H, t, J = 7.8 Hz)
3	1.46 (2H, m)
4-7	1.19-1.33 (8H, m)
8	2.00 (2H, q, J = 6.6 Hz)
9, 10, 12, 13	5.25-5.35 (4H, m)
11	2.71 (2H, t, J = 6.6 Hz)
14	2.00 (2H, q, J = 6.6 Hz)
15-17	1.19-1.33 (6H, m)
18	0.84 (3H, t, J = 7.2 Hz)

（28）Compound **28**：Stearic acid

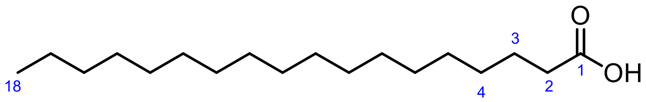

Structure of compound 28

^{1}H NMR (600 MHz, CD_3OD) data of compound 28

Position	δ_H
2	2.26 (2H, t, J = 7.3 Hz)
3	1.59 (2H, m)
4-17	1.29 (28H, m)
18	0.89 (3H, t, J = 6.8 Hz)

（29）Compound **29**：Carbonarone A

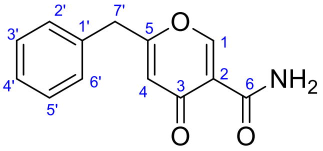

Structure of compound 29

^{1}H (600 MHz, DMSO-d_6) and ^{13}C (150 MHz, DMSO-d_6) NMR data of compound 29

Position	δ_H	δ_C
1	8.77 (1H, s)	162.1
2		119.3
3		177.6
4	6.41 (1H, s)	115.4
5		163.0
6		168.8
6-NH_2	8.53 ,7.73 (each 1H, br. s)	
1'		135.2
2', 6'	7.25-7.36 (2H, m)	129.0 (2C)
3',5'	7.25-7.36 (2H, m)	128.7 (2C)
4'	7.25-7.36 (1H, m)	127.2
7'	3.97 (2H, s)	38.2

（30）Compound **30**：Dimethyl phthalate

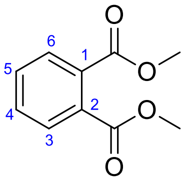

Structure of compound 30

^{1}H NMR (600 MHz, DMSO-d_6) data of compound 30

Position	δ_H
3,6	7.73 (2H, m)
4,5	7.67 (2H, m)
$COOCH_3$	3.80 (6H, s)

（31）Compound **31**：Rubrofusarin B

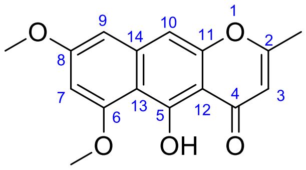

Structure of compound 31

^{1}H NMR (600 MHz, DMSO-d_6) data of compound 31

Position	δ_H
2-CH_3	2.37 (3H, s)
3	6.17 (1H, s)
5-OH	14.81 (1H, s)
7	6.45 (1H, d, J = 2.4 Hz)
9	6.84 (1H, d, J = 2.4 Hz)
10	7.16 (1H, s)
6-OCH_3 and 8-OCH_3	3.87, 3.86 (each 3H, s)

（32）Compound **32**：Asperxanthon

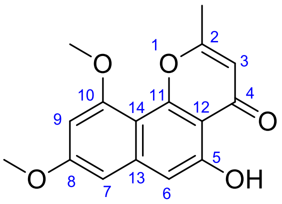

Structure of compound 32

^{1}H (600 MHz, DMSO-d_6) and ^{13}C (150 MHz, $CDCl_3$) NMR data of compound 32

Position	δ_H	δ_C
2		166.6
2-CH_3	2.48 (3H, s)	20.5
3	6.45 (1H, s)	110.2

4		182.8
5		156.6
5-OH	12.89 (1H, s)	
6	6.89 (1H, s)	105.8
7	6.81 (1H, br. s)	97.9
8		161.4
9	6.51 (1H, br. s)	97.0
10		159.0
11		155.8
12		104.9
13		141.2
14		108.8
8, 10-OCH_3	3.92, 3.86 (each 3H, s)	55.9, 55.4

（33）Compound **33**：Dibutyl phthalate

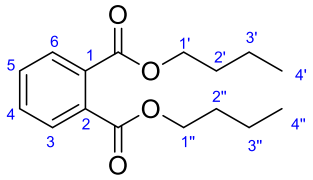

Structure of compound 33

^{1}H NMR (600 MHz, DMSO-*d*$_6$) data of compound 33

Position	δ_H
3,6	7.70 (2H, m)
4,5	7.64 (2H, m)
1,1"	4.20 (4H, d, *J* = 6.6 Hz)
2,2"	1.62 (4H, m)
3,3"	1.35 (4H, m)
4',4"	0.89 (6H, t, *J* = 6.4 Hz)

（34）Compound **34**：Palmitoleic acid

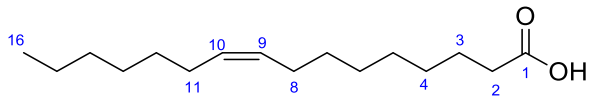

Structure of compound 34

^{1}H NMR (600 MHz, DMSO-*d*$_6$) data of compound 34

Position	δ_H
1-COOH	11.93 (1H, br. s)
2	2.15 (2H, t, *J* = 7.2 Hz)
3	1.46 (2H, m)
4-7	1.14-1.32 (8H, m)
8,11	1.96 (4H, q, *J* = 6.4 Hz)
9,10	5.26-5.34 (2H, m)
12-15	1.14-1.32 (8H, m)
16	0.83 (3H, t, *J* = 7.0 Hz)

1D NMR spectra and HRESIMS spectra of 34 compounds

Compound **1**:

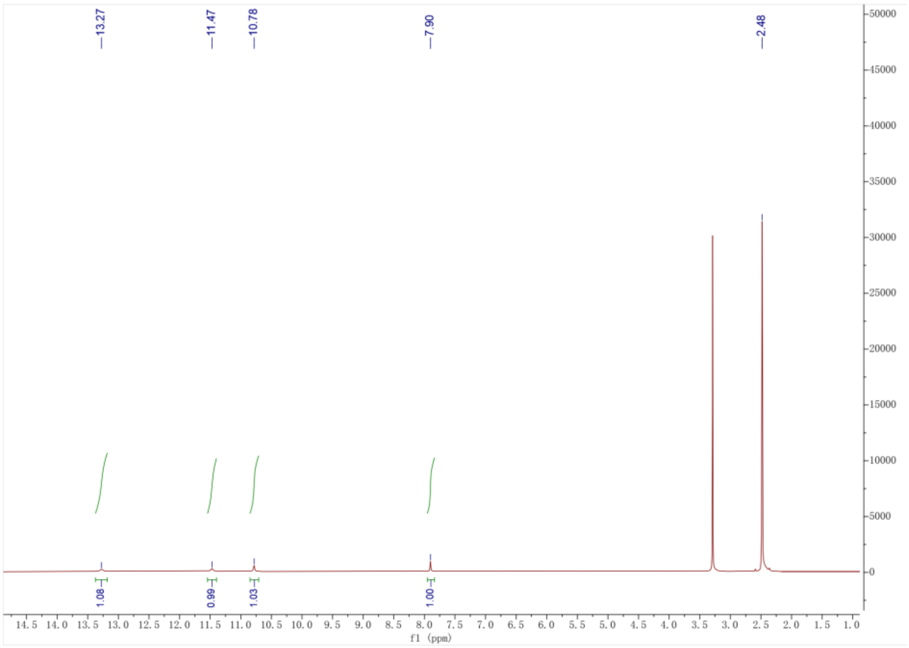

¹H-NMR spectrum（DMSO-*d*6, 600 MHz）

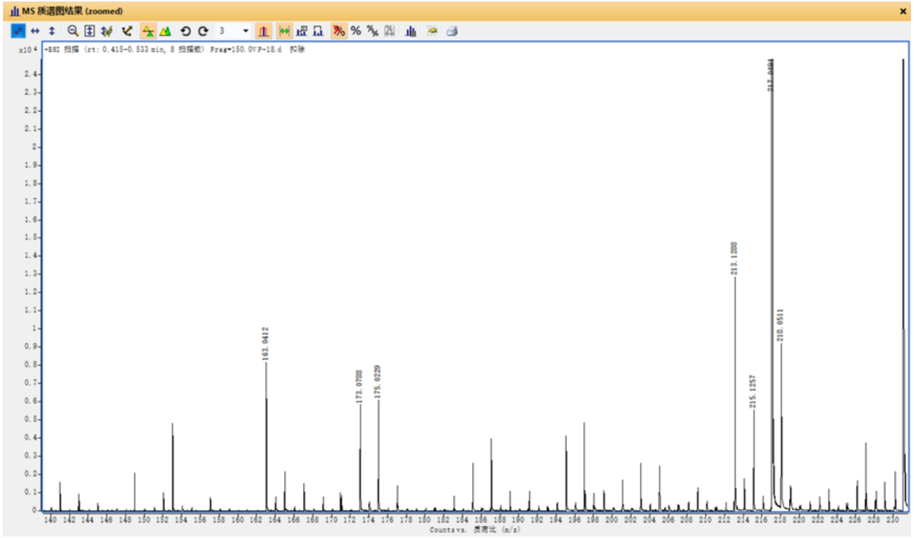

⁺ESI-MS

Compound **2**:

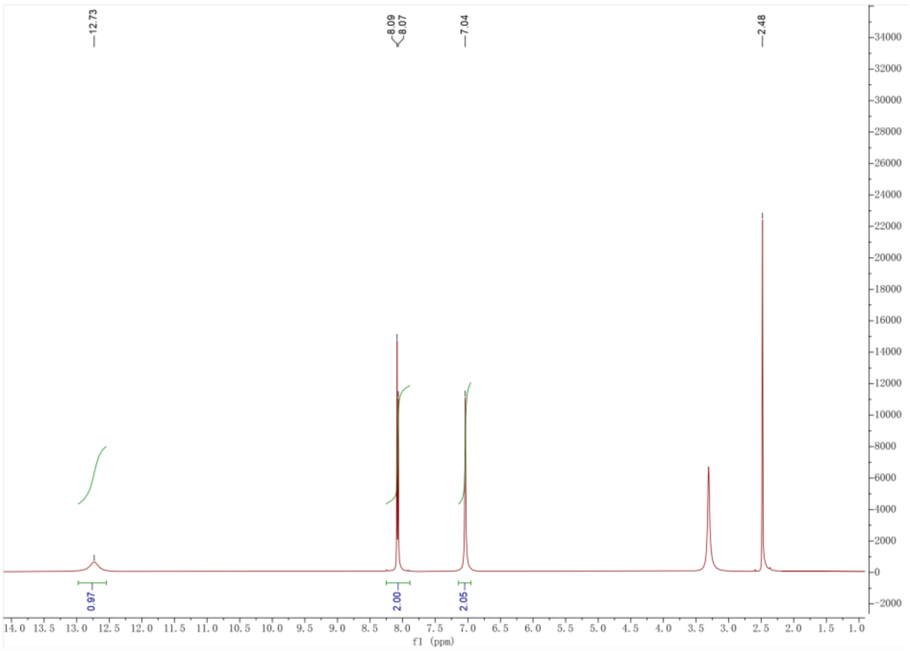

¹H-NMR spectrum（DMSO-*d6*, 600 MHz）

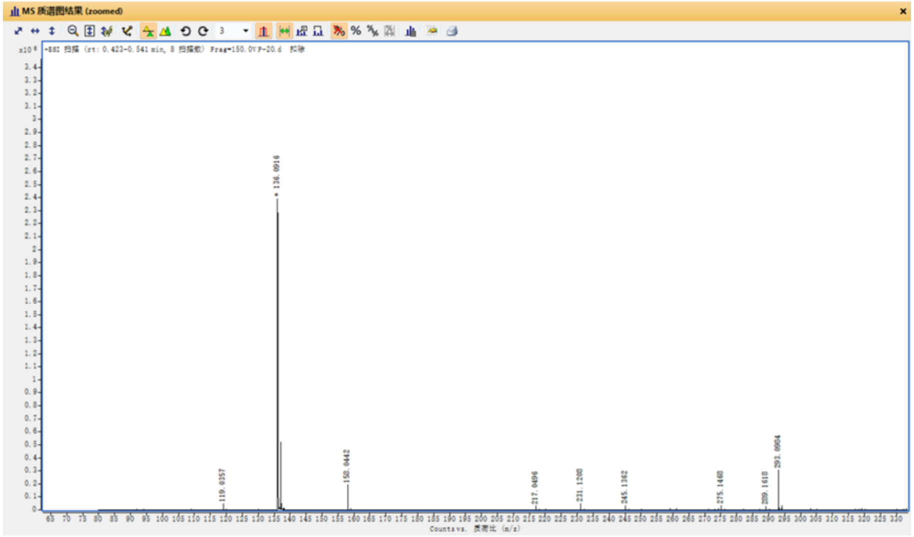

$^{+}$ESI-MS

Compound **3**:

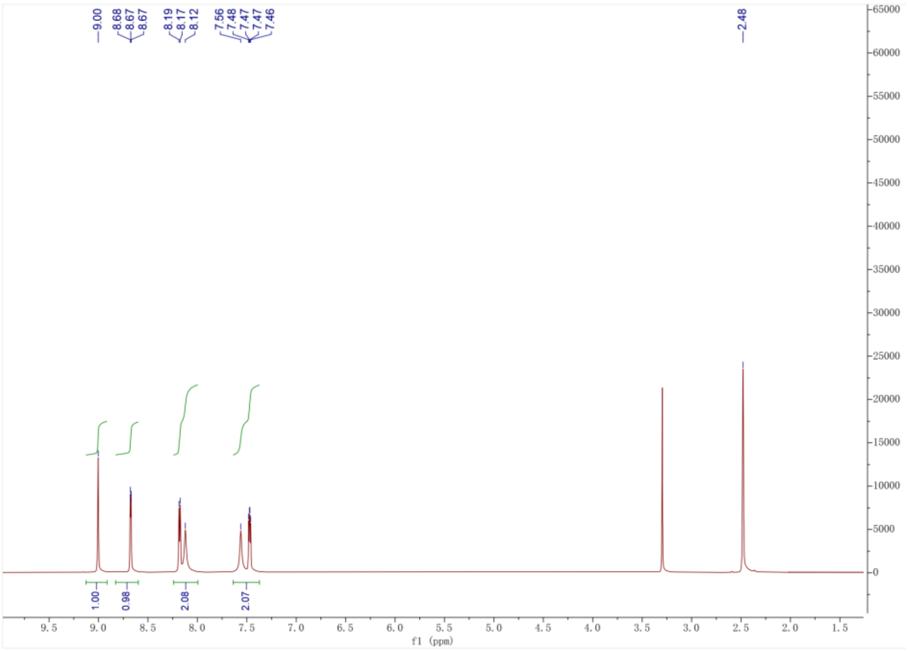

^{1}H-NMR spectrum（DMSO-*d*6, 600 MHz）

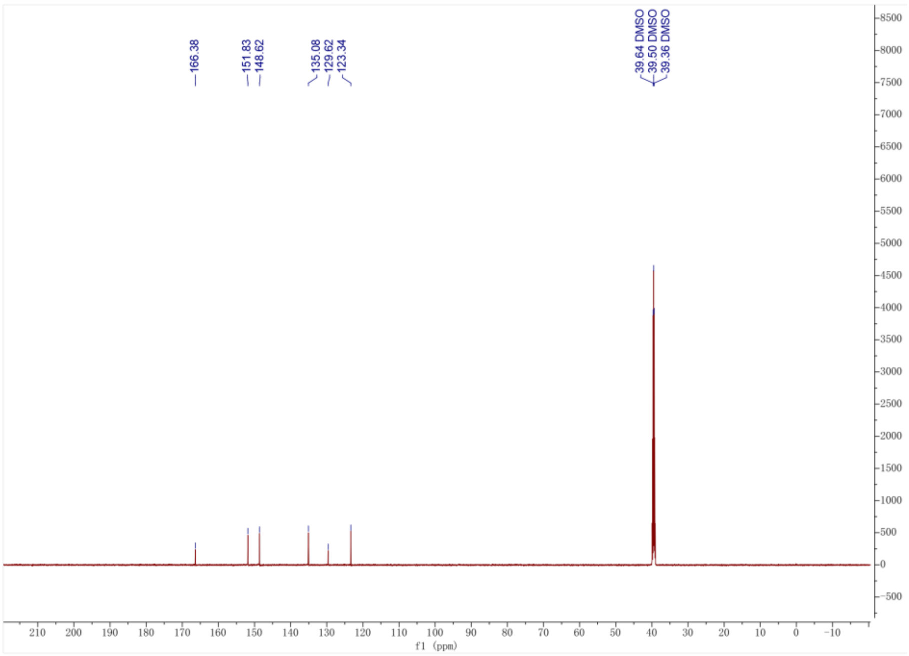

^{13}C-NMR spectrum（DMSO-*d*6, 150 MHz）

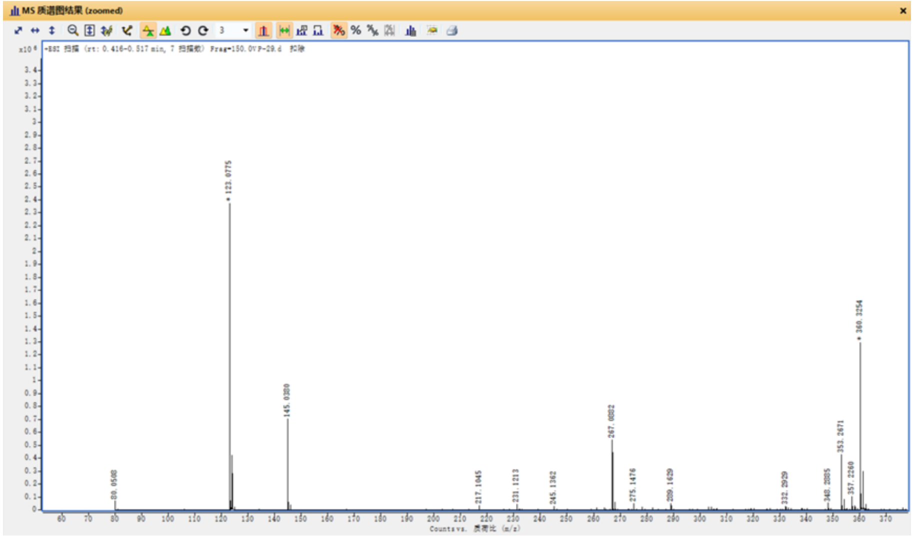

$^+$ESI-MS

Compound **4**:

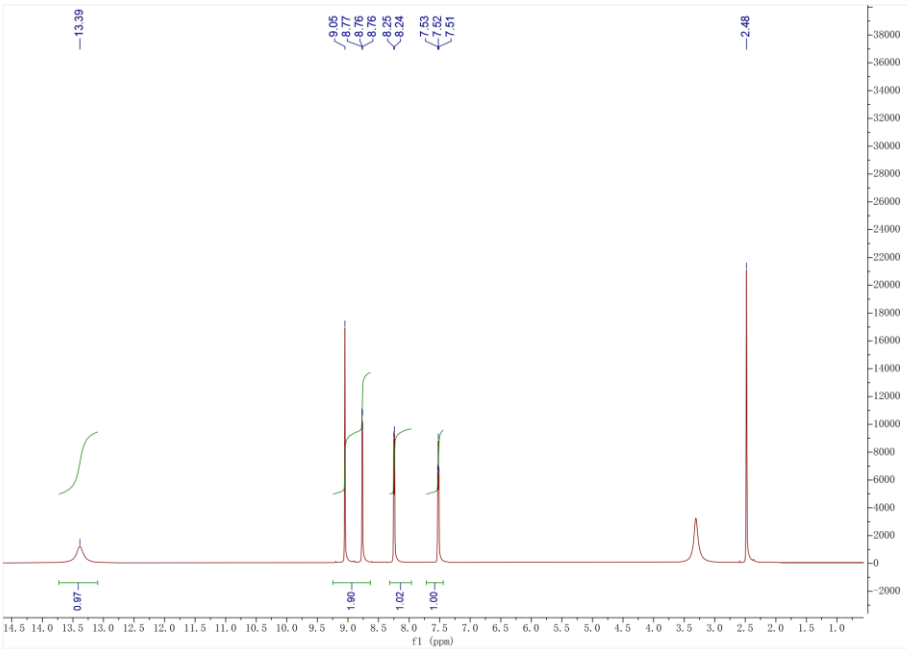

^{1}H-NMR spectrum（DMSO-*d*6, 600 MHz）

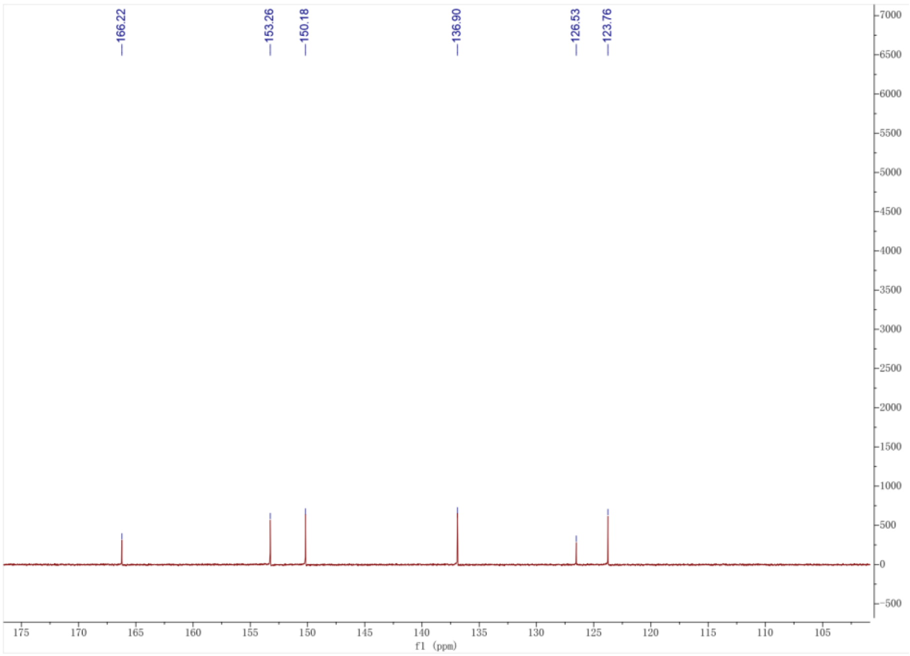

^{13}C-NMR spectrum（DMSO-*d*6, 150 MHz）

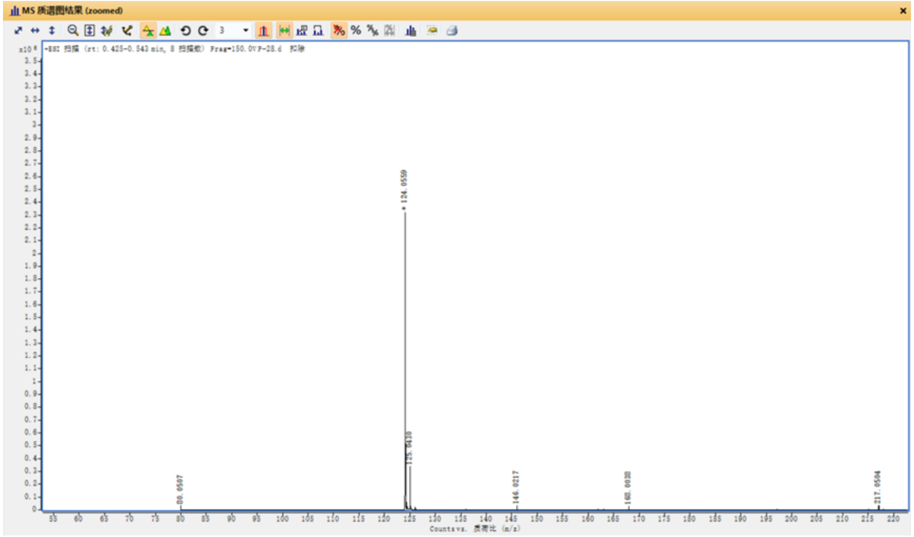
MS 质谱图结果 (zoomed)
x10^6
+ESI 扫描 (rt: 0.425-0.543 min, 8 扫描数) Frag=150.0V P-28.d 扣除
* 124.0559
125.0430
80.0507
146.0217
168.0038
217.0504
Counts vs. 质荷比 (m/z)

$^{+}$ESI-MS

Compound **5**:

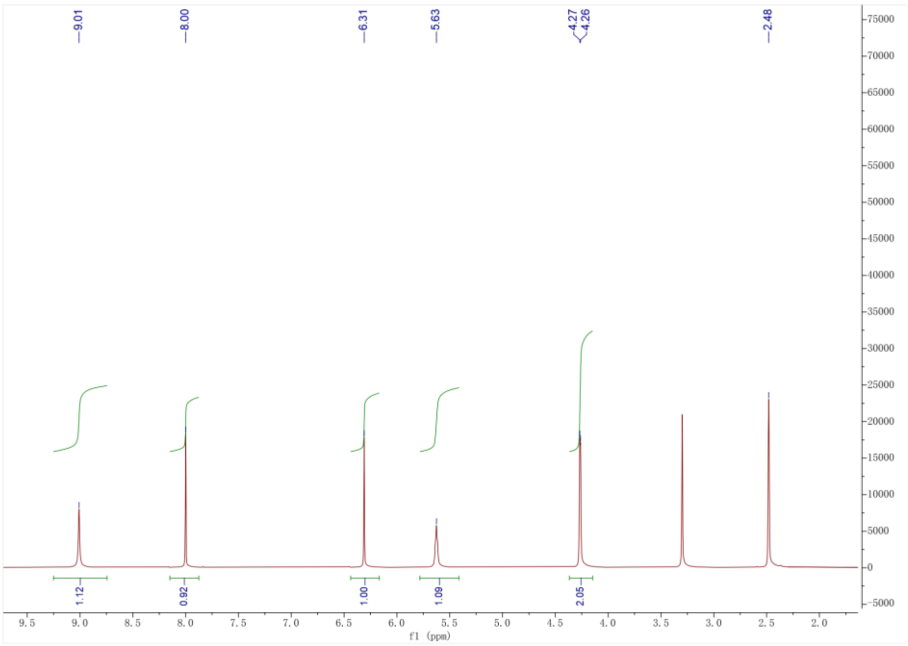

^{1}H-NMR spectrum（DMSO-*d*6, 600 MHz）

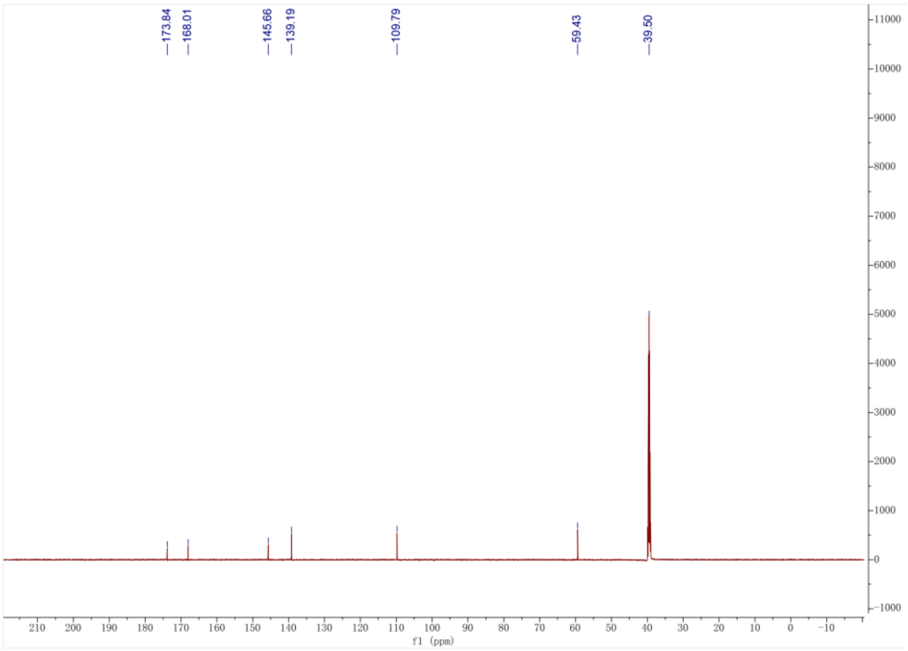

^{13}C-NMR spectrum（DMSO-*d*6, 150 MHz）

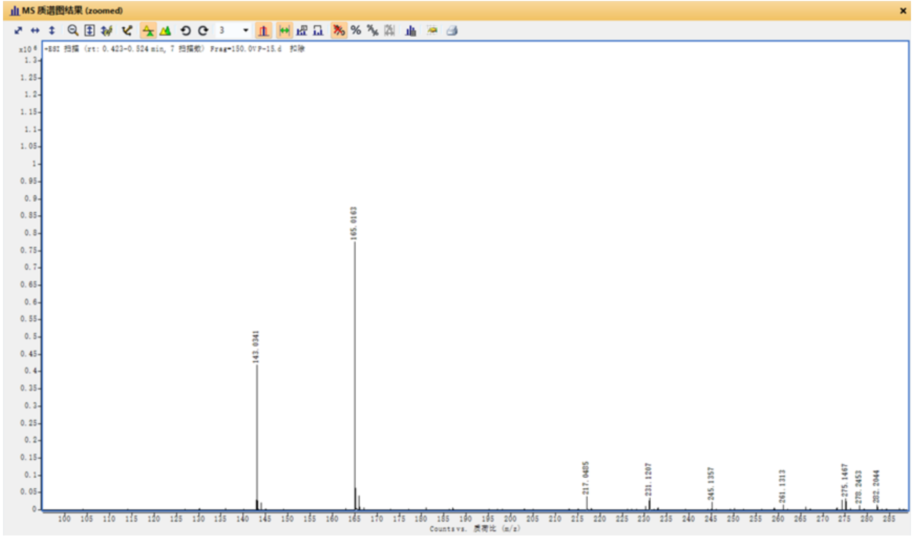
MS 质谱图结果 (zoomed)
+ESI 扫描 (rt: 0.423-0.524 min, 7 扫描数) Frag=150.0V P-15.d 扣除
x10^6
143.0341
165.0163
217.0485
231.1297
245.1357
261.1313
275.1467
278.2453
282.2044
Counts vs. 质荷比 (m/z)
$^{+}$ESI-MS

Compound **6**:

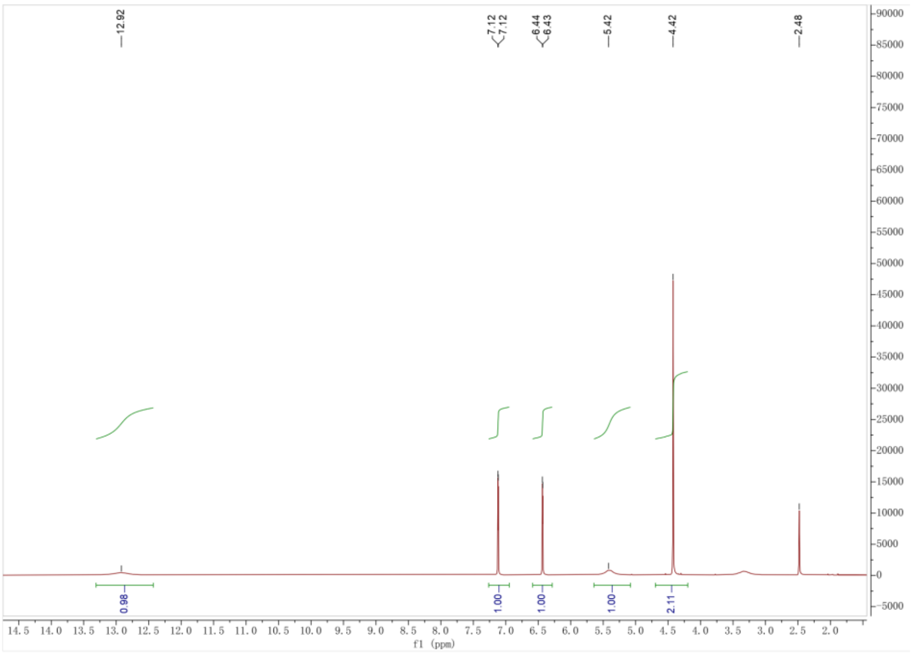

^{1}H-NMR spectrum（DMSO-*d*6, 600 MHz）

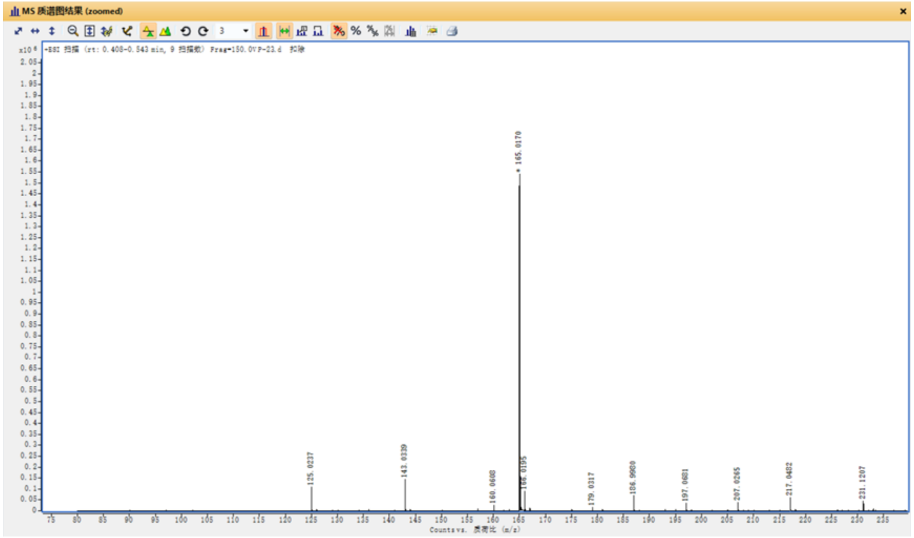

$^+$ESI-MS

Compound **7**:

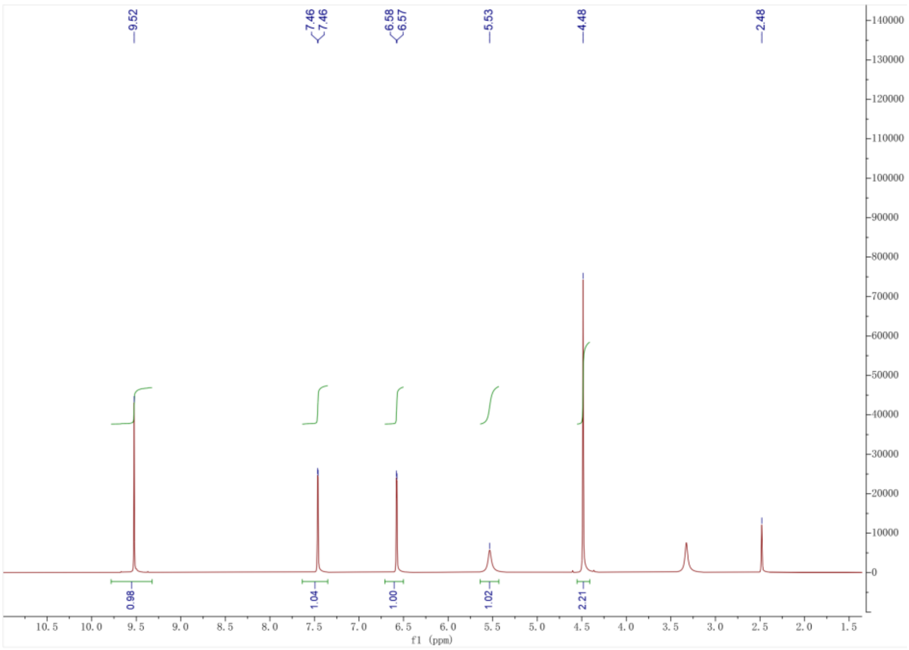

^{1}H-NMR spectrum（DMSO-*d*6, 600 MHz）

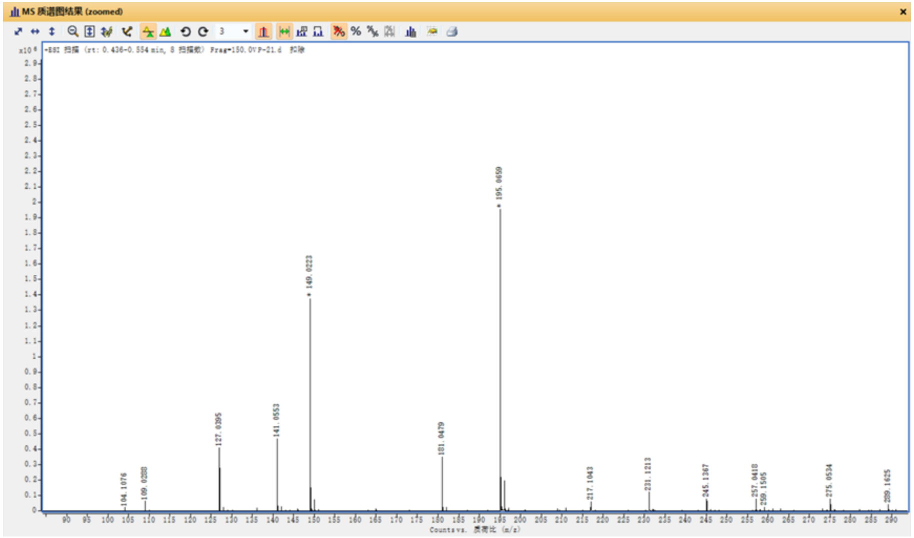

$^{+}$ESI-MS

Compound **8**:

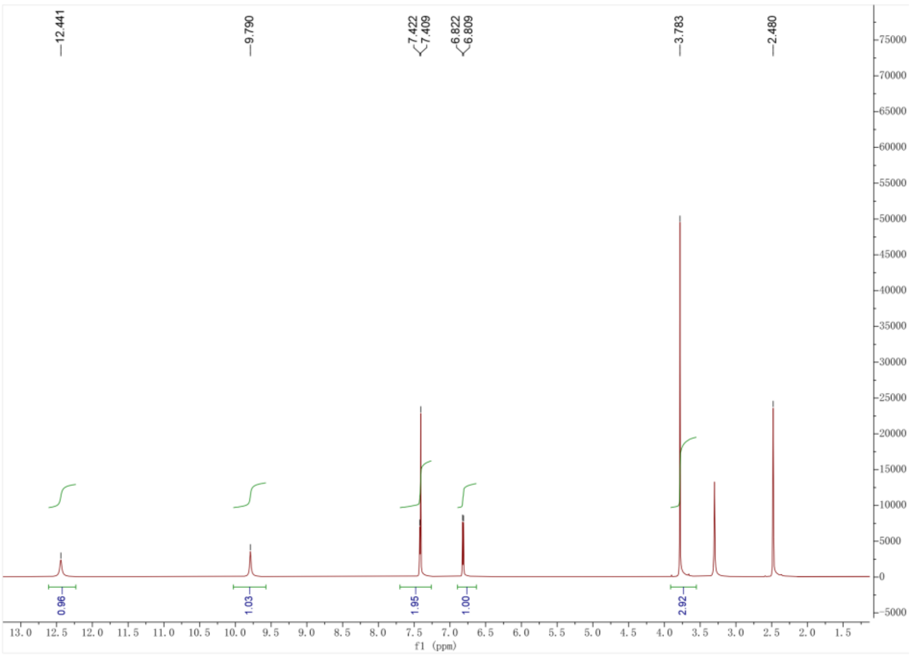

^{1}H-NMR spectrum（DMSO-*d*6, 600 MHz）

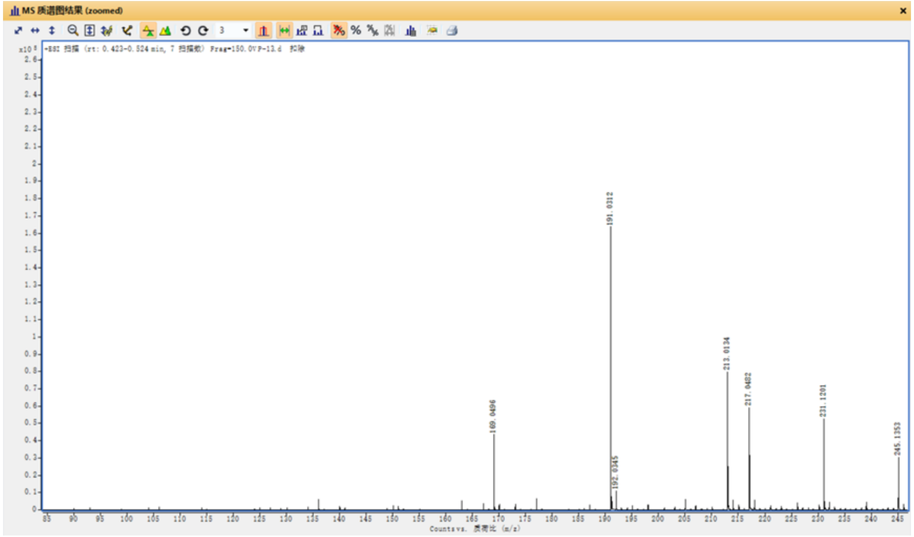

[+]ESI-MS

Compound **9**:

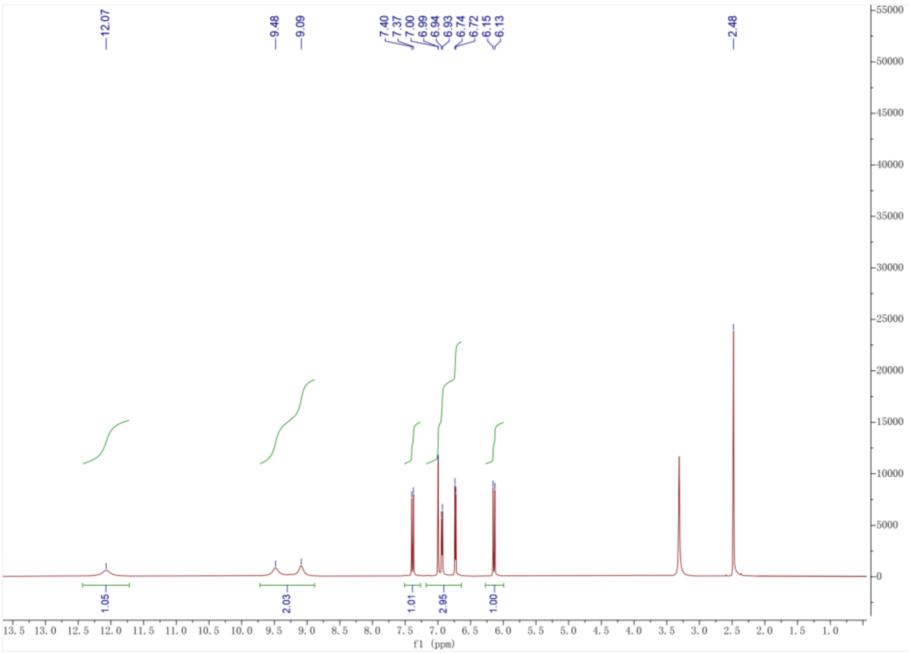

^{1}H-NMR spectrum（DMSO-*d*6, 600 MHz）

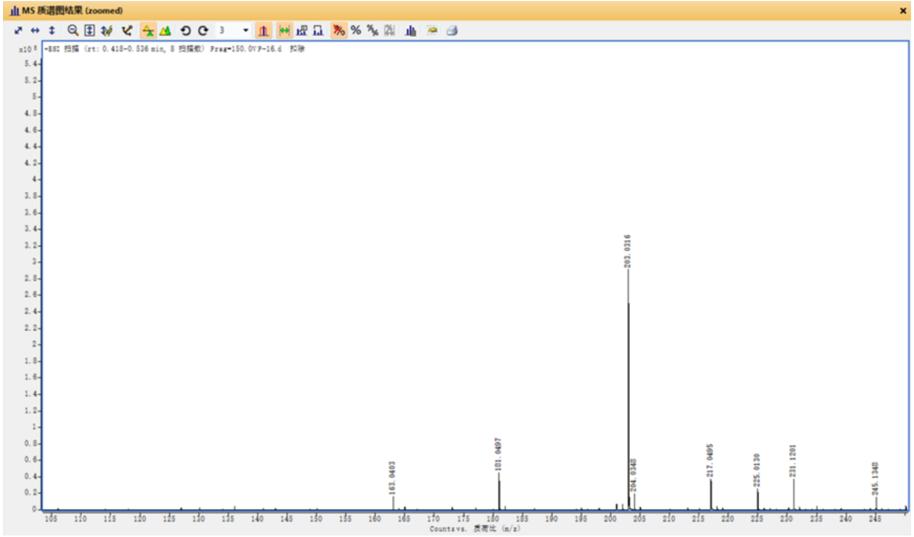

+ESI-MS

Compound **10**：

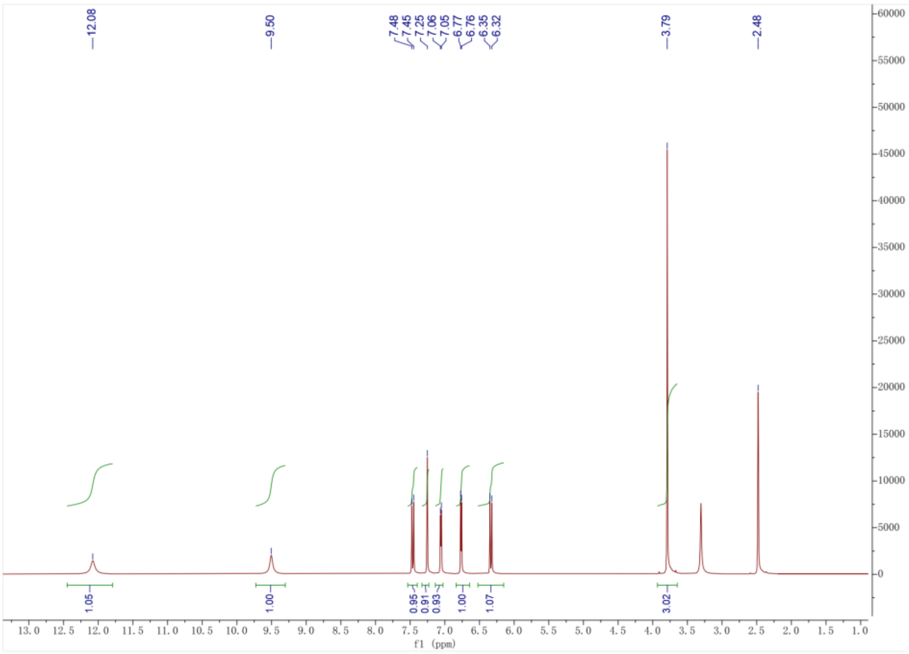

^{1}H-NMR spectrum（DMSO-*d*6, 600 MHz）

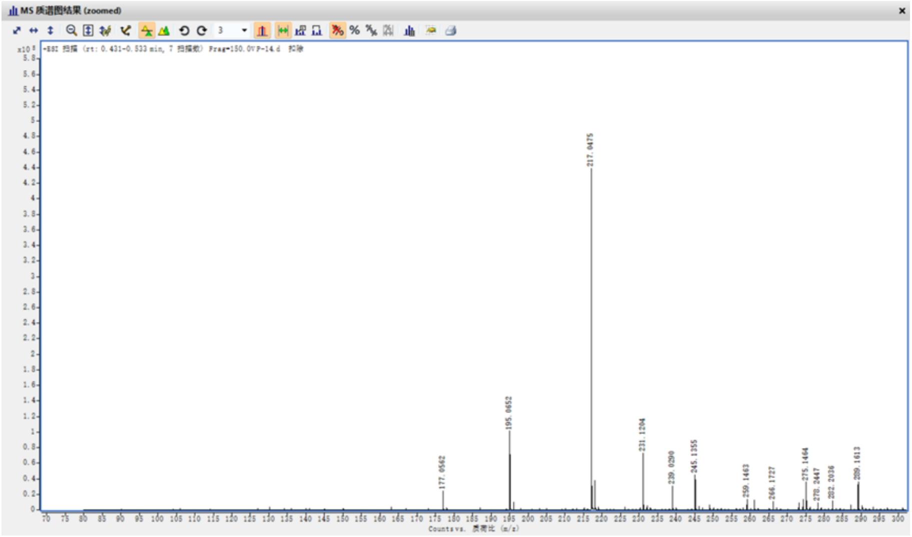

$^{+}$ESI-MS

Compound **11**：

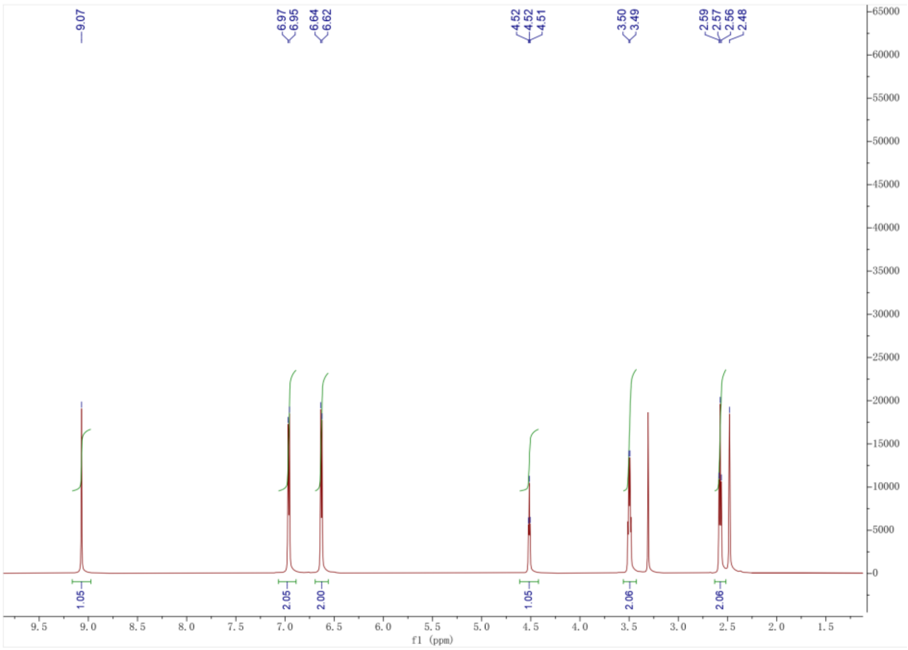

^{1}H-NMR spectrum（DMSO-*d*6, 600 MHz）

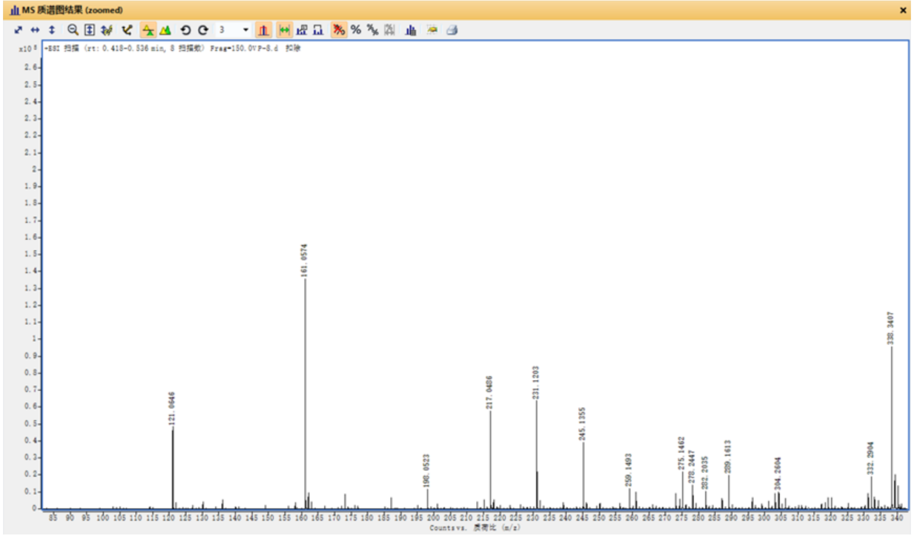

$^{+}$ESI-MS

Compound **12**：

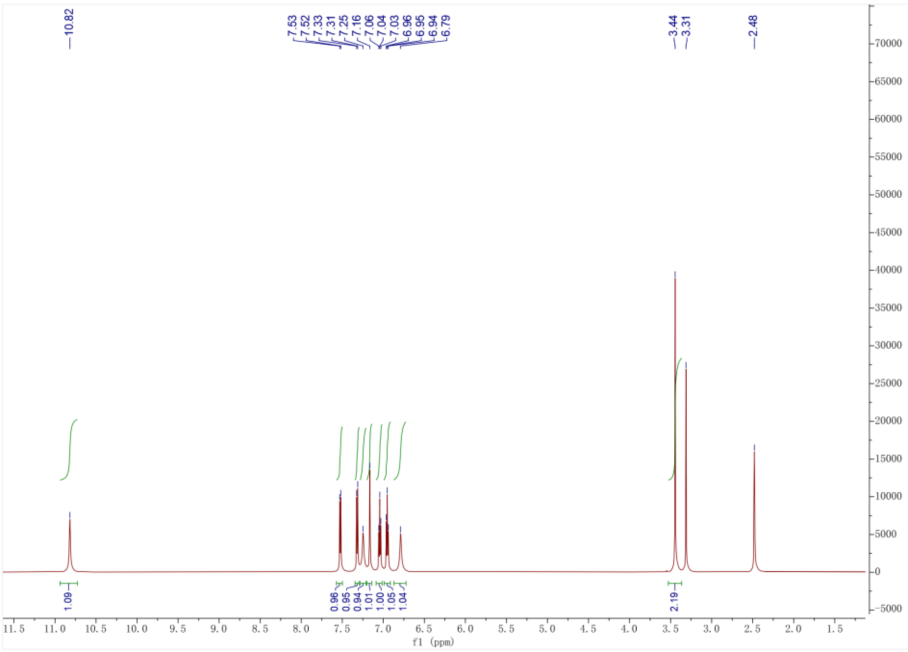

^{1}H-NMR spectrum（DMSO-*d*6, 600 MHz）

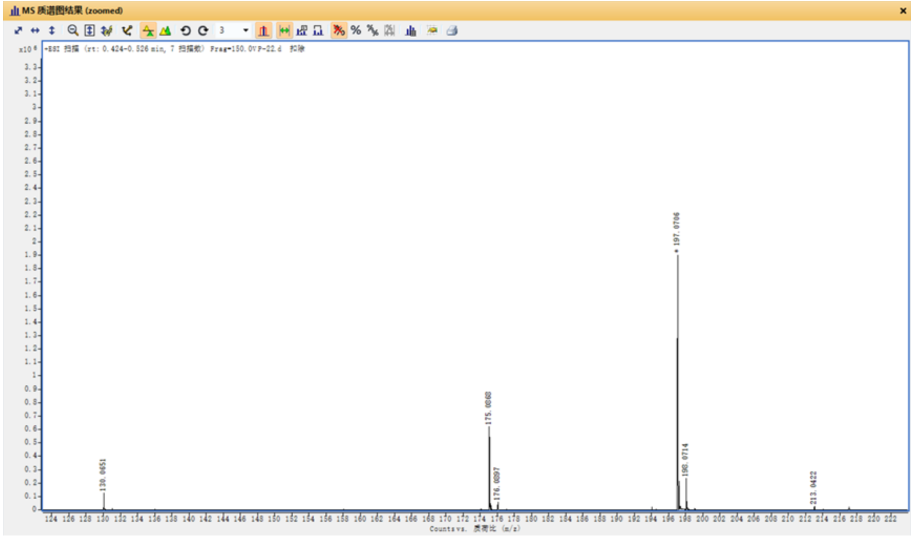

$^+$ESI-MS

Compound **13**:

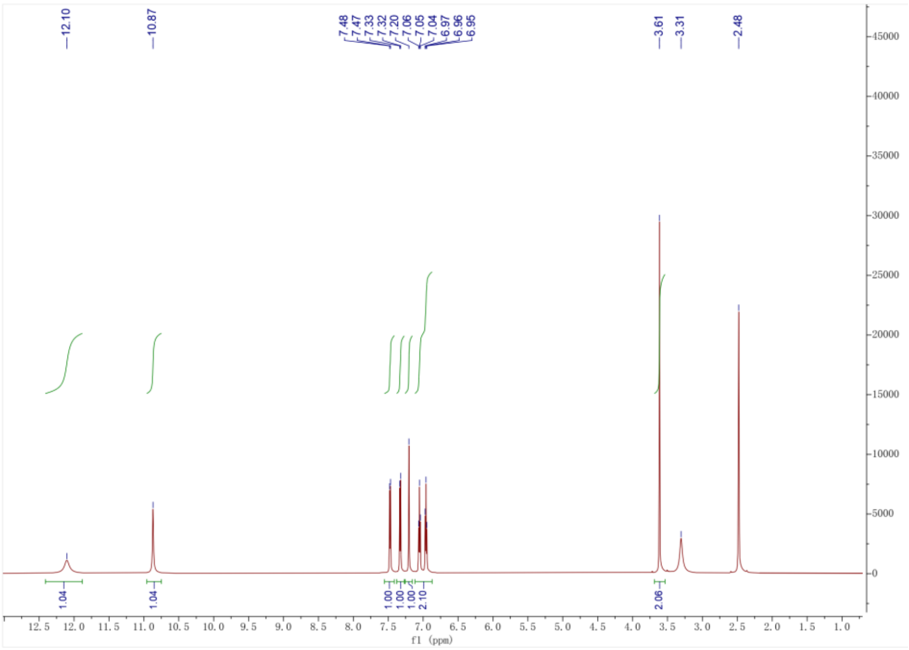

^{1}H-NMR spectrum（DMSO-*d*6, 600 MHz）

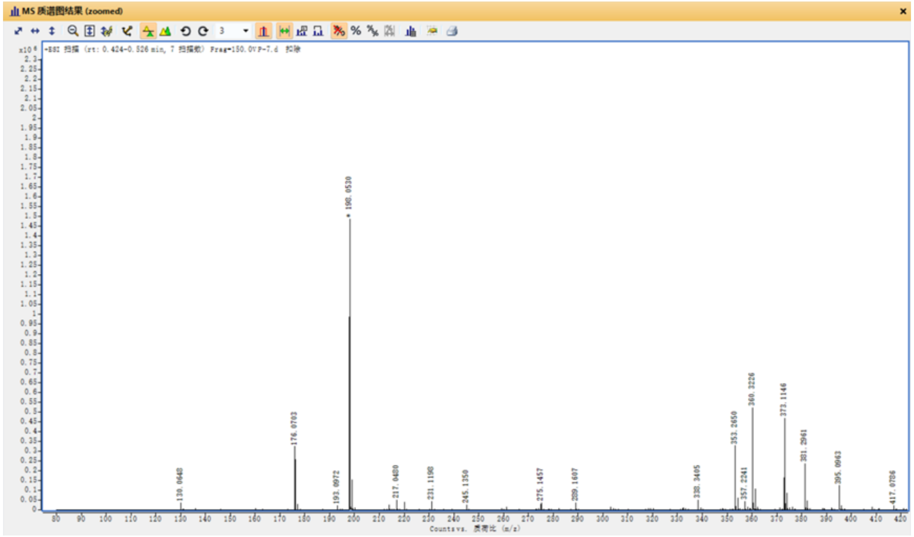

$^{+}$ESI-MS

Compound **14**:

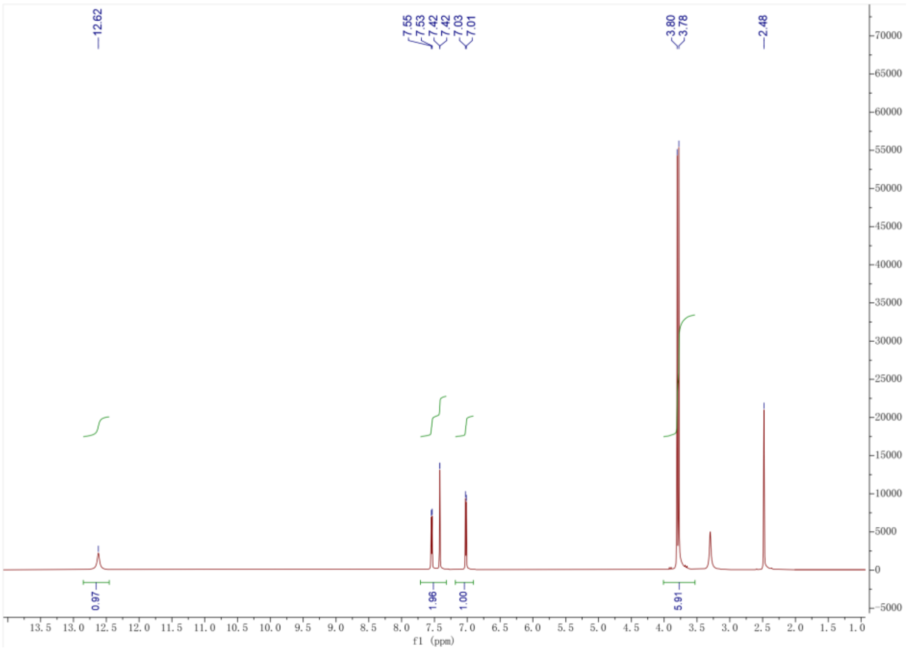

^{1}H-NMR spectrum（DMSO-*d*6, 600 MHz）

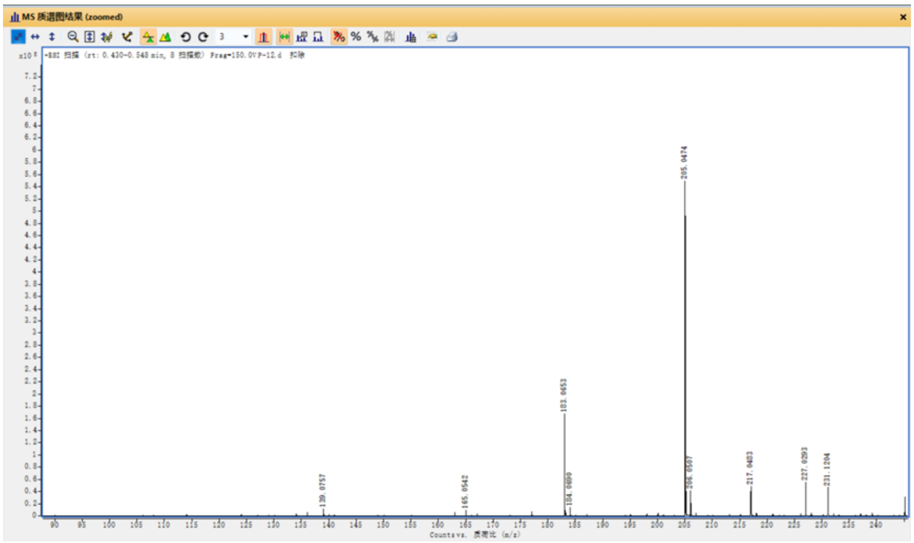

+ESI-MS

Compound **15**:

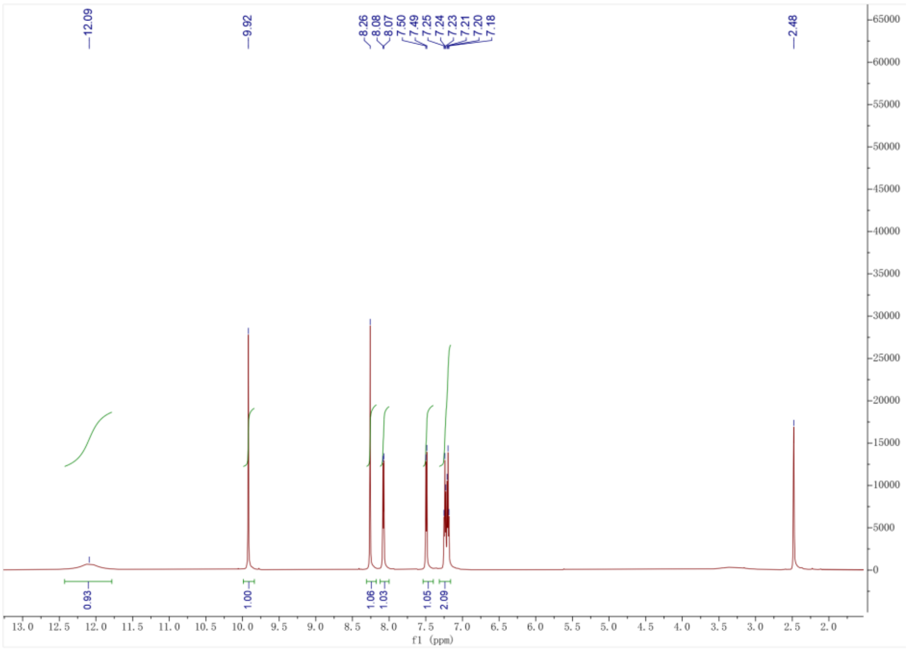

^{1}H-NMR spectrum（DMSO-*d*6, 600 MHz）

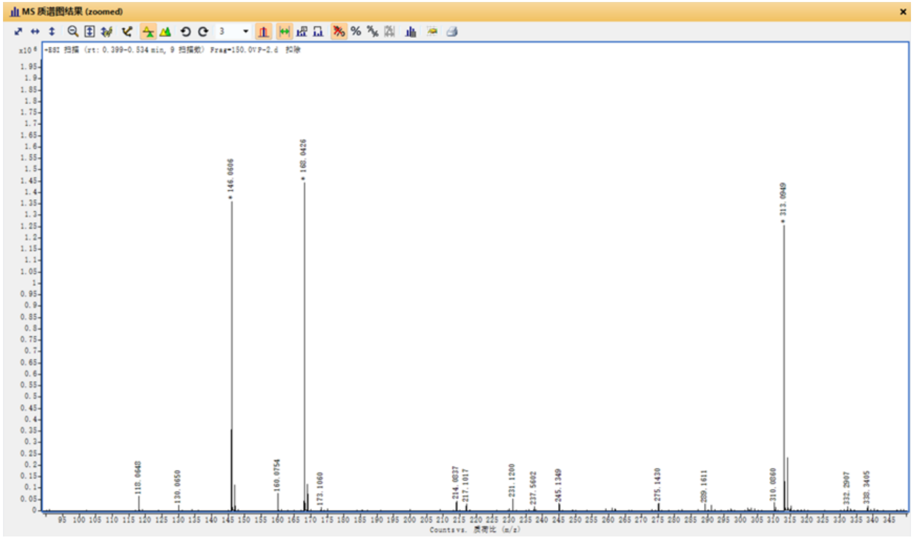

$^{+}$ESI-MS

Compound **16**:

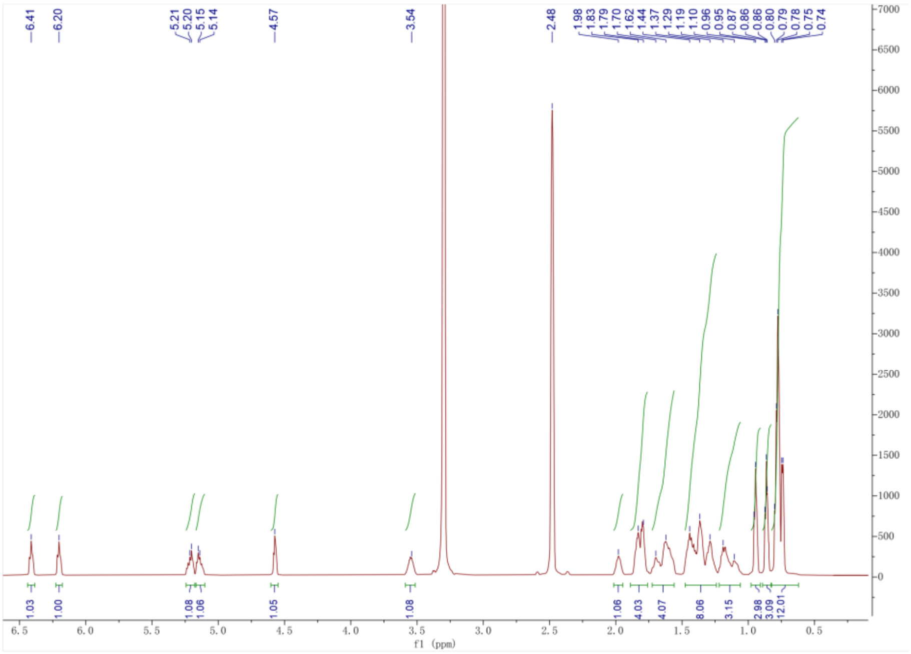

^{1}H-NMR spectrum（DMSO-*d*6, 600 MHz）

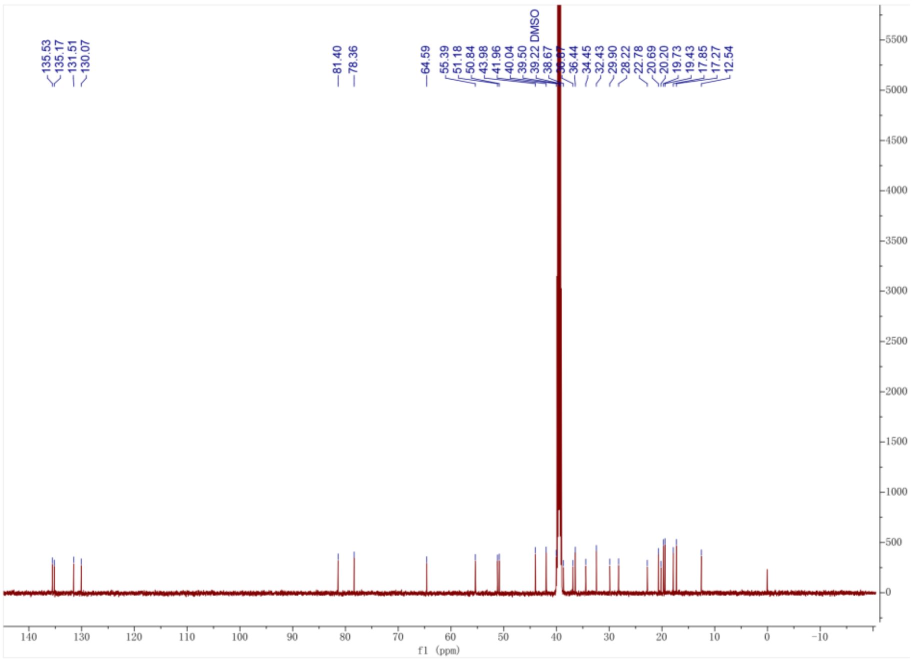

^{13}C-NMR spectrum（DMSO-*d*6, 150 MHz）

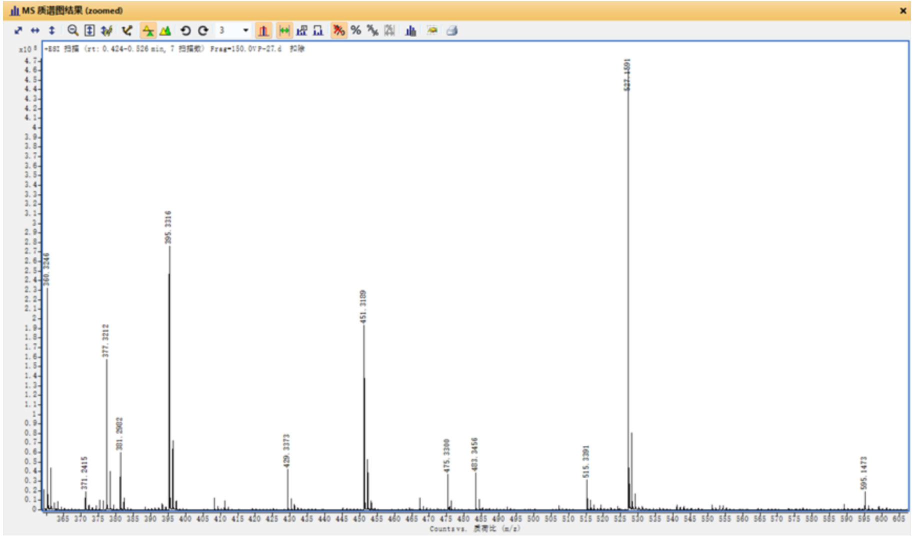
MS 质谱图结果 (zoomed)
x10 5
+ESI 扫描 (rt: 0.424-0.526 min, 7 扫描数) Frag=150.0V P-27.d 扣除
360.3246
371.2415
377.3212
381.2982
395.3316
429.3373
451.3189
475.3300
483.3456
515.3391
527.1591
595.1473
Counts vs. 质荷比 (m/z)

$^{+}$ESI-MS

Compound **17**:

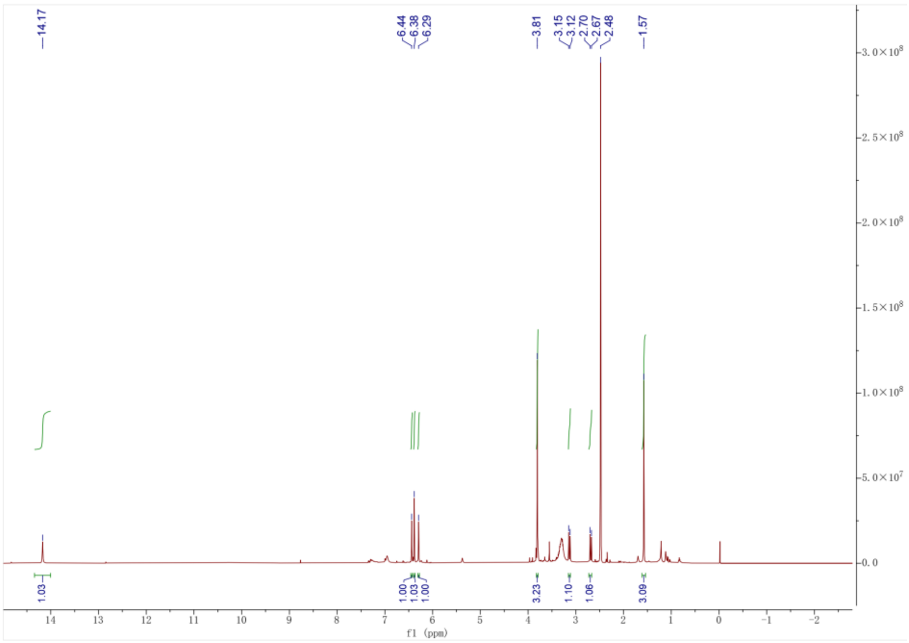

^{1}H-NMR spectrum（DMSO-$d6$, 600 MHz）

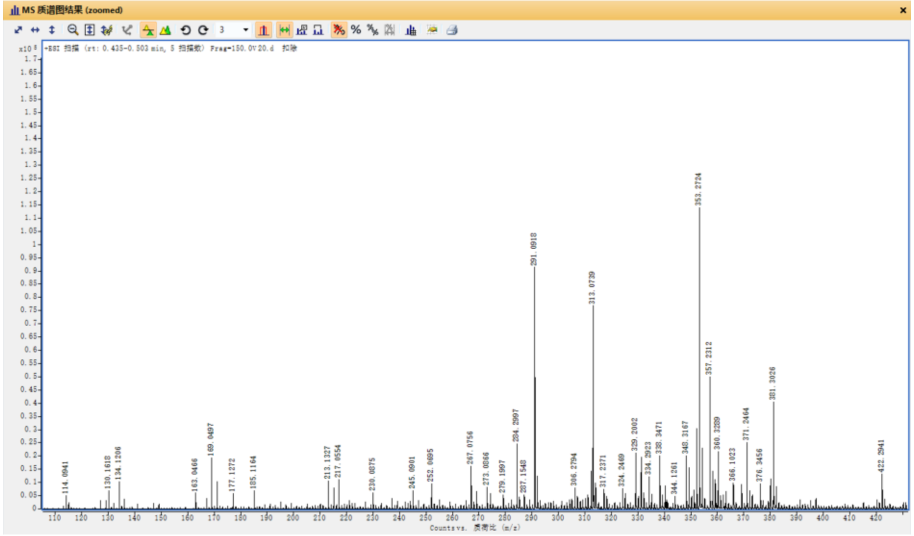

$^{+}$ESI-MS

Compound **18**:

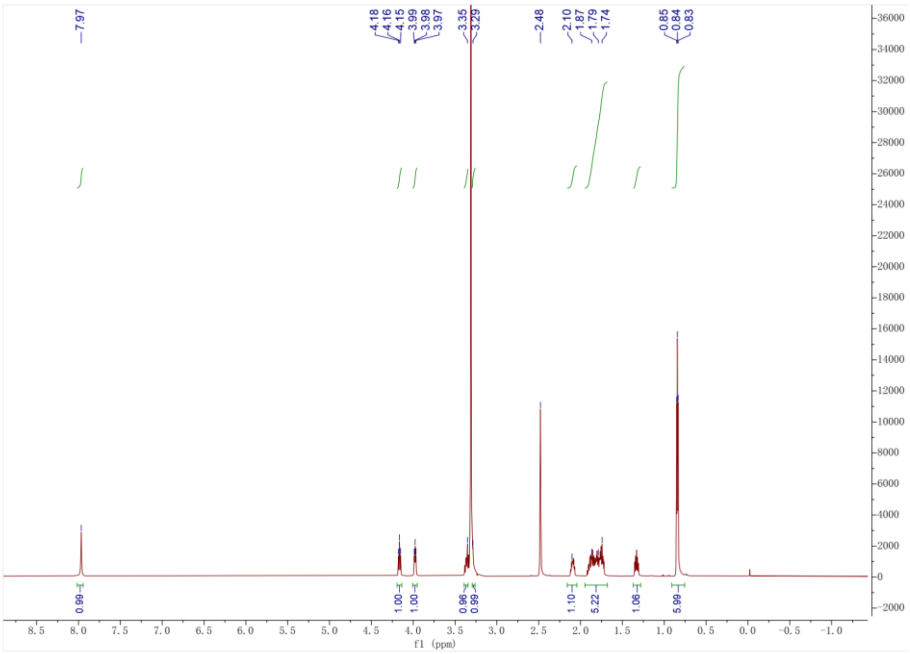

^{1}H-NMR spectrum（DMSO-*d*6, 600 MHz）

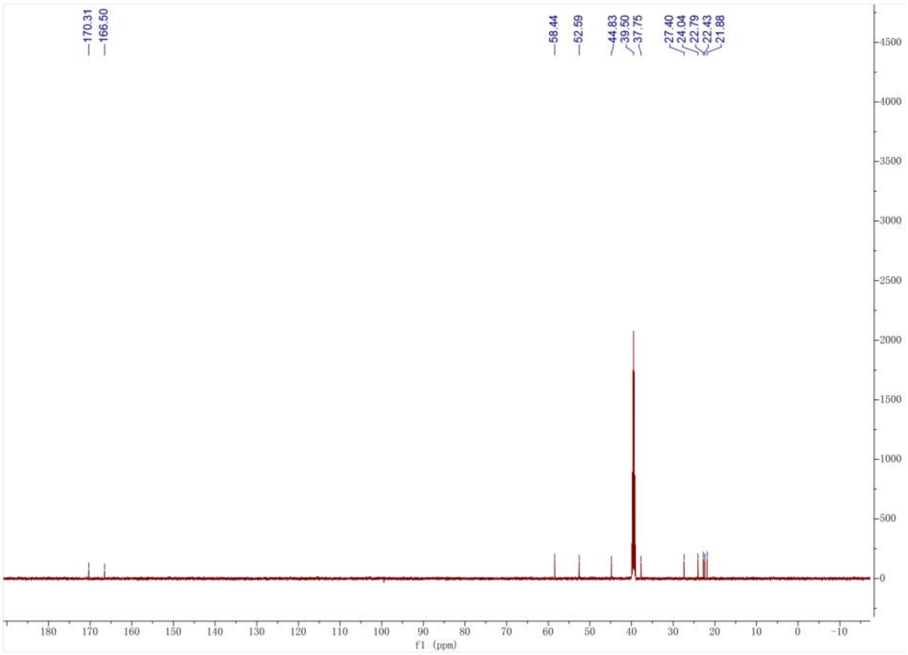

^{13}C-NMR spectrum（DMSO-*d*6, 150 MHz）

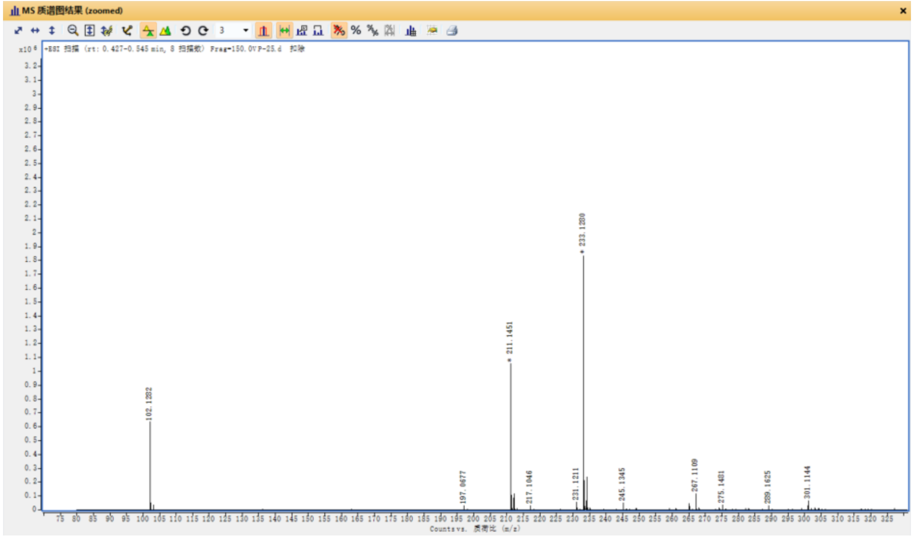
MS 质谱图结果 (zoomed)
x10 6
+ESI 扫描 (rt: 0.427-0.545 min, 8 扫描数) Frag=150.0V P-25.d 扣除
102.1232
197.0677
211.1451
217.1046
231.1211
233.1280
245.1345
267.1109
275.1481
289.1625
301.1144
Counts vs. 质荷比 (m/z)

$^{+}$ESI-MS

Compound **19**:

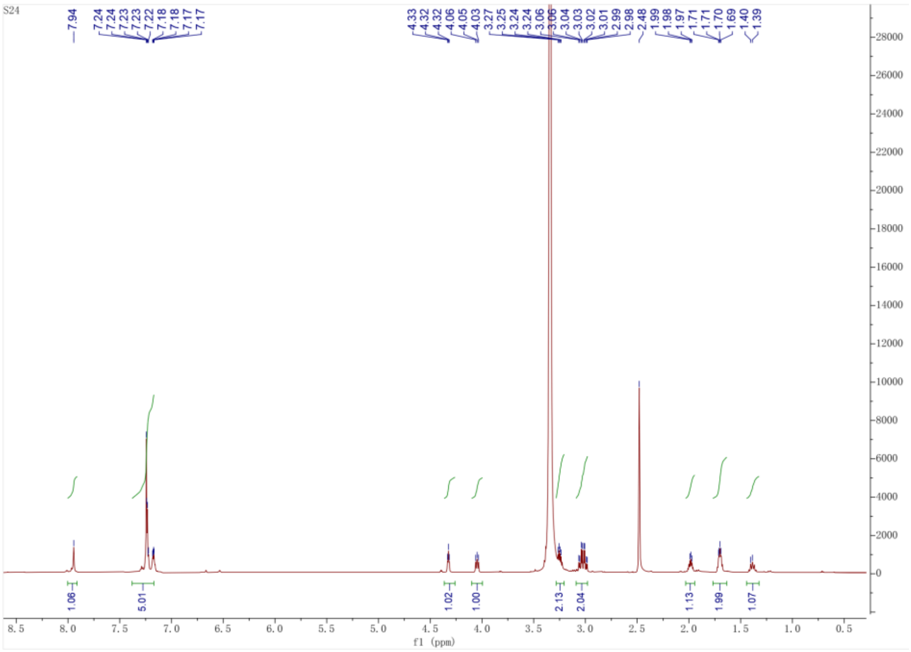

^{1}H-NMR spectrum（DMSO-*d*6, 600 MHz）

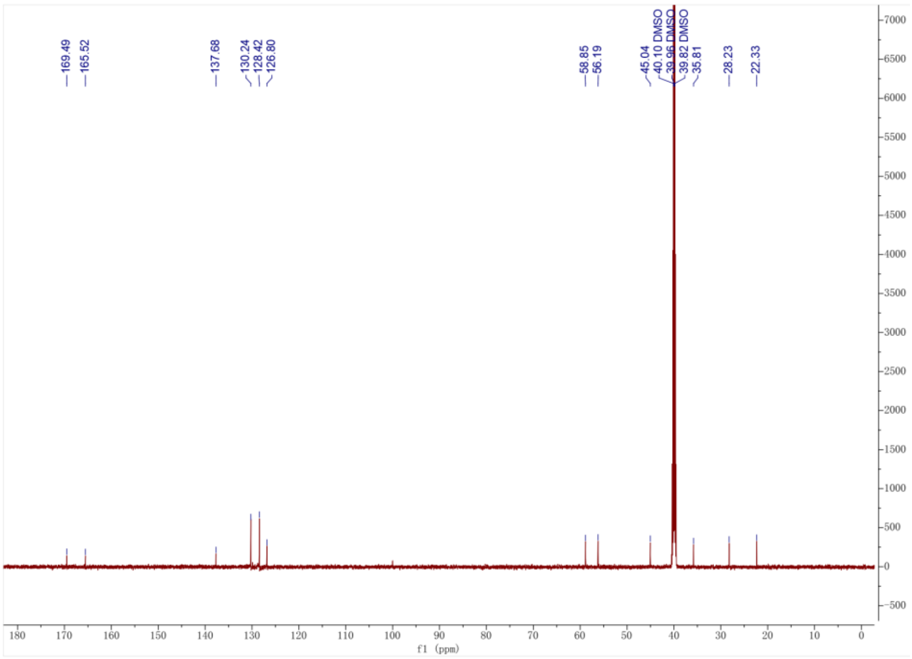

^{13}C-NMR spectrum（DMSO-*d*6, 150 MHz）

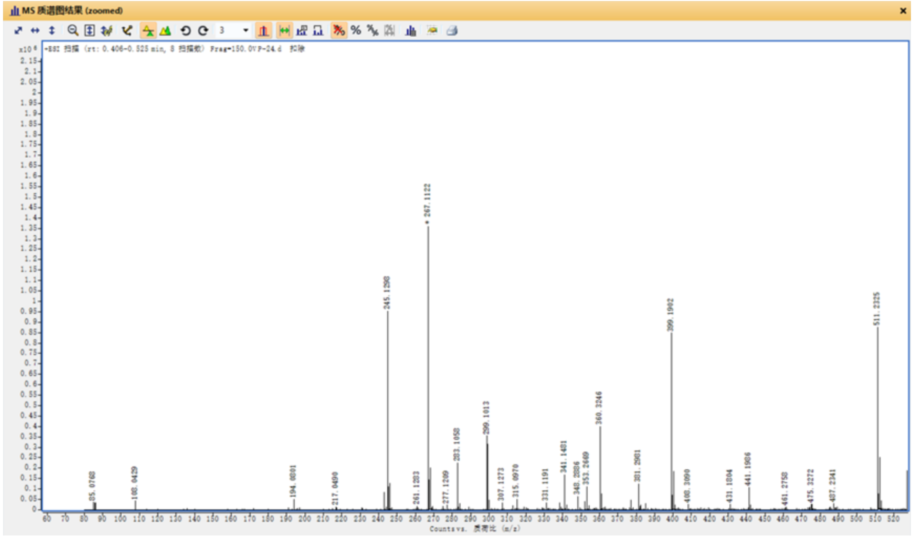

+ESI-MS

Compound **20**：

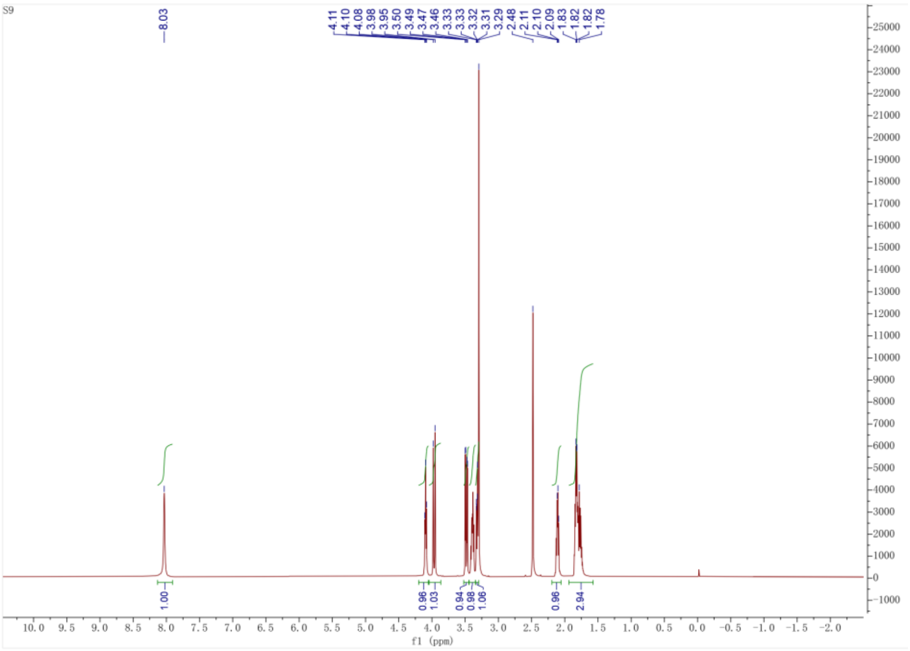

^{1}H-NMR spectrum（DMSO-*d*6, 600 MHz）

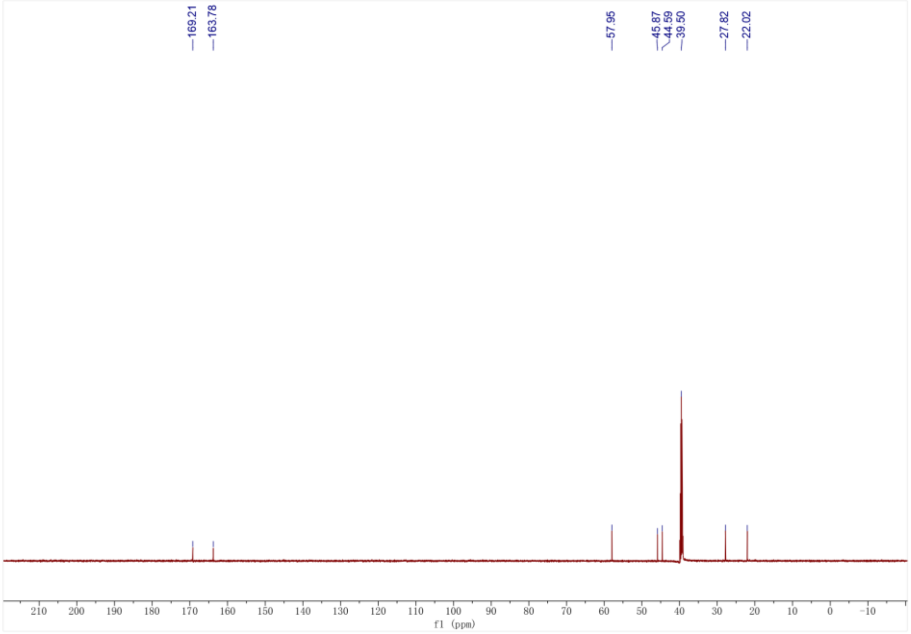

^{13}C-NMR spectrum（DMSO-*d*6, 150 MHz）

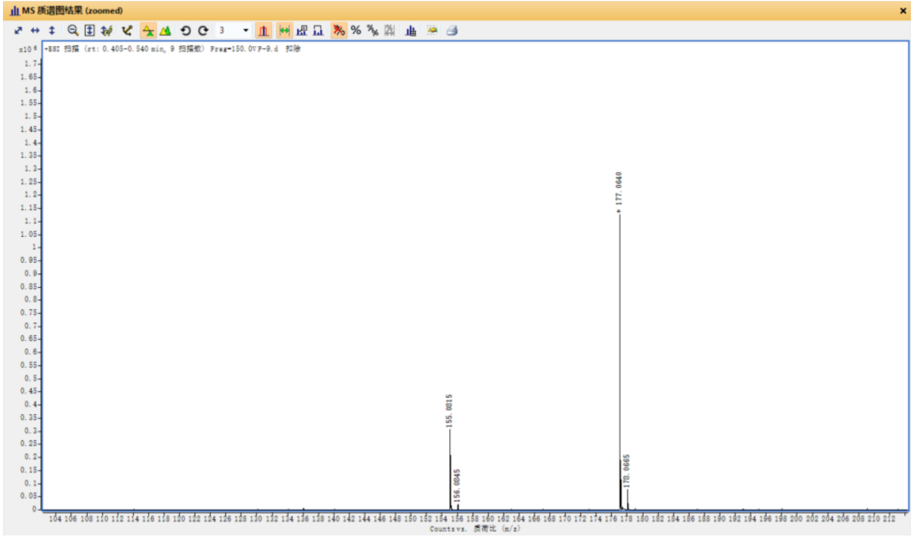

$^{+}$ESI-MS

Compound **21**：

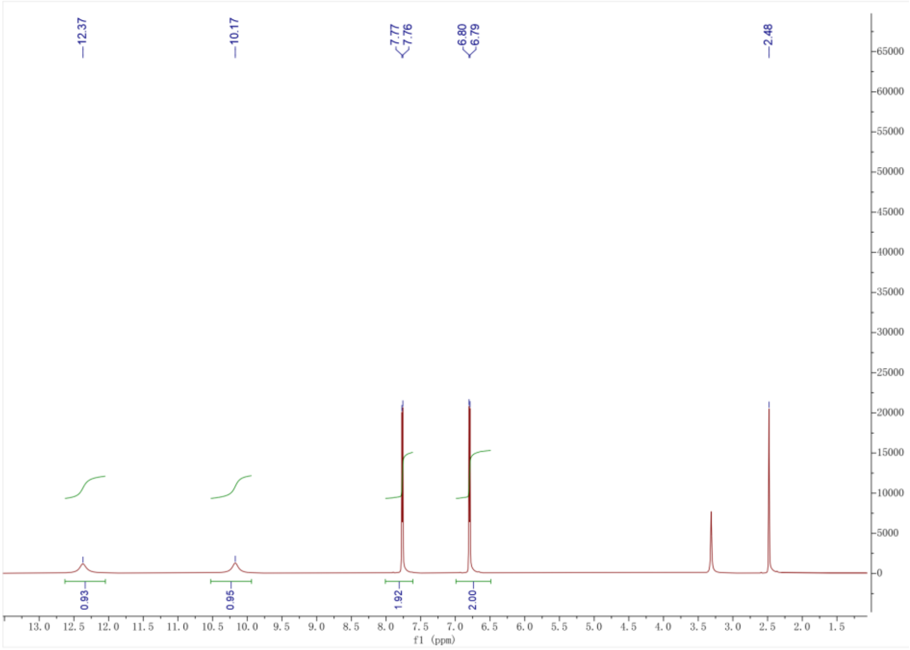

^{1}H-NMR spectrum（DMSO-*d6*, 600 MHz）

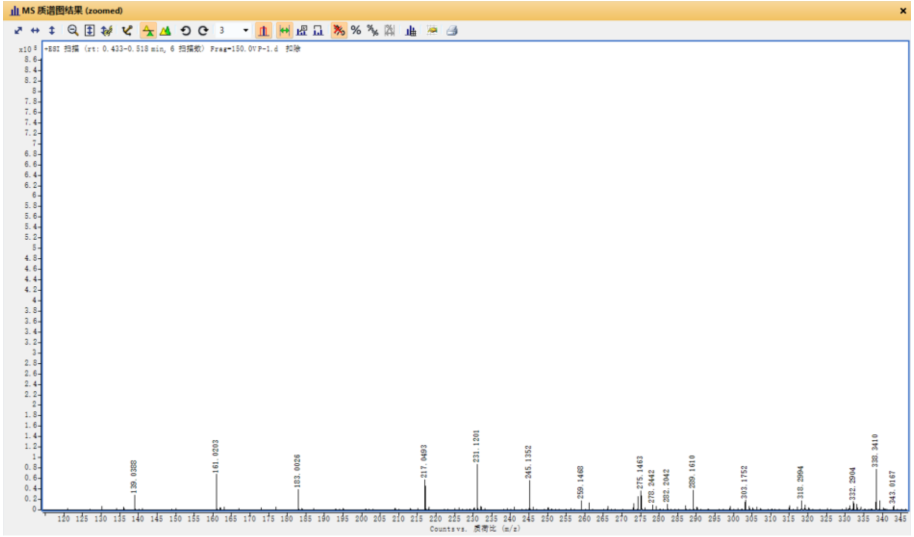

+ESI-MS

Compound **22**:

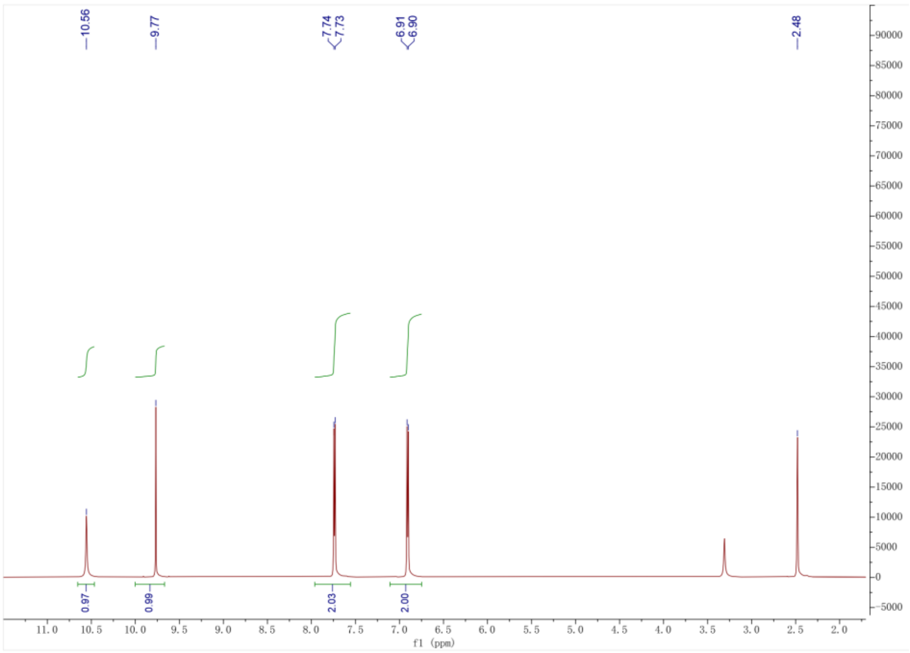

^{1}H-NMR spectrum（DMSO-*d6*, 600 MHz）

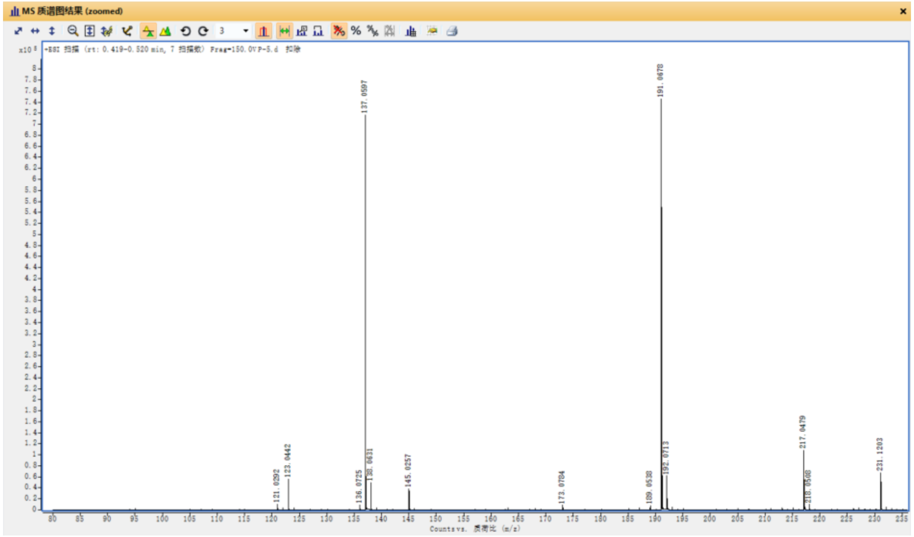

+ESI-MS

Compound **23**:

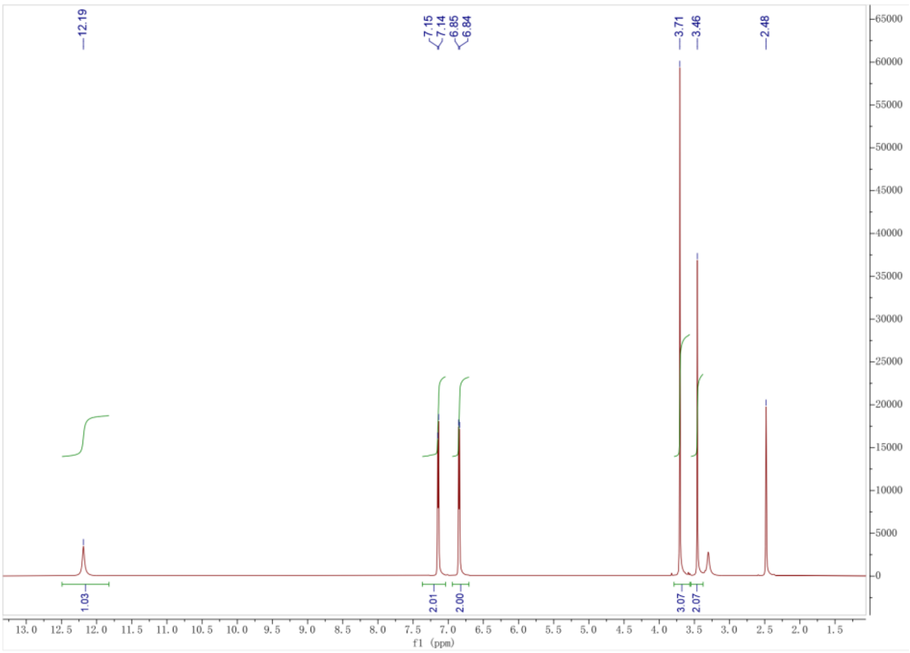

¹H-NMR spectrum（DMSO-*d*6, 600 MHz）

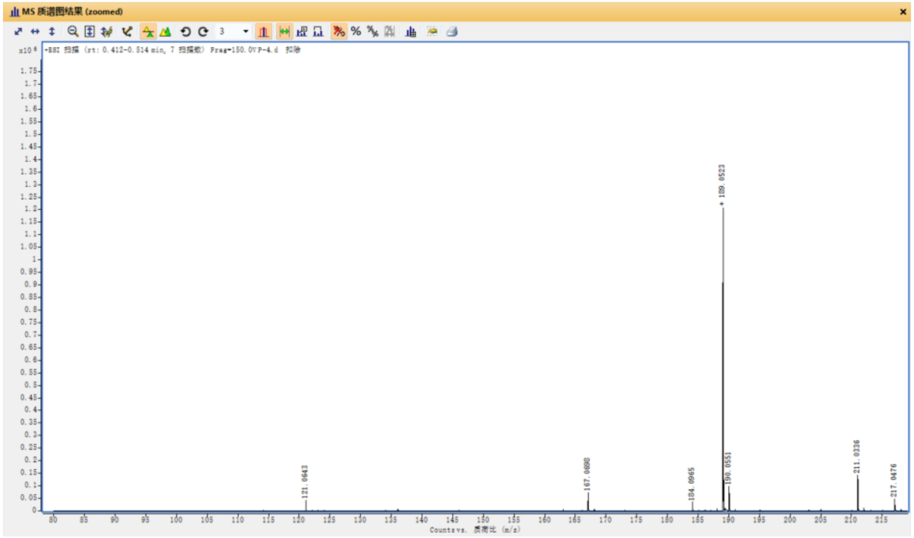

⁺ESI-MS

Compound **24**:

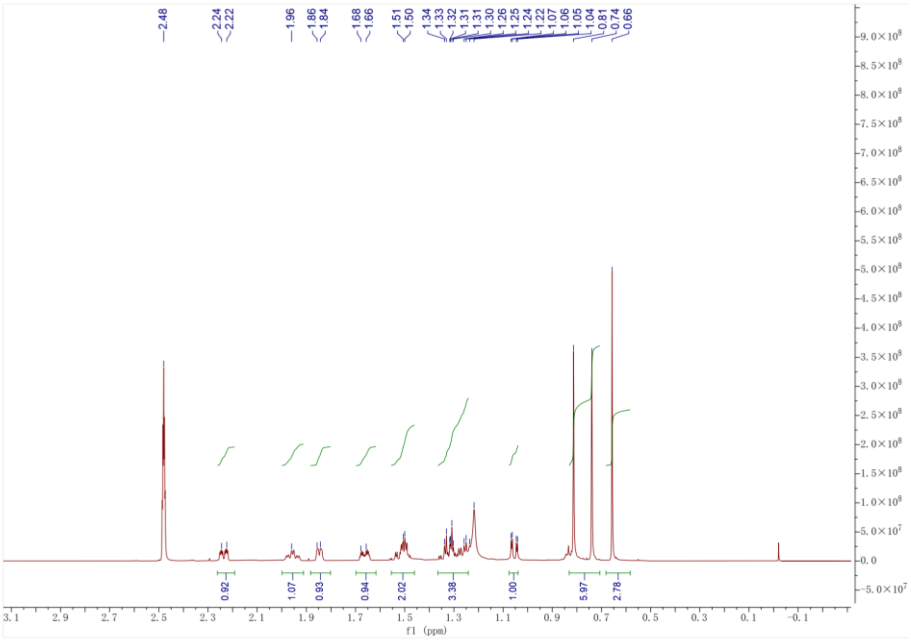

^{1}H-NMR spectrum（DMSO-*d*6, 600 MHz）

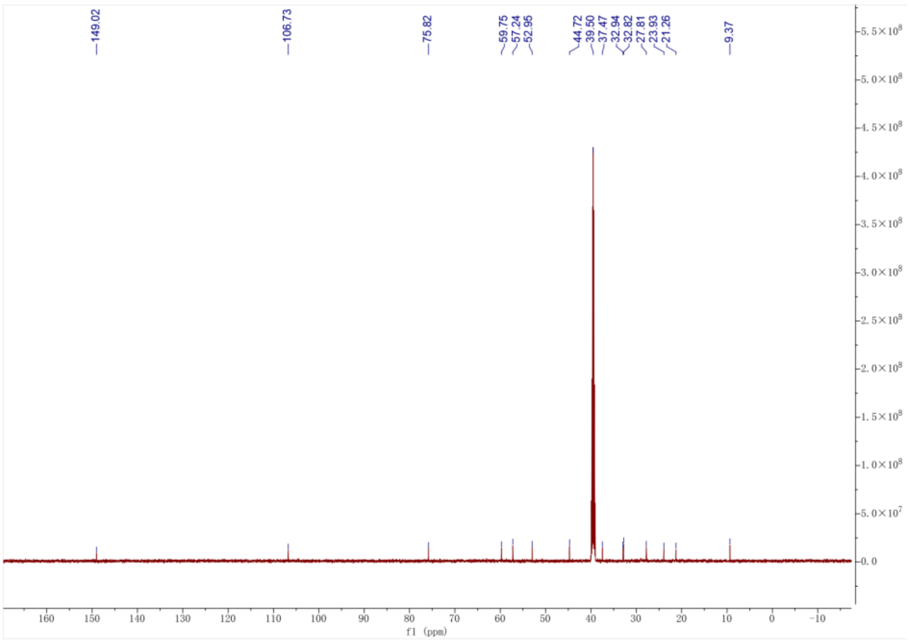

^{13}C-NMR spectrum（DMSO-*d*6, 150 MHz）

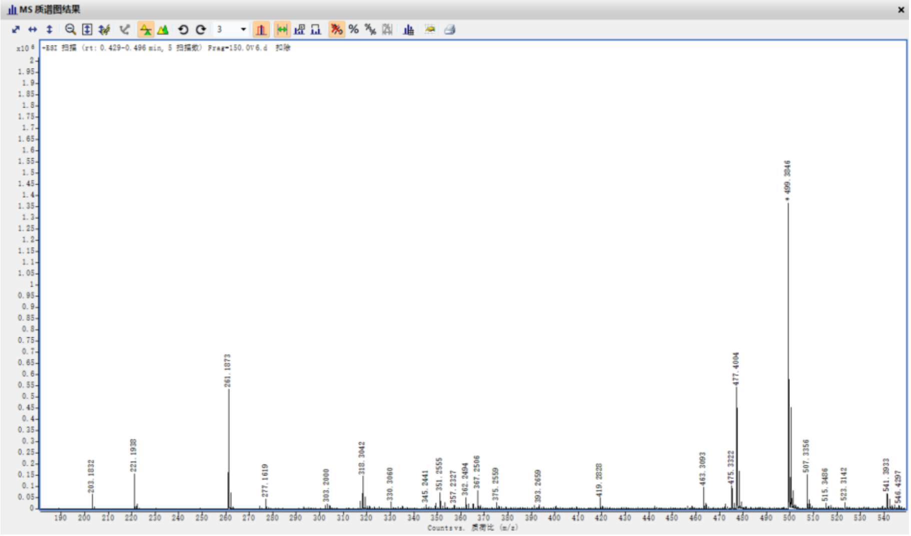
MS 质谱图结果
+ESI 扫描 (rt: 0.429-0.496 min, 5 扫描数) Frag=150.0V 6.d 扣除
x10^6
203.1832
221.1938
261.1873
277.1619
303.2000
318.3042
330.3060
345.2441
351.2555
357.2327
362.2494
367.2506
375.2559
393.2659
419.2828
463.3093
475.3322
477.4004
* 499.3846
507.3356
515.3436
523.3142
541.3933
546.4297
Counts vs. 质荷比 (m/z)

$^{+}$ESI-MS

Compound **25**:

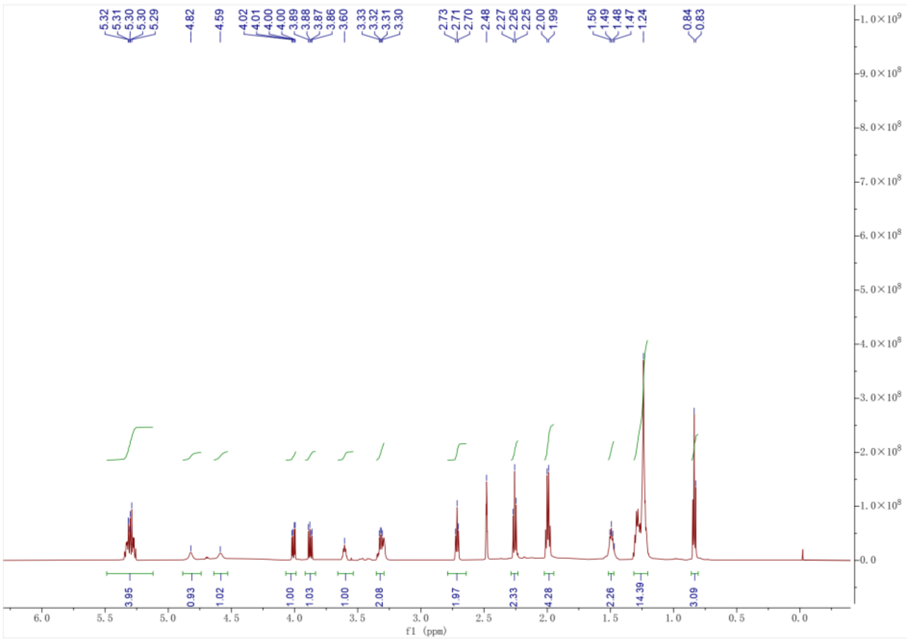

^{1}H-NMR spectrum（DMSO-*d6*, 600 MHz）

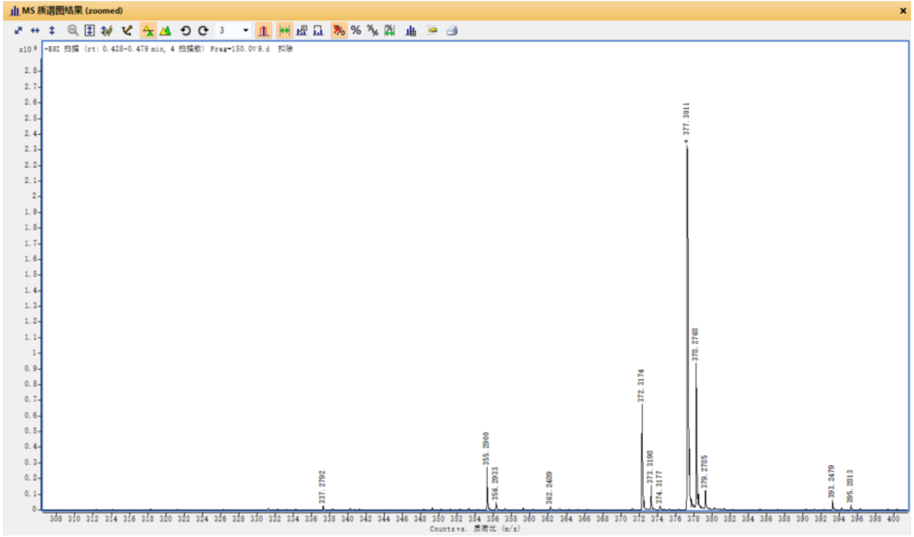

$^{+}$ESI-MS

Compound **26**:

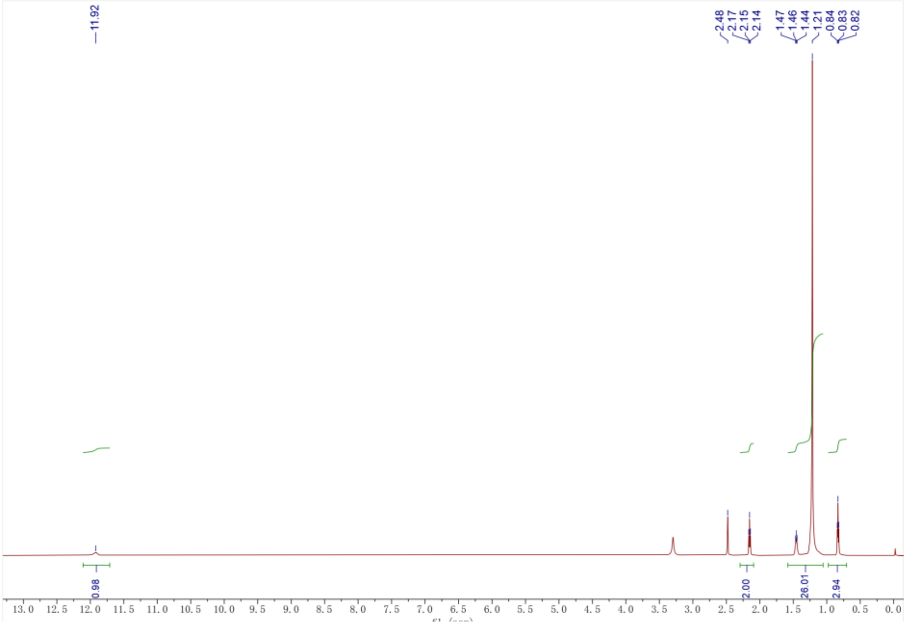

^{1}H-NMR spectrum（DMSO-*d*6, 600 MHz）

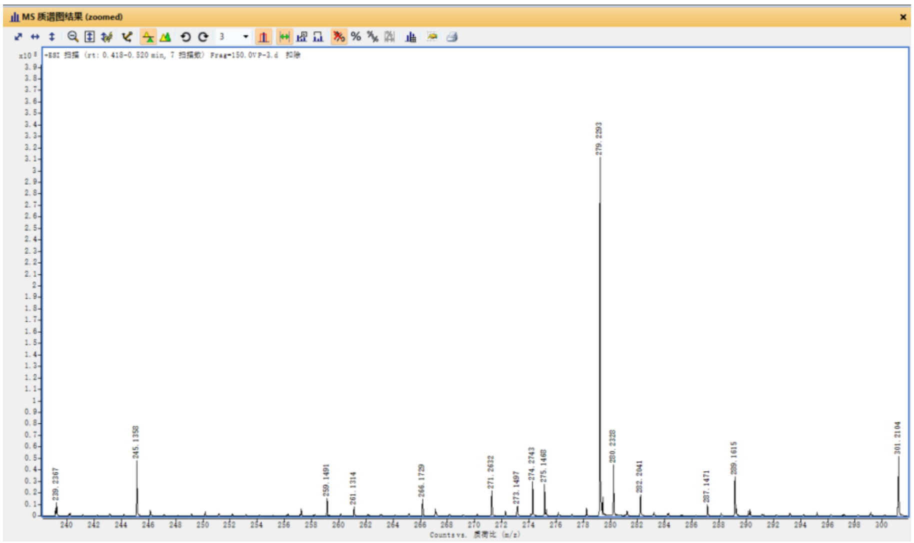

+ESI-MS

Compound **27**:

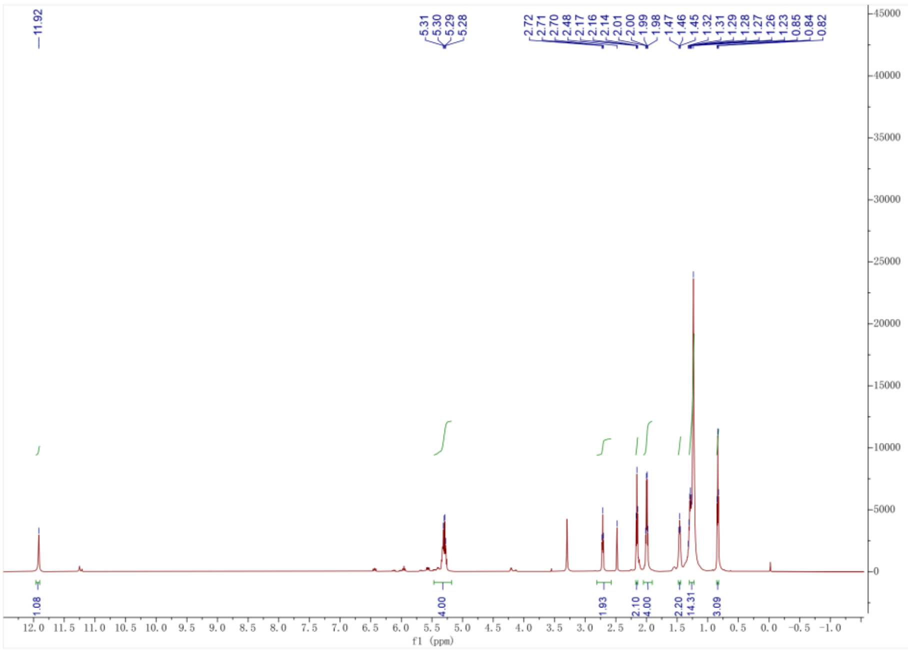

^{1}**H-NMR spectrum（DMSO-*d6*, 600 MHz）**

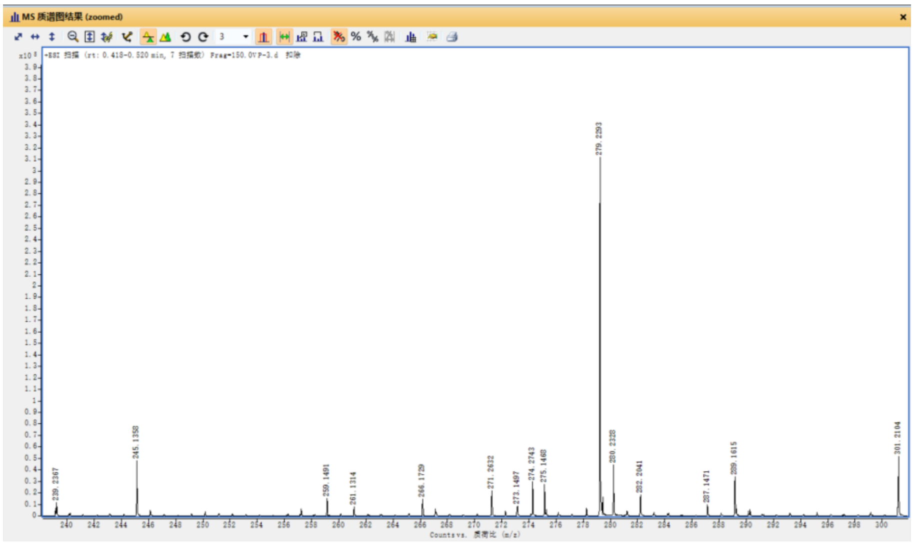

$^{+}$**ESI-MS**

Compound **28**:

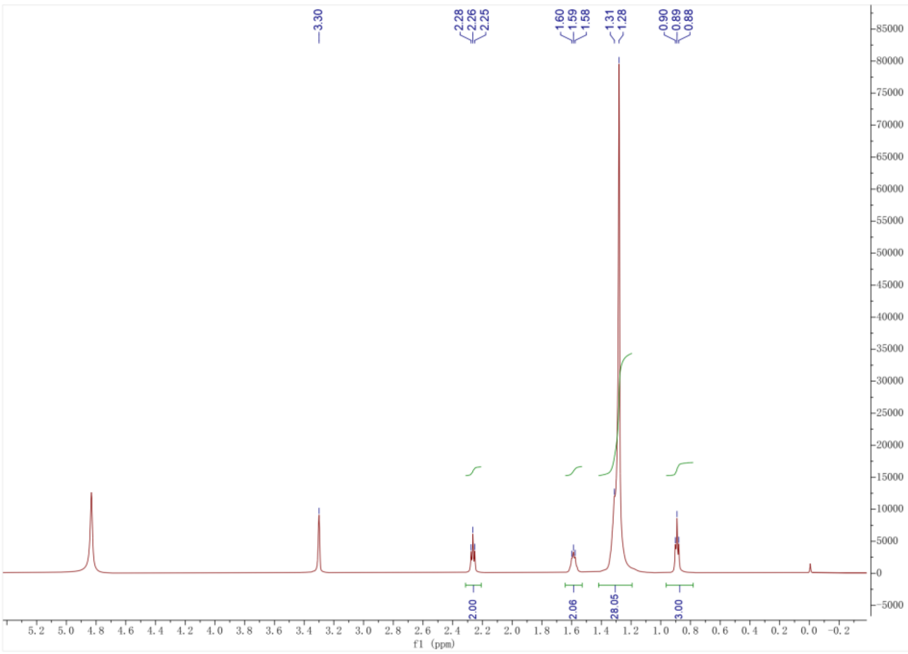

^{1}H-NMR spectrum（CD_3OD, 600 MHz）

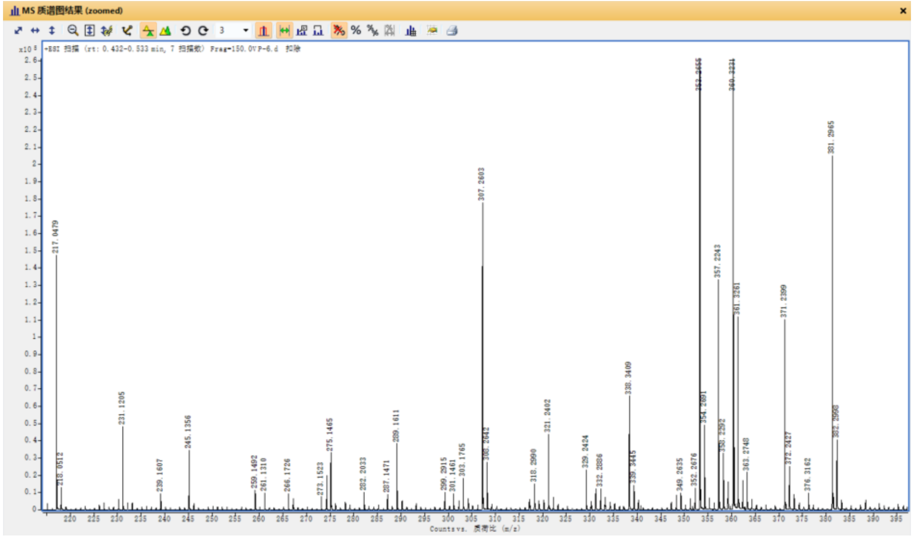

$^{+}$ESI-MS

Compound **29**:

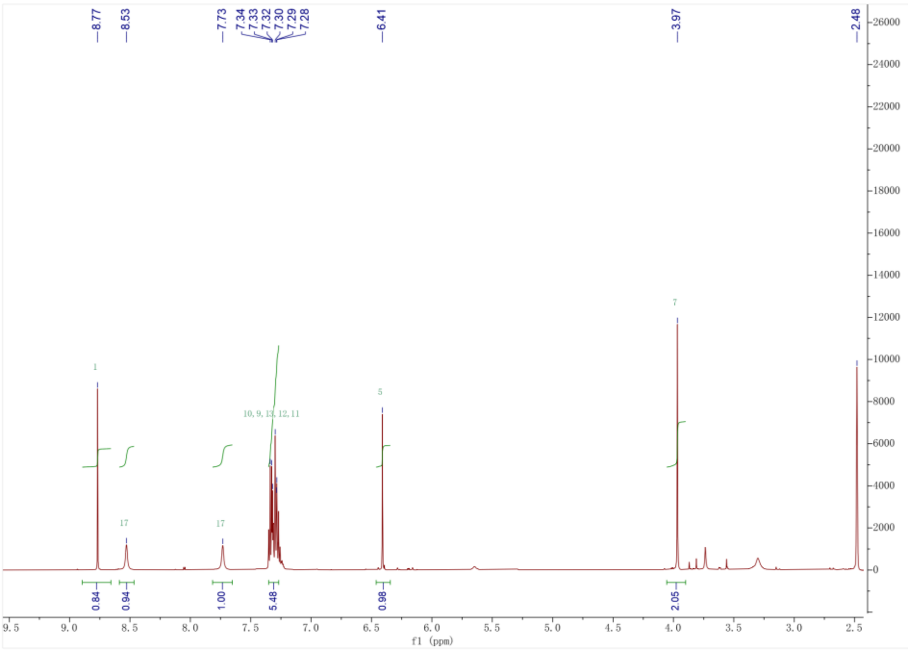

^{1}H-NMR spectrum（DMSO-*d6*, 600 MHz）

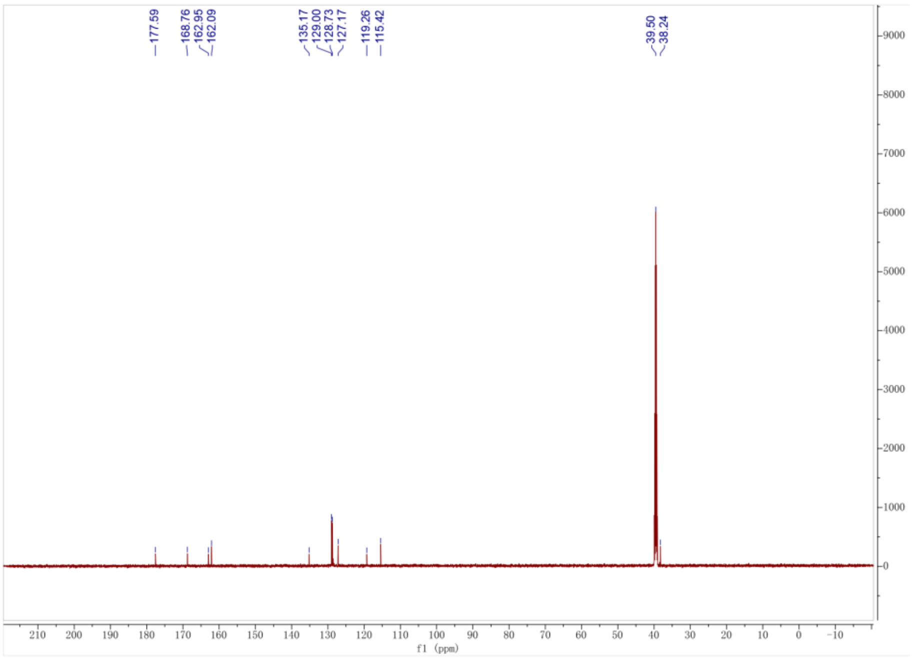

^{13}C-NMR spectrum（DMSO-*d6*, 150 MHz）

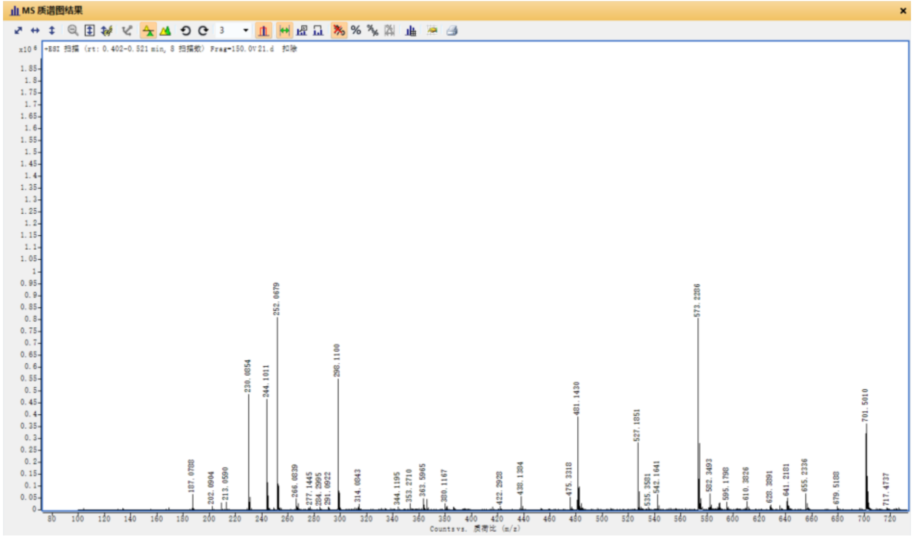
MS 质谱图结果
+ESI 扫描 (rt: 0.402-0.521 min, 8 扫描数) Frag=150.0V 21.d 扣除
x10^6
187.0788
202.0904
213.0590
230.0854
244.1011
252.0679
266.0839
277.1445
284.2995
291.0922
298.1100
314.0843
344.1195
353.2710
363.5965
380.1167
422.2928
438.1384
475.3318
481.1430
527.1851
535.3581
542.1641
573.2286
582.3493
595.1798
610.3826
628.3891
641.2181
655.2336
679.5188
701.5010
717.4737
Counts vs. 质荷比 (m/z)

$^{+}$ESI-MS

Compound **30**：

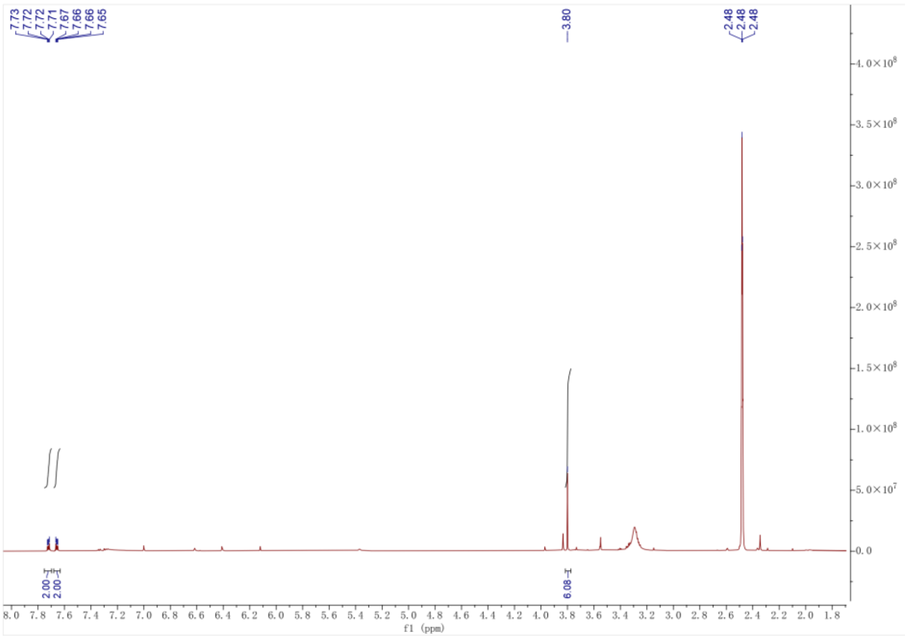

^{1}H-NMR spectrum（DMSO-*d*6, 600 MHz）

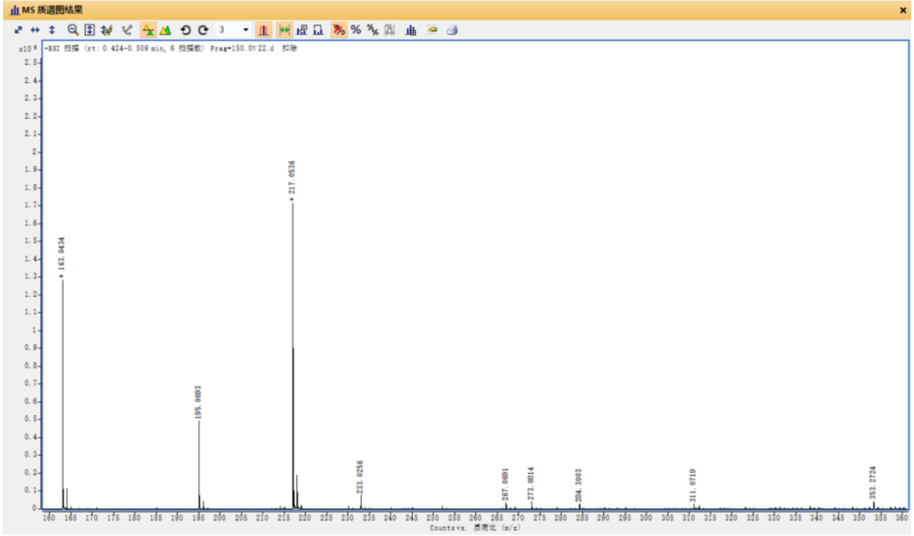

$^{+}$ESI-MS

Compound **31**：

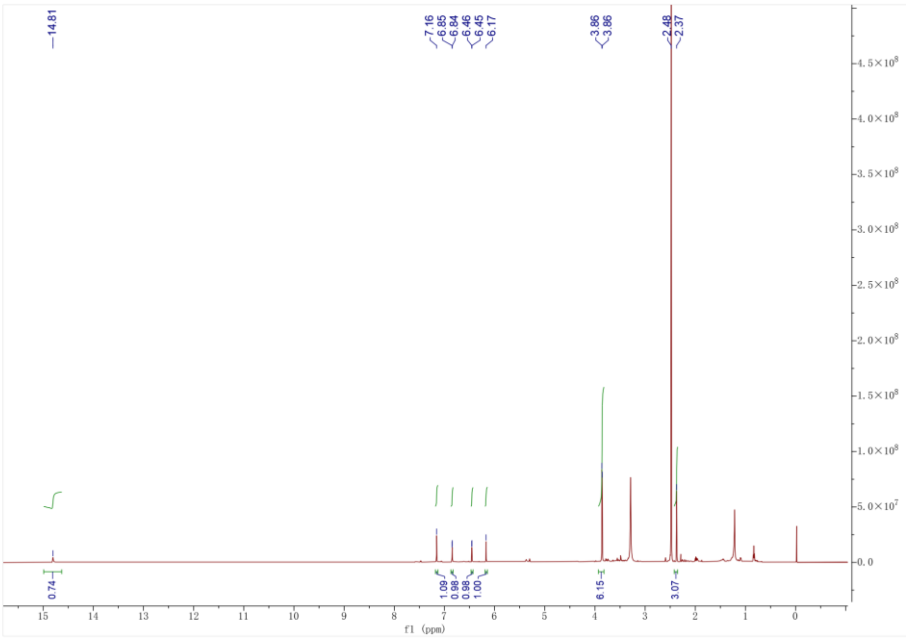

^{1}H-NMR spectrum（DMSO-*d*6, 600 MHz）

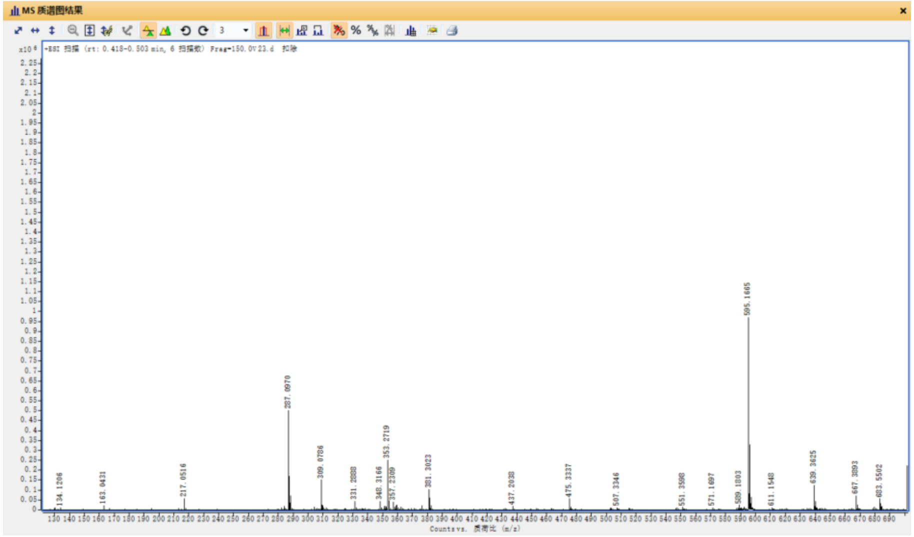

+ESI-MS

Compound **32**:

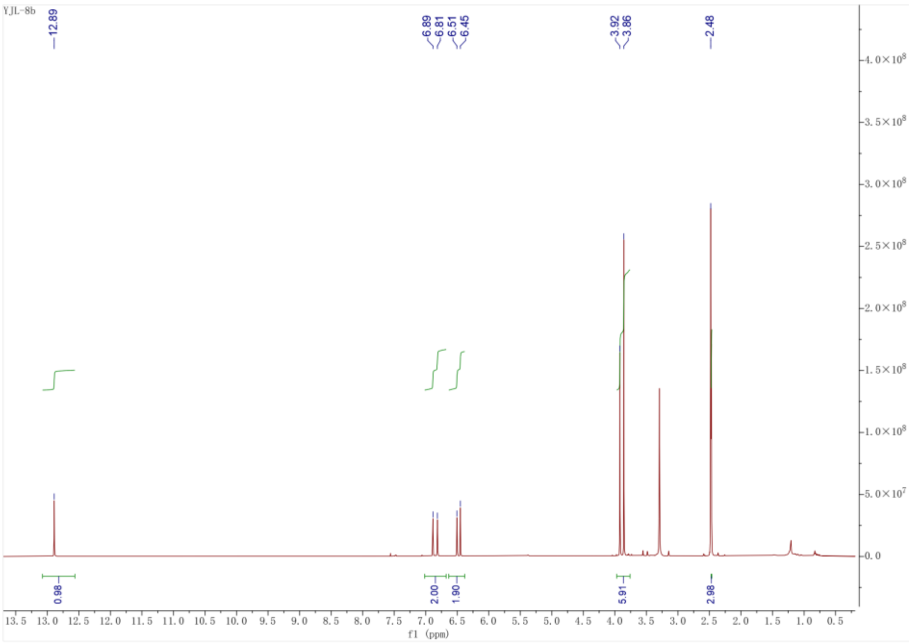

^{1}H-NMR spectrum（DMSO-*d*6, 600 MHz）

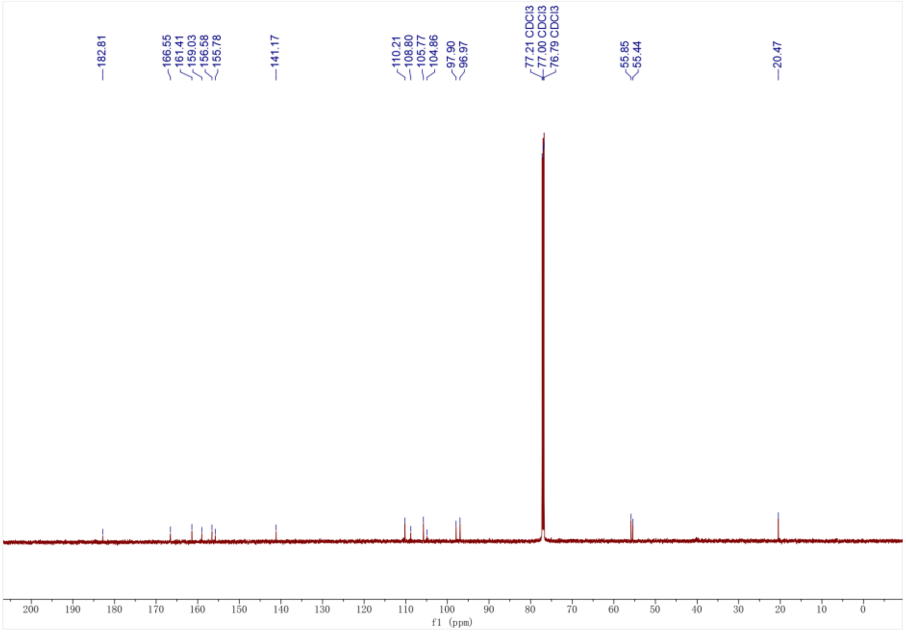

^{13}C-NMR spectrum（$CDCl_3$, 150 MHz）

Compound **33**：

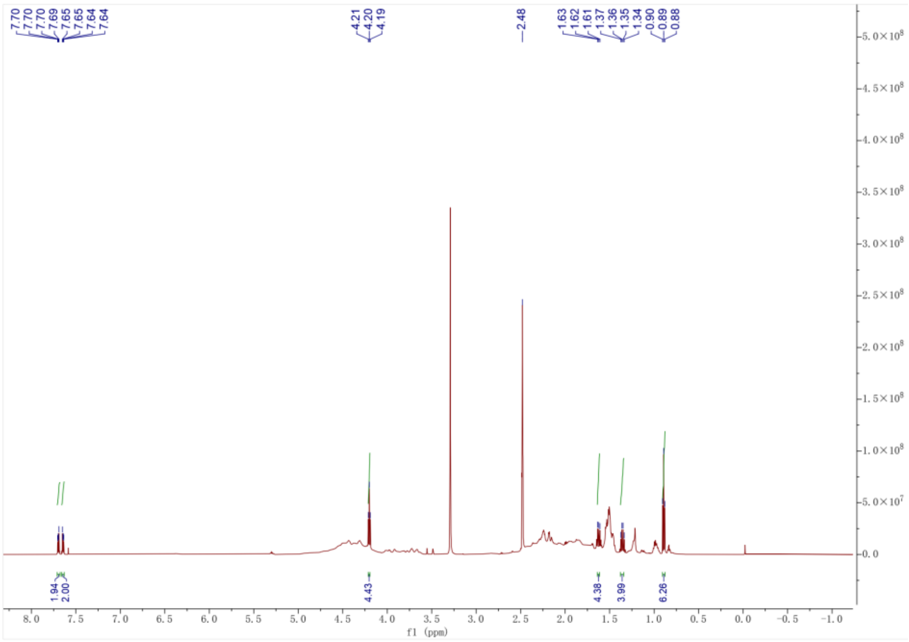

^{1}H-NMR spectrum（DMSO-*d*6, 600 MHz）

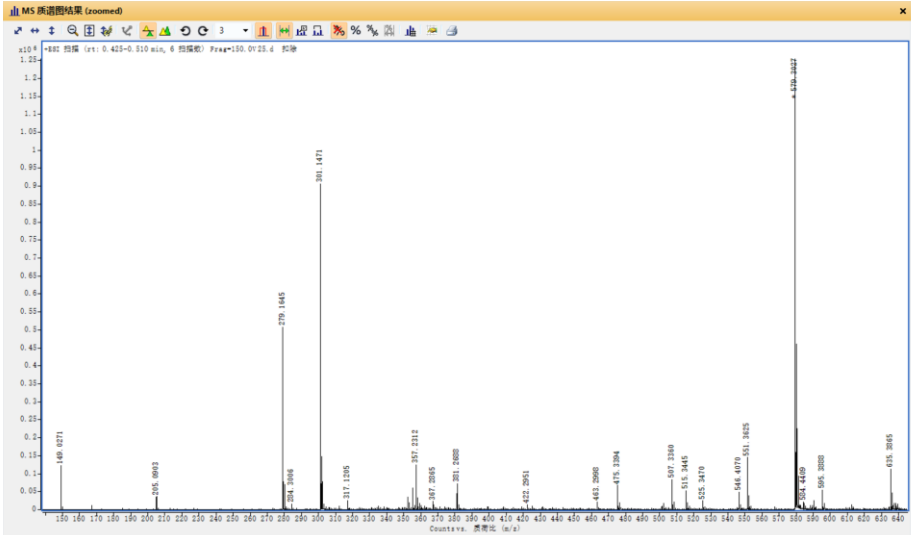

$^+$ESI-MS

Compound **34**：

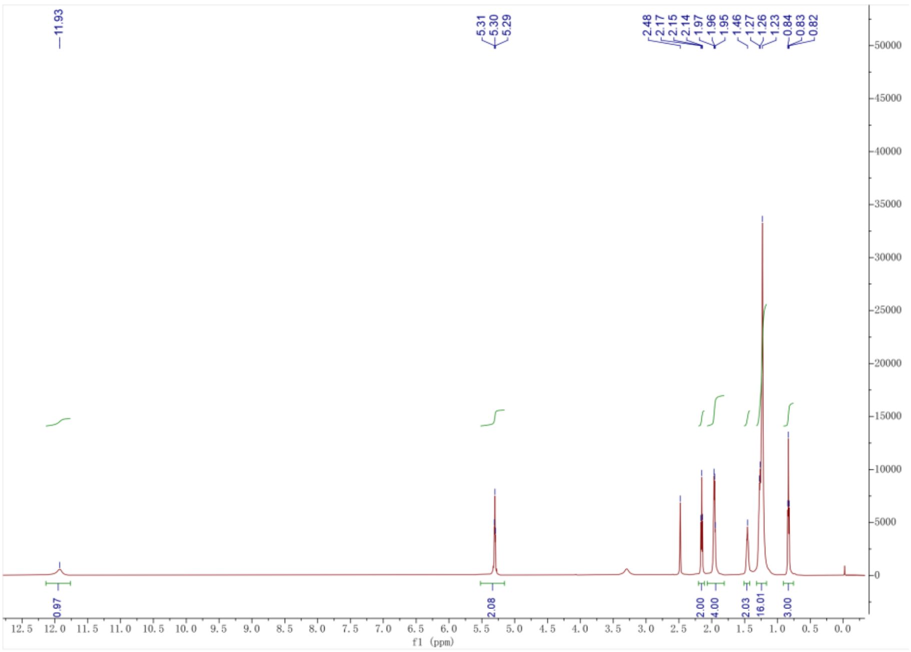

^{1}H-NMR spectrum（DMSO-*d6*, 600 MHz）

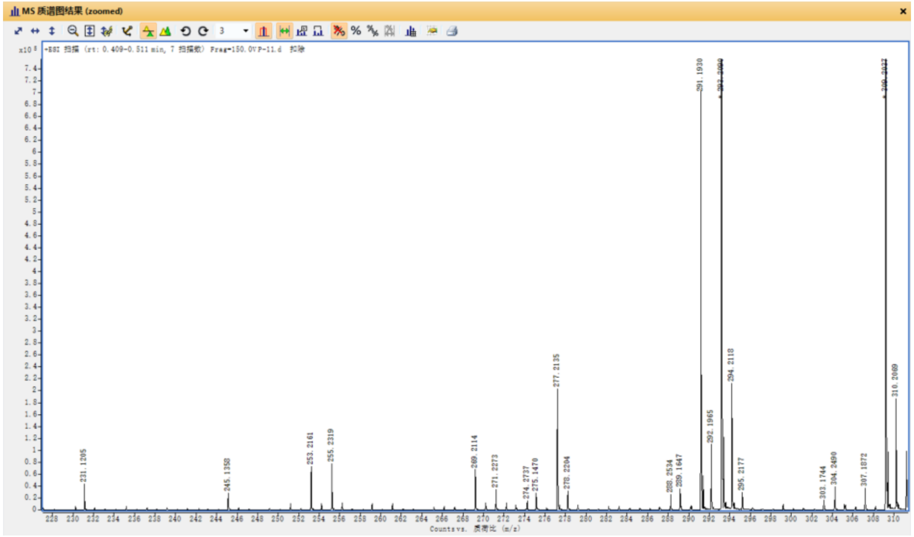

$^{+}$ESI-MS
