# Supplementary material for: From Contact to Stalemate: MAPK-Associated Chemical and Enzymatic Defenses Shape a Stable Barrage in the Co-Culture of Trametes sp. D and Aspergillus niger L14
Source: J Fungi (Basel). 2026 Apr 30;12(5):327. doi: 10.3390/jof12050327 (PMC13208484; doi:10.3390/jof12050327)
Supplement: Supplementary file 1 [file jof-12-00327-s001.zip › Table S2_Reciprocal modulation of major SMs upon exogenous metabolite challenge.pdf]

Table S1. Reciprocal modulation of major SMs upon exogenous metabolite challenge

A. *T.* sp. D metabolite profile after supplementation with *A. niger* L14 SMs

Added *A. niger* L14 SMs	Final conc. (μg/mL)	3-Indoleacetic acid	1H-Indole-3-carboxaldehyde	Tyrosol	1H-Indole-3-acetamide	Cyclo-(Pro-Phe)	Cyclo-(Leu-Pro)	Ergosterol peroxide
Fonsecin	1	0.143±0.09	0.928±0.10	0.697±0.03	0.141±0.06	0.386±0.17	0.200±0.09	0.768±0.16
	10	0.162±0.10	1.176±0.04	0.810±0.06	0.203±0.15	0.764±0.07	0.429±0.05	0.834±0.05
	100	0.321±0.10	1.324±0.10	0.991±0.15	0.278±0.07	1.166±0.03	0.606±0.05	0.962±0.17
Ferulic acid	1	0.140±0.08	0.886±0.06	0.739±0.03	ND	ND	0.002±0.00	0.082±0.07
	10	0.203±0.08	0.894±0.05	0.780±0.08	ND	ND	0.003±0.00	0.758±0.07
	100	0.999±0.18	0.894±0.03	0.792±0.09	ND	ND	0.004±0.00	0.834±0.17
Kojic acid	1	0.194±0.03	0.914±0.02	0.974±0.07	0.180±0.07	0.349±0.04	0.043±0.02	0.251±0.19
	10	0.195±0.11	1.098±0.11	1.046±0.09	0.849±0.05	0.535±0.15	0.067±0.03	0.490±0.18
	100	0.425±0.09	1.173±0.08	1.251±0.03	1.456±0.04	0.670±0.06	0.167±0.06	0.876±0.07
Vanillic acid	1	0.518±0.08	1.158±0.04	0.780±0.07	0.046±0.04	ND	0.024±0.02	ND
	10	0.903±0.10	1.233±0.11	0.810±0.02	0.132±0.13	ND	0.040±0.02	ND
	100	0.960±0.02	1.276±0.06	0.939±0.06	0.890±0.03	ND	0.061±0.04	ND
Veratric acid	1	0.442±0.03	1.755±0.09	0.739±0.09	0.030±0.03	ND	0.048±0.03	ND
	10	0.569±0.06	1.912±0.06	0.963±0.05	0.046±0.04	ND	0.058±0.04	ND
	100	0.665±0.06	1.998±0.05	1.270±0.15	0.141±0.08	ND	0.071±0.06	ND
Caffeic acid	1	0.172±0.04	1.074±0.06	0.788±0.08	0.066±0.05	ND	0.006±0.01	ND
	10	0.202±0.07	1.153±0.02	1.069±0.02	0.107±0.08	ND	0.016±0.01	ND
	100	0.503±0.02	1.766±0.03	1.182±0.06	0.237±0.09	ND	0.076±0.04	ND
5-Hydroxymethyl-2-furfuraldehyde	1	0.141±0.10	0.871±0.09	0.732±0.04	ND	0.191±0.04	0.514±0.16	0.383±0.08
	10	0.142±0.05	0.886±0.09	0.738±0.05	ND	0.262±0.04	0.609±0.05	0.791±0.04
	100	0.143±0.09	0.887±0.08	0.740±0.17	ND	1.002±0.09	0.610±0.04	1.050±0.16
5-Hydroxymethyl-2-furancarboxylic acid	1	0.142±0.07	0.886±0.04	0.743±0.03	ND	0.062±0.05	0.136±0.11	0.383±0.03
	10	0.142±0.07	0.886±0.07	0.795±0.14	ND	0.105±0.04	0.225±0.03	0.459±0.09
	100	0.144±0.05	0.887±0.04	0.833±0.06	ND	0.124±0.03	0.240±0.03	0.741±0.02
Xanthine	1	0.540±0.05	1.287±0.03	0.783±0.11	ND	ND	ND	ND
	10	0.554±0.03	2.200±0.13	0.926±0.16	ND	ND	ND	ND
	100	0.626±0.04	2.737±0.07	1.133±0.03	ND	ND	ND	ND

Added *A. niger* L14 SMs	Final conc. (μg/mL)	3-Indoleacetic acid	1H-Indole-3-carboxaldehyde	Tyrosol	1H-Indole-3-acetamide	Cyclo-(Pro-Phe)	Cyclo-(Leu-Pro)	Ergosterol peroxide
Adenine	1	0.222±0.11	1.179±0.07	0.755±0.04	ND	ND	ND	ND
	10	0.328±0.05	1.384±0.07	1.141±0.03	ND	ND	ND	ND
	100	0.708±0.06	1.713±0.08	1.501±0.07	ND	ND	ND	ND
Nicotinic acid	1	0.193±0.04	1.212±0.05	0.844±0.04	ND	ND	0.144±0.06	0.465±0.03
	10	0.677±0.07	1.245±0.11	0.910±0.02	ND	ND	0.442±0.06	0.512±0.14
	100	0.905±0.11	1.874±0.10	1.479±0.10	ND	ND	0.503±0.08	0.524±0.07
Nicotinamide	1	0.186±0.15	0.997±0.07	0.788±0.07	ND	ND	0.134±0.10	0.243±0.05
	10	0.442±0.11	1.488±0.08	1.019±0.04	ND	ND	0.264±0.17	0.569±0.03
	100	0.251±0.06	1.676±0.02	1.159±0.05	ND	ND	0.341±0.08	0.785±0.06
12 *A. niger* L14-derived SM mixture	mixture	1.250±0.03	1.745±0.10	1.291±0.08	1.132±0.09	0.587±0.05	0.670±0.12	0.623±0.04

B. *A. niger* L14 metabolite profile after supplementation with *T.* sp. D SMs

Added *T.* sp. D SMs	Final conc. (μg/mL)	Fonsecin	Ferulic acid	Kojic acid	Vanillic acid	Veratric acid	Caffeic acid	5-Hydroxymethyl-2-furfuraldehyde	5-Hydroxymethyl-2-furancarboxylic acid	Xanthine	Adenine	Nicotinic acid	Nicotinamide
3-Indoleacetic acid	1	1.200±0.05	0.966±0.04	0.408±0.03	0.856±0.17	0.638±0.05	0.490±0.16	ND	ND	ND	0.161±0.09	ND	ND
	10	1.980±0.14	1.402±0.10	0.516±0.17	1.311±0.03	0.951±0.16	0.537±0.06	ND	ND	ND	0.429±0.03	ND	ND
	100	2.895±0.05	1.721±0.04	0.540±0.06	1.511±0.04	1.025±0.03	0.735±0.09	ND	ND	ND	0.576±0.09	ND	ND
1H-Indole-3-carboxaldehyde	1	1.129±0.10	0.939±0.05	0.406±0.04	0.937±0.09	0.614±0.10	0.594±0.08	0.152±0.04	0.087±0.02	0.046±0.05	0.048±0.04	0.025±0.02	0.080±0.08
	10	1.724±0.06	1.634±0.11	0.437±0.04	1.008±0.06	0.650±0.05	0.652±0.03	0.178±0.07	0.109±0.11	0.100±0.03	0.096±0.05	0.028±0.03	0.100±0.10
	100	3.096±0.09	2.516±0.05	0.459±0.08	1.089±0.15	0.727±0.06	0.735±0.15	0.281±0.09	0.138±0.08	0.115±0.10	0.161±0.03	0.041±0.04	0.129±0.06
Tyrosol	1	0.935±0.04	1.045±0.03	0.434±0.05	0.825±0.07	0.620±0.07	0.624±0.17	ND	ND	ND	ND	ND	ND
	10	1.387±0.06	1.545±0.04	0.443±0.09	0.966±0.04	0.643±0.08	0.800±0.14	ND	ND	ND	ND	ND	ND
	100	1.853±0.07	1.806±0.09	0.555±0.08	1.151±0.07	1.015±0.09	0.917±0.02	ND	ND	ND	ND	ND	ND

Added *T.* sp. D SMs	Final conc. (μg/mL)	Fonsecin	Ferulic acid	Kojic acid	Vanillic acid	Veratric acid	Caffeic acid	5-Hydroxymethyl-2-furfuraldehyde	5-Hydroxymethyl-2-furancarboxylic acid	Xanthine	Adenine	Nicotinic acid	Nicotinamide
1H-Indole-3-acetamide	1	1.675±0.06	0.834±0.07	0.524±0.06	0.923±0.05	0.803±0.04	0.529±0.10	ND	ND	ND	0.205±0.08	ND	ND
	10	2.013±0.09	1.508±0.02	0.634±0.06	1.334±0.02	0.840±0.06	0.691±0.06	ND	ND	ND	0.303±0.09	ND	ND
	100	2.802±0.07	3.203±0.06	0.715±0.05	1.485±0.17	0.979±0.07	0.882±0.19	ND	ND	ND	0.684±0.11	ND	ND
Cyclo-(Pro-Phe)	1	1.209±0.05	0.877±0.07	0.442±0.07	0.957±0.02	0.665±0.03	0.509±0.07	0.185±0.10	0.135±0.04	0.060±0.06	0.024±0.02	0.095±0.09	0.018±0.02
	10	2.525±0.08	1.314±0.03	0.503±0.14	1.344±0.09	0.729±0.04	0.791±0.04	0.229±0.03	0.193±0.19	0.132±0.03	0.067±0.04	0.211±0.10	0.059±0.03
	100	2.917±0.03	3.611±0.08	0.714±0.04	1.512±0.03	0.869±0.06	0.951±0.07	0.299±0.11	0.240±0.05	0.183±0.08	0.522±0.10	0.642±0.09	0.084±0.06
Cyclo-(Leu-Pro)	1	1.532±0.15	1.344±0.08	0.327±0.18	0.953±0.04	0.776±0.05	0.553±0.10	0.131±0.09	0.120±0.05	0.196±0.04	0.174±0.10	0.107±0.07	0.094±0.07
	10	3.419±0.06	2.718±0.05	0.623±0.09	1.342±0.08	0.820±0.07	0.574±0.11	0.147±0.02	0.127±0.03	0.245±0.03	0.398±0.04	0.117±0.07	0.262±0.06
	100	4.142±0.18	4.095±0.03	0.883±0.02	1.461±0.08	0.835±0.08	0.804±0.05	0.227±0.09	0.146±0.06	0.412±0.05	0.577±0.07	0.198±0.08	0.299±0.04
Ergosterol peroxide	1	1.755±0.04	1.070±0.12	0.475±0.03	1.311±0.08	0.834±0.08	0.629±0.08	0.524±0.07	0.058±0.04	0.101±0.03	0.207±0.02	0.274±0.02	0.091±0.08
	10	2.592±0.08	1.375±0.03	0.630±0.09	1.861±0.12	1.059±0.20	0.997±0.09	0.699±0.09	0.127±0.06	0.500±0.11	0.435±0.04	0.651±0.18	0.102±0.05
	100	4.944±0.05	2.655±0.19	0.808±0.04	2.181±0.06	1.275±0.09	1.073±0.06	1.399±0.08	0.535±0.09	0.540±0.07	0.832±0.07	0.672±0.08	0.381±0.07
7 *T.* sp. D-derived SM mixture	mixture	2.067±0.04	1.593±0.09	0.594±0.19	1.922±0.04	0.830±0.03	0.685±0.18	1.098±0.06	0.353±0.08	0.842±0.09	0.661±0.10	0.295±0.04	0.214±0.03

Notes: Report values as Mean ± SD (n = 3). ND, not detected.
