# Supplementary material for: From Contact to Stalemate: MAPK-Associated Chemical and Enzymatic Defenses Shape a Stable Barrage in the Co-Culture of Trametes sp. D and Aspergillus niger L14
Source: J Fungi (Basel). 2026 Apr 30;12(5):327. doi: 10.3390/jof12050327 (PMC13208484; doi:10.3390/jof12050327)
Supplement: Supplementary file 1 [file jof-12-00327-s001.zip › Table S3_Molecular docking results of seven T. sp. D-derived SMs with A. niger.pdf]

Molecular docking results of seven *T.* sp. D-derived SMs with *A. niger*

Enzymes			Name	3-indoleacetic_acid	1H-indole-3-acetamide	1H-indole-3-carboxaldehyde	Cyclo-(Pro-Phe)	Cyclo-(Leu-Pro)	Ergosterol peroxide	Tyrosol
Experimental	transferase（1）	Flavin prenyltransferase PAD1, mitochondrial	6QLG-PadA1	-8.6	-8.6	-7.4	-9.2	-7.8	-10.1	-6.8
	Oxidoreductase（7）	Glucose oxidase	1CF3	-7.2	-7.1	-6.7	-9.0	-7.6	-9.9	-6.3
			3QVP-C2221	-7.2	-7.8	-6.8	-9.5	-7.9	-9.3	-6.4
			3QVR-P3121	-7.2	-7.2	-6.9	-9.5	-8.0	-9.5	-6.3
			8JPZ-Gox_M8	-7.1	-7.0	-6.3	-8.8	-7.0	-10.7	-6.3
		Monoamine oxidase N	2VVL-MAO-N-D3	-8.1	-8.3	-7.3	-11.0	-7.7	-11.5	-6.9
			2VVM-MAO-N-D5	-8.2	-8.2	-7.2	-9.7	-7.9	-10.5	-6.7
		Multicopper oxidase	5LM8-McoG	-6.6	-6.8	-5.9	-7.0	-6.6	-7.9	-5.7
	Lysing enzyme（5）	2,3-dimethylmalate lyase	3FA4	-7.2	-7.2	-6.4	-7.9	-6.9	-10.0	-5.8
		Actibind T2	3TBJ	-6.1	-6.3	-5.7	-7.2	-6.0	-8.1	-5.3
		Ferulic acid decarboxylase 1	6TIH-Fdc	-6.7	-7.0	-6.6	-8.4	-6.7	-9.6	-6.1

		Pectin Lyase A	1IDK	-5.3	-5.4	-4.7	-6.2	-5.4	-7.5	-4.5
		Pectin Lyase B	1QCX	-6.2	-6.1	-5.7	-6.6	-5.6	-7.8	-5.1
	hydrolytic enzymes（23）	Chitinase	6IGY	-6.9	-6.7	-6.1	-7.8	-6.5	-9.1	-5.8
		Endoglucanase A	1KS5	-5.9	-6.0	-5.2	-7.0	-5.8	-8.3	-4.6
		Epoxide hydrolase	1QO7	-6.7	-6.9	-6.1	-7.8	-6.5	-8.6	-5.9
		3-phytase A	3K4P	-6.7	-6.9	-5.8	-7.3	-6.1	-8.7	-5.1
		EstA αβ	1UKC-EstA	-5.6	-5.8	-4.9	-7.3	-6.1	-8.8	-4.8
		Carboxylic ester hydrolase	7K4O	-6.9	-6.2	-6.0	-7.0	-6.2	-7.6	-5.2
		Alpha-L-rhamnosidase	8IWD-Rha-2	-7.4	-7.5	-7.1	-8.8	-7.1	-9.4	-6.5
		Polygalacturonase II	1CZF	-5.6	-5.2	-4.8	-6.1	-5.5	-7.6	-4.6
		rutinosidase	6I1A	-7.2	-7.0	-6.2	-8.0	-6.9	-8.9	-5.7
		Feruloyl Esterase A	1UZA	-6.4	-6.4	-5.8	-7.1	-6.5	-8.1	-5.6
		Isopullulanase	1X0C	-6.6	-6.7	-5.7	-7.5	-6.5	-9.9	-5.2
		Endo-beta-1, 4-glucanase	5I78	-7.1	-7.0	-6.4	-7.6	-6.4	-9.1	-5.7
		Glucoamylase	3EQA	-7.4	-7.5	-6.8	-8.0	-6.9	-9.2	-5.9
			6FRV	-6.4	-6.5	-5.4	-8.6	-6.0	-7.8	-5.8
		Endo-beta-1,4-mannanase	3WH9-ManBK	-6.7	-6.7	-5.9	-8.3	-6.9	-11.0	-5.8
		Polygalacturonase I	1NHC	-6.8	-6.8	-6.2	-8.7	-6.6	-9.4	-5.5

		pH 2.5 acid phosphatase	1QFX	-6.9	-6.9	-6.0	-8.2	-7.3	-9.6	-5.8
		ochratoxinase	4C65	-7.4	-7.5	-7.0	-8.0	-6.7	-9.1	-6.3
		alpha-Amylase	2AAA	-6.2	-6.2	-5.6	-6.8	-5.9	-8.7	-6.0
		Endo-1,4-β-xylanase	1UKR	-7.0	-6.9	-6.5	-8.4	-6.9	-9.1	-6.3
			6QE8-GH11	-6.5	-6.7	-6.1	-8.5	-6.5	-9.2	-5.5
		Actibind T2 RNase	3D3Z	-6.7	-6.4	-5.9	-7.4	-5.8	-8.9	-5.3
		Glycosyl hydrolases family 31 family protein	6DRU	-7.0	-7.1	-6.5	-8.8	-6.8	-10.3	-6.0
Predicted	Oxidoreductase（1）	Aldehyde dehydrogenase	AF_AFP41751F1	-7.0	-7.0	-6.8	-8.0	-6.1	-9.0	-5.8
	hydrolytic enzymes (2)	Exopolygalacturonase X	AF_AFQ27UB3F1	-6.4	-6.4	-5.6	-7.5	-6.9	-7.8	-5.4
		Pectinesterase	AF_AFP17872F1	-5.5	-5.2	-5.1	-6.1	-5.4	-7.4	-4.9
