# Supplementary material for: From Contact to Stalemate: MAPK-Associated Chemical and Enzymatic Defenses Shape a Stable Barrage in the Co-Culture of Trametes sp. D and Aspergillus niger L14
Source: J Fungi (Basel). 2026 Apr 30;12(5):327. doi: 10.3390/jof12050327 (PMC13208484; doi:10.3390/jof12050327)
Supplement: Supplementary file 1 [file jof-12-00327-s001.zip › Table S4_Molecular docking results of twelve A. niger L14-derived SMs with T. versicolor.pdf]

Molecular docking results of twelve *A. niger* L14-derived SMs with *T. versicolor*

Enzymes			Name	5-hydroxymethyl-2-furancarboxylic acid	5-hydroxymethyl-2-furfuraldehyde	Ferulic acid	Fonsecin	Kojic acid	Vanillic acid	Veratric acid	Caffeic acid	Nicotinamide	Nicotinic acid	Adenine	Xanthine
Experemental	Oxidoreductase（4）	Laccase	1KYA	-5.7	-5.0	-6.3	-8.1	-6.2	-6.5	-6.3	-7.1	-5.9	-5.9	-6.0	-6.3
		Laccase 2	1GYC	-5.8	-5.0	-6.4	-7.7	-5.9	-6.2	-6.1	-6.7	-5.6	-5.6	-5.5	-6.2
		Hydroxyquinol-1,2-dioxygenase	8R2W	-5.7	-5.4	-6.9	-7.6	-5.9	-6.6	-6.2	-7.1	-6.1	-6.0	-5.8	-7.1
		Phenol 2-monooxygenase	8R2U	-5.5	-5.3	-6.9	-8.8	-5.4	-6.2	-6.3	-7.0	-5.9	-5.7	-5.8	-6.7
	转移酶（8）	glutathione transferase	6GC9-glutathione transferase Xi 1 (3.2 Å)	-5.6	-5.3	-6.8	-9.4	-5.7	-6.0	-6.0	-6.9	-5.3	-5.2	-6.2	-6.9
			6GCA-glutathione transferase Xi 3	-5.6	-5.2	-7.0	-7.9	-5.8	-6.4	-6.4	-7.2	-5.7	-5.5	-6.4	-6.5
			6GIB-glutathione transferase Omega 2S	-5.3	-4.6	-6.5	-8.4	-5.0	-5.9	-5.7	-6.3	-5.1	-5.1	-5.5	-5.9
			6F43-glutathione transferase Omega 3S	-5.9	-5.4	-7.0	-8.8	-5.5	-6.4	-6.7	-7.3	-5.7	-5.7	-6.2	-6.1
			6F70-glutathione transferase Omega 6S	-5.0	-5.1	-6.6	-7.7	-4.8	-5.9	-5.7	-6.5	-5.0	-5.0	-5.1	-5.9
			6HJS-glutathione transferase Omega 1C	-4.8	-4.5	-6.0	-7.2	-5.0	-5.5	-5.3	-6.0	-4.7	-4.9	-4.8	-5.3
			6SR9-glutathione transferase Omega 2C	-5.3	-4.8	-5.9	-8.1	-5.0	-5.7	-5.5	-6.5	-4.9	-4.9	-5.3	-5.7
			6SRB-glutathione transferase Omega 3C	-6.5	-5.9	-6.5	-7.7	-5.9	-6.2	-5.6	-6.5	-5.8	-6.0	-6.4	-6.4
Predicted	Oxidoreductase（4）	Laccase	AF_AFQ12719F1-Laccase 4	-5.4	-4.8	-6.8	-8.2	-5.2	-6.2	-5.8	-6.6	-5.3	-5.1	-5.5	-5.9
			AF_AFQ12717F1-Laccase 5	-5.6	-4.7	-6.0	-8.2	-5.6	-6.1	-5.9	-6.7	-5.5	-5.4	-5.7	-6.1

		Ligninase C	AF_AFP20013F1	-5.5	-5.0	-6.9	-9.3	-5.2	-6.1	-6.4	-6.8	-5.6	-5.4	-5.3	-5.7
		Pyranose 2-oxidase	AF_AFP79076F1	-5.9	-5.5	-7.3	-8.7	-5.8	-6.9	-6.3	-7.4	-5.9	-5.7	-6.0	-6.3
