# Supplementary figures and images for: From Contact to Stalemate: MAPK-Associated Chemical and Enzymatic Defenses Shape a Stable Barrage in the Co-Culture of Trametes sp. D and Aspergillus niger L14
Source: J Fungi (Basel). 2026 Apr 30;12(5):327. doi: 10.3390/jof12050327 (PMC13208484; doi:10.3390/jof12050327)

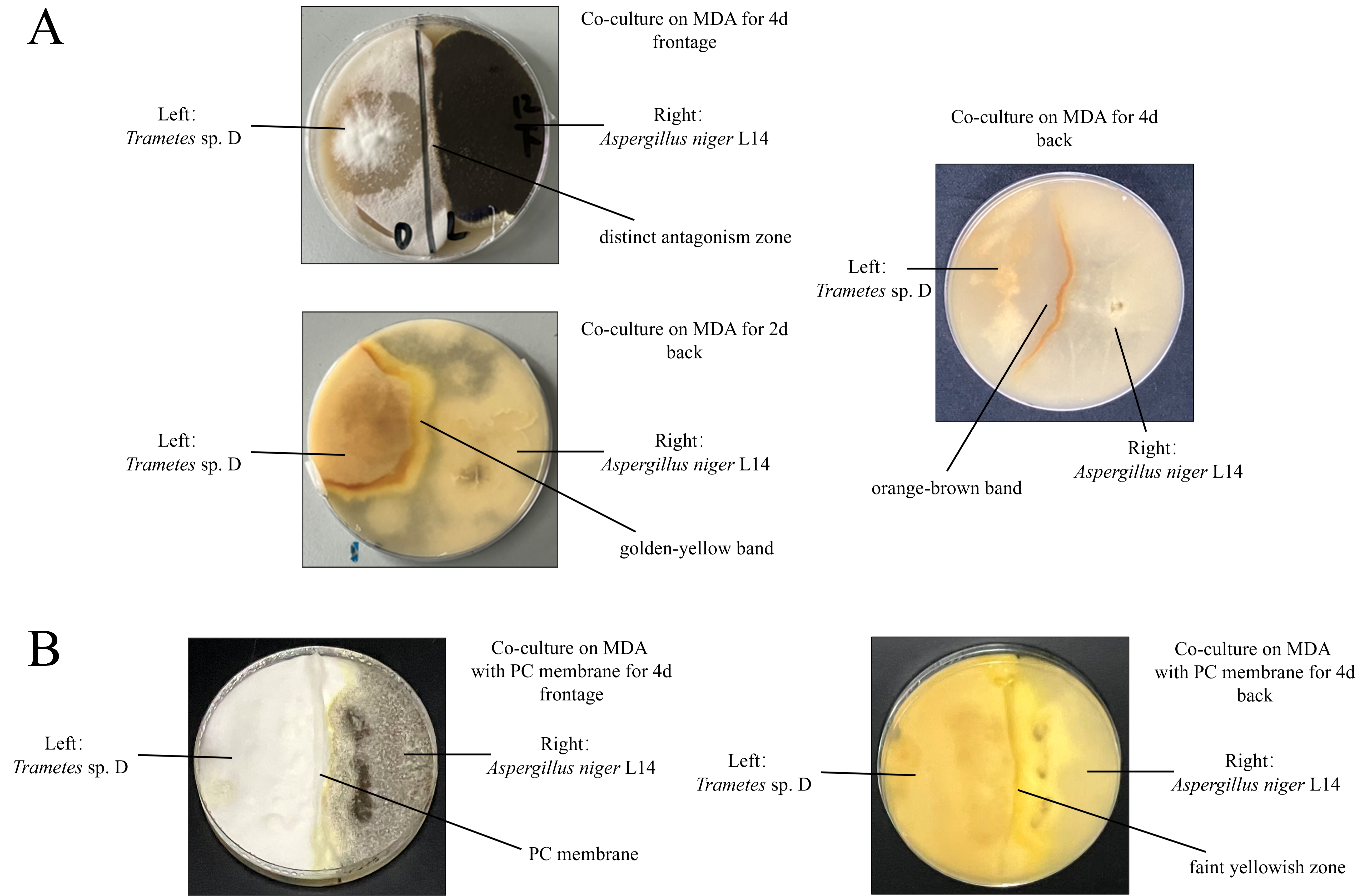

Supplement: Supplementary file 1 [file jof-12-00327-s001.zip › Figure S1_Time-resolved macroscopic phenotypes of confrontation and the effect of physical separation.tif]

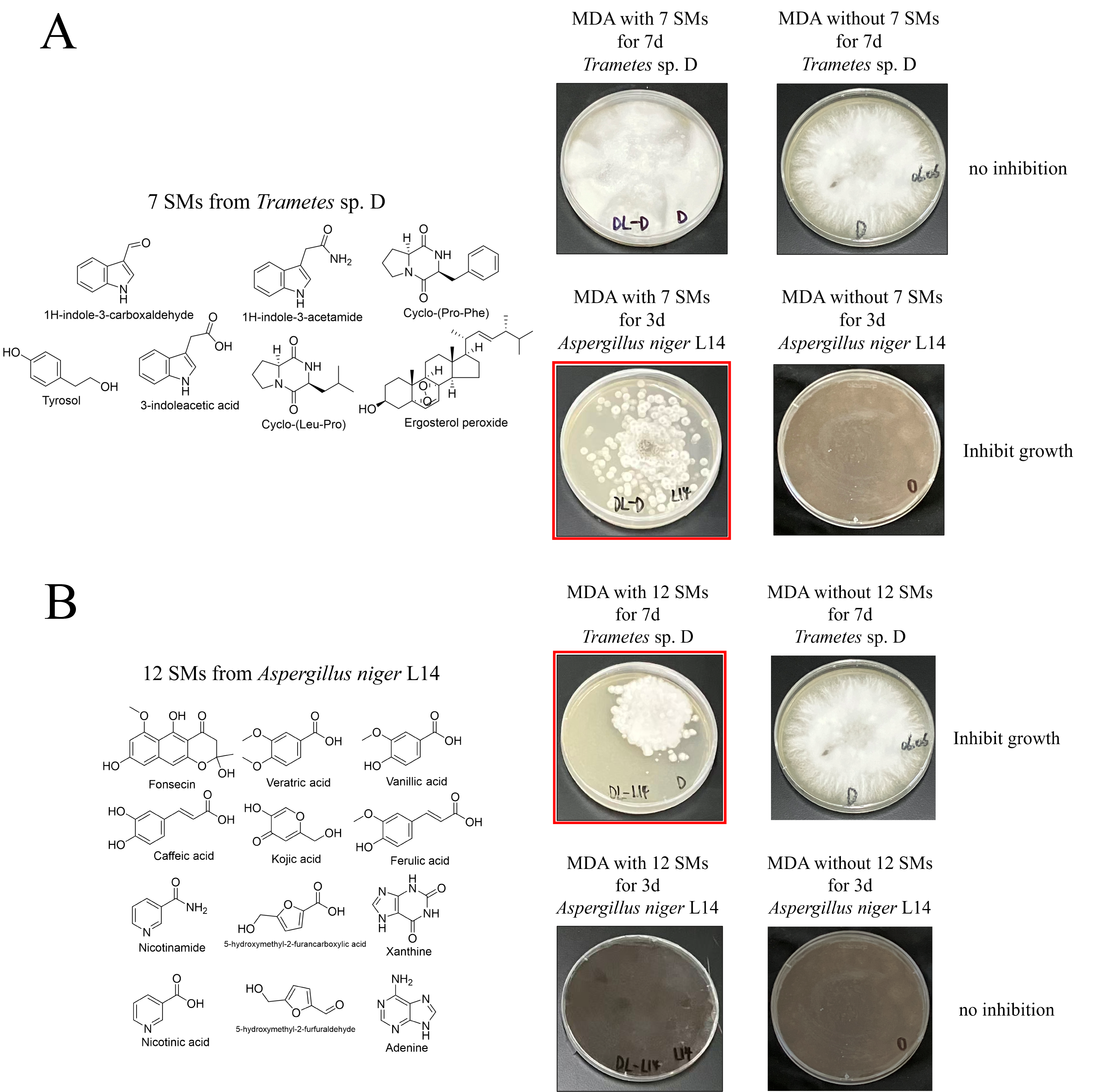

Supplement: Supplementary file 1 [file jof-12-00327-s001.zip › Figure S3_Plate assays using mixtures of all identified SMs demonstrate selective antagonism without self-inhibition.tif]
